# Design, Synthesis and ROMP of Novel *Exo*-Norbornene Silyl Ethers for Functional Polymer Applications

**DOI:** 10.3390/ma19091681

**Published:** 2026-04-22

**Authors:** Mariusz Majchrzak, Jerzy Garbarek, Ahmed M. Eissa

**Affiliations:** 1 Department of Organometallic Chemistry, Faculty of Chemistry, Adam Mickiewicz University in Poznań, 8 Uniwersytetu Poznańskiego Str., 61-614 Poznań, Poland; jerzy.garbarek@amu.edu.pl; 2 Research Institute of Healthcare Sciences, School of Pharmacy & Life Sciences, Faculty of Science and Engineering, University of Wolverhampton, Wolverhampton WV1 1LY, UK; a.m.eissa@wlv.ac.uk; 3 Department of Chemistry, School of Engineering, University of Warwick, Coventry CV4 7AL, UK; 4 Department of Polymers, Chemical Industries Research Division, National Research Centre, El-Bohouth St. 33, Dokki, Giza 12622, Cairo, Egypt

**Keywords:** Grubbs catalyst, ROMP, norbornene, silyl-ether, silicon, polymeric material

## Abstract

With the constant development of new polymer chemistry technologies, it is necessary to find modern synthetic pathways for the synthesis of polymers bearing numerous applicable characteristics, in an easy, efficient and environmentally friendly way. One such possibility is to present the use of metathesis type reactions and more specifically ring-opening metathesis polymerisation (ROMP), which provides the opportunity to produce linear unsaturated functionalised polymeric chains in a ‘living’ yet controlled manner with the use of ruthenium-based carbene (Ru=CHR) Grubbs’ catalysts (initiators: G1, G2, G3). In order to achieve satisfying results and obtain full conversion of the monomers, sterically hindered molecules are preferred, because the process of opening the ring results in simultaneous release of the energy that propagates the whole process. The incorporation of silicon-based substituents (such as silyl ethers) into the norbornene matrix can provide higher thermal stability of polymers, leading to the creation of flame-retardant materials. Other applications include gas separation membranes or biomedicine, upon further modification. This paper focusses on the development and optimisation of the synthetic method of previously not reported *exo*-norbornene silyl ethers along with their metathesis polymerisation to achieve linear unsaturated polymers with high isolation yields.

## 1. Introduction

**Scheme 1:**
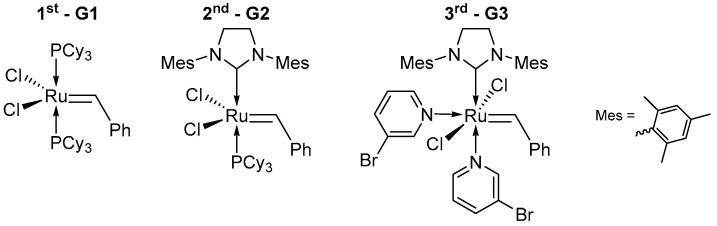
Structures of Grubbs’ generations catalysts (G1, G2, G3).

Olefin metathesis, a metal-catalysed method for redistributing double carbon–carbon bonds, has been known since the mid-1950s [1], and the idea was further developed in the 1960s [2]. Today, it remains an efficient approach for obtaining unsaturated molecules that are often challenging or impossible to synthesise using other methods. Given the constant demand for new modern technologies and innovative polymer synthesis techniques, ring-opening metathesis polymerisation (ROMP) has emerged as a powerful tool in controllable polymer chemistry. ROMP enables the creation of a diverse range of polymers with unique, complex architectures, varied compositions, and useful functionalities. ROMP converts cyclic olefins into linear polymers, incorporating olefin moieties in the main chain, using organometallic catalysts in a ‘*living*’ fashion, allowing the creation of block-type copolymers. To achieve this, catalysts that control the structure, molecular weight, and physical and chemical properties along the polymer chains are essential [3]. In the past, various initiators composed of transition metal complexes, such as Ti [4], Ta [5], W, and Mo [6], were employed. However, in recent times, ruthenium carbene-type catalysts (Grubbs’ catalysts) (Scheme 1) have been used predominantly due to their stability in air and compatibility with different functional groups [7]. For successful ROMP, sterically hindered compounds, such as cyclooctene, dicyclopentadiene, or norbornene, are required [3]. With all of these characteristics combined, ROMP allows for fast polymerisation with high macromonomer conversion, facile incorporation of a variety of functional polymers into frameworks, and precise control over the macromolecular architecture by independently manipulating the lengths and structures of polymer backbones and side chains.

The addition of silicon element into the polymer matrix in the past has been widely acknowledged for its flame-retardant capabilities by leaving inorganic silica residues upon being exposed to elevated temperatures. It acted as a barrier to mass transport, delaying the volatilisation of decomposition products. As a result, the number of volatiles available for combustion in the gas phase is reduced, which in turn decreases the heat feedback to the polymer surface. In addition, the silica residue insulates the underlying polymer from external heat flux [8,9]. More recent studies show that the introduction of silyl ether side chains containing bioactive substituents into the polymer backbone enables the development of pH-dependent drug delivery agents that undergo hydrolysis within the organism [10]. Additionally, by adjusting the substituents and the longevity of the potential alkyl-substituents, the hydrolysis time can be fine-tuned, allowing targeted drug delivery to specific locations [11]. Although conventional polynorbornene and its materials, such as brush-arm star polymers (BASPs) prepared from ROMP of norbornene terminated with polyethylene glycol (PEG) were once considered highly non-biodegradable [12], recent reports indicate efforts to modify their characteristics for potential applications in triggered self-assembly, stimuli-responsive materials, and biomedicine. These modifications aim to enhance their sustainability. One effective approach involves incorporating silyl ether-based cyclic olefins as copolymers, leading to polymer backbone degradability under mild acidic aqueous conditions [13]. Silicon materials are known for their bio-inertness and biocompatibility, and in the future, biotemplated self-assembled Si micro- and nanocomposites could potentially replace chemically synthesised silicates and serve as suitable materials for scaffold development in hard tissue engineering [14]. By introduction of silicon compounds, particularly silyl ethers, into the polynorbornene structure, the toxicity of such materials is expected to decrease, resulting in enhanced biodegradability. Currently, there is a growing demand in the modern petroleum industry to find suitable membrane-type materials for gas separation processes, making them highly promising options in the market. However, these materials face significant challenges in terms of selective permeability during long-term operation and the production process, which should be inexpensive, facile and environmentally friendly. To address these issues, recent studies propose the use of silyl-based poly(NBE) as glassy materials capable of selective gas absorption processes. These polymers are known for being highly permeable and demonstrate solubility-controlled permeation of hydrocarbons [15], attributed to their microporous structure [16,17,18] and presence of large free volumes [17,19]. Remarkably, polynorbornenes bearing side bulky substituents Me_3_Si- exhibit exceptional membrane properties due to the mentioned characteristics, resulting in extremely high gas permeability coefficients [20,21,22,23]. Another group of polymers that also show similar properties are polynorbornenes with Si-O-Si [24,25] or Si-O-C [26,27] groups attached. While they are not considered as microporous or highly permeable polymers with high free volume, they still demonstrate solubility-controlled permeation of hydrocarbons. These polymers possess mechanical and thermal properties resembling those of glassy polymers, but their membrane characteristics are closer to those of rubbers. Furthermore, their permeability coefficients increase for molecules of larger sizes. 

**Scheme 2:**
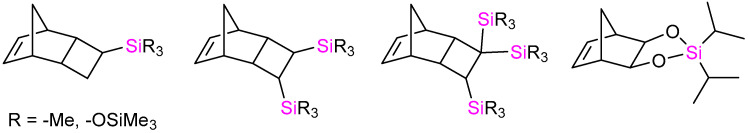
Examples of reported norbornene-based silicon compounds.

So far, based on our knowledge, there have been no reports on the synthesis of 5-norbornene-2-*exo*-3-*exo*-based linear polymeric materials bearing silyl ethers. The only examples available are from the aforementioned gas separation studies (without an oxygen linker). Additionally, there is only one example of a similar molecule with a Si-O bond connected directly to the 2-*exo*-3-*exo* position of the norbornene ring; however, it lacks two of the –CH_2_—groups that normally increase the distance between the polymeric backbone and functional groups, making the mers less rigid [28] (Scheme 2).

**Scheme 3:**
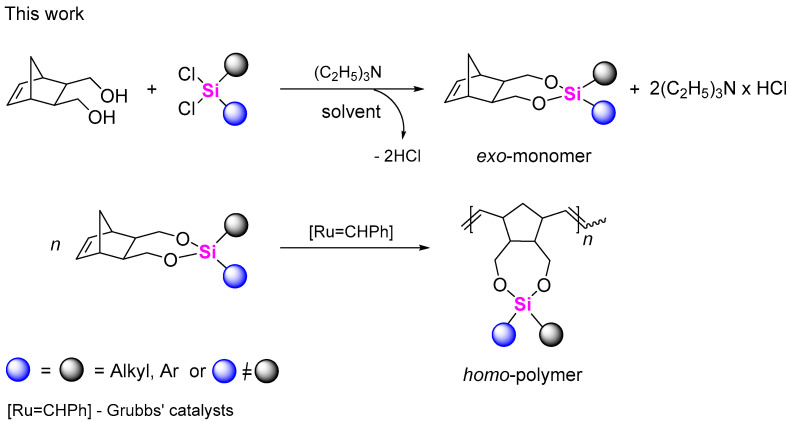
General overview of the research.

The purpose of this research is to present an efficient method for synthesis of new poly(norbornenes) with different silyl ethers attached through the condensation process of diol and mono-/dichlorosilanes and subsequent ring-opening metathesis polymerisation with high yields of acquired products (Scheme 3).

In future these materials may presumably find their applications in biomedicine, petroleum industries or as flame-retardant materials, upon further modifications.

## 2. Materials and Methods

### 2.1. Materials

The chemicals were obtained from the following sources: toluene, methylene chloride, n-hexane, tetrahydrofuran were purchased from Chempur (Piekary Śląskie, Poland) and P.O.Ch. (Gliwice, Poland). 5-norbornene-2-*exo*,3-*exo*-dimethanol (97%), dichlorodiphenylsilane (98%), dichlorodimethylsilane (99%), dichloromethylsilane (97%), chlorotrimethylsilane (99%), *tert*-butyl(chloro)diphenylsilane (98%) were acquired from Aldrich (Poznań, Poland), dichloro(methyl)phenylsilane (98%) and dichlorodiethylsilane (97%) from Thermo Scientific (Warsaw, Poland), chlorotriethylsilane (98%), dichlorodiisopropylsilane (98%) and 1,2-Bis(chlorodimethylsilyl)ethane (97%) from Angene (Hyderabad, Telangana), deuterated chloroform (CDCl_3_), Grubbs catalyst 1st generation C_43_H_72_Cl_2_P_2_Ru from Aldrich, ethyl vinyl ether from Merck (Warsaw, Poland). Acquired solvents toluene, DCM, THF and n-hexane were stored over molecular sieves type A4. Methylene chloride used in polymerisation was kept in Schlenk’s system in inert atmosphere over molecular sieves type A4. Please refer to the Appendix A for precautions.

### 2.2. General Procedure for Monomer (M1–M10) Synthesis

In a two-neck round-bottom flask filled with argon 5-norbornene-2-*exo*-3-*exo*-dimethanol was placed and solvent (toluene/THF) was added. The flask was then cooled to 0 °C in an ice bath, after which triethylamine was introduced in excess, followed by chloro-/dichlorosilane and mixed for the required time from 0 °C to room temperature. The progress of the reaction was observed via FTIR and GC-MS. After the completion of the reaction the mixture was filtered through a glass fibre syringe filter and the filtrate was concentrated under vacuum.

### 2.3. General Procedure for Polymer (P1–P8) Synthesis

A two-neck glass reactor under argon equipped with a magnetic stirring bar was loaded with monomer, followed by addition of methylene chloride (DCM) and Grubbs catalyst [Ru=CHPh]. The solution was stirred for the required time at room temperature, after which an excess (≈150 μL) of ethyl vinyl ether was added in order to terminate the reaction. The obtained mixture was then concentrated and polymeric material was precipitated by adding the rest of the solution dropwise to n-hexane. In the next step, the hexane was decanted and the polymer was dissolved once again in DCM and then precipitated. This step was repeated to remove any residual catalyst that may have remained in the mixture. After final decantation, polymer was dried under vacuum.

## 3. Results and Discussion

### 3.1. Synthesis of Silyl Ether Monomers

**Scheme 4:**
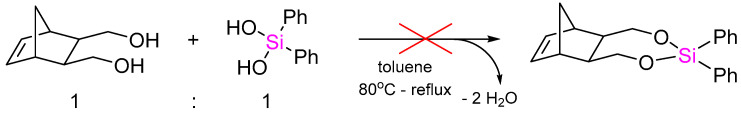
Diphenylsilanodiol condensation experiments.

Based on our previous studies concerning the synthesis and polymerisation of norbornene-based boronic esters [29,30], the preliminary studies focused on utilisation of diols and silanodiols in condensation reactions under similar reaction conditions. However, based on GC-MS the reaction did not proceed either in elevated temperature (80 °C) and in reflux with Dean–Stark apparatus attached (Scheme 4).

The lack of reaction progress was resolved by changing the type of substrate from silyl diols to dichlorosilanes. This substitution enabled the rapid synthesis of several new exo-monomers (M1–M6) with good isolation yields in the range of 60–90% (see Table 1). Furthermore, the optimised synthesis route allowed for control of the process, in which only trace amounts of byproducts were observed alongside the desired products. It is also worth noting that the addition of triethylamine (C_2_H_5_)_3_N in excess (≈2.1–3.1 eq.) allowed for facile separation of solvent from the forming triethylamine hydrochloride salt (C_2_H_5_)_3_N·HCl, thus providing an effective method for quick isolation without the need of using a higher amount of solvent in additional isolation steps (e.g., extraction). When it comes to the solvent used, THF proved to be the most efficient, as it allowed for complete precipitation of the salt. Most of the resulting monomers M2, M4, M5, M6 have formed dense-type colourless or yellow liquid products; only in two cases were the resulting products white solids M1 and M3 substituents which were easily soluble in organic polar solvents (DCM, CHCl_3_, THF) and a little less in the non-polar one (toluene) (Table 1).

**Scheme 5:**
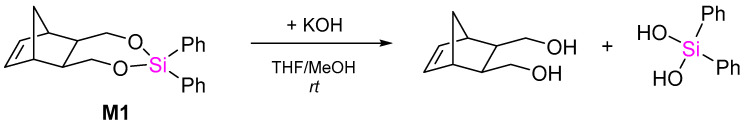
Silyl ether hydrolysis reaction.

According to the literature [13], silyl ether compounds exhibit decomposition upon hydrolysis either in acidic or basic conditions. The hydrolysis process on M1 was also examined and through the analysis of GC-MS it was confirmed that also norbornene-based silyl ether compounds undergo decomposition in those environments (Scheme 5).

**Figure 1:**
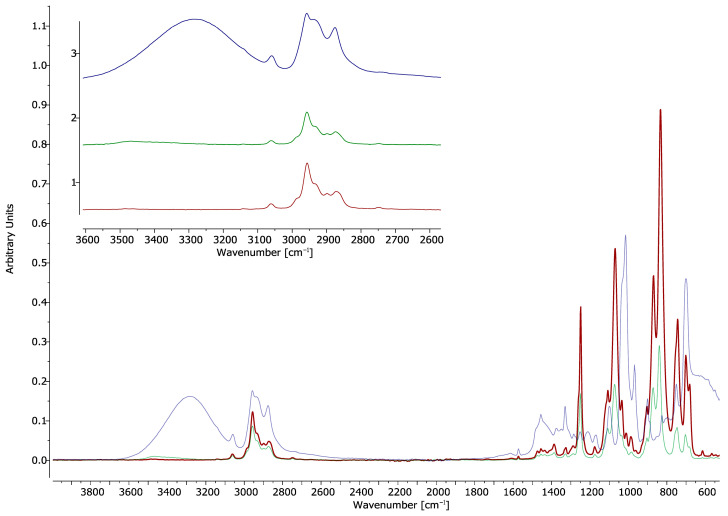
FT-IR spectra of reaction progress-synthesis of compound M7 (brown line), M7 mixture before adding Cl-SiMe_3_ in excess (green line) and NBE (CH_2_OH)_2_ (blue line) were measured at 25 °C as a solid state.

**Scheme 6:**
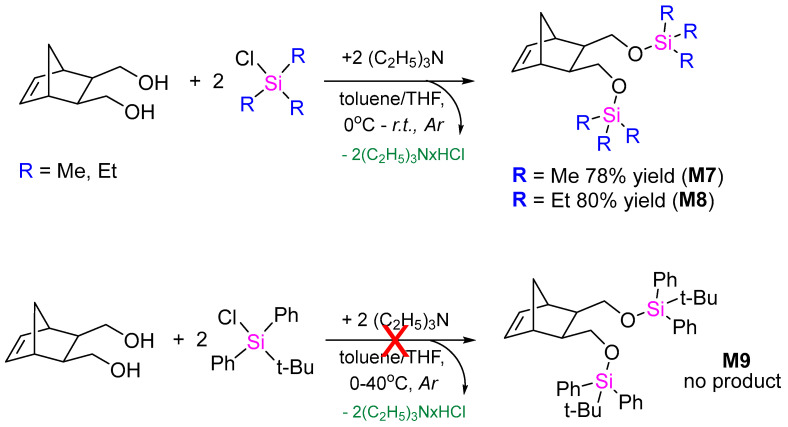
Condensation reaction of monochlorosilanes and norbornene diol.

A similar experiment was performed with the polymer, yielding a comparable hydrolysis reaction. Similarly to the M1 monomer, the formation of hydroxyl groups on the norbornene matrix was observed. After we had successfully made cyclic silyl ethers, we used a similar way of making chemicals to also get new open exo-,exo-bis(silyl)monomers using monochlorosilanes. Two further monomers M7 and M8 were synthesised in this manner and prepared for polymerisation. In both cases, condensation required excess of monochlorosilane (≈40% for M7 and 55% for M8) in order to acquire disubstituted monomer, as the GC-MS and FT-IR analyses performed upon the reaction in 1:2 molar ratio [diol]:[chlorosilane] showed also mono-substituted form (Figure 1). In order to see the limits of this approach, additional reaction was conducted on sterically hindered monochlorosilane containing tert-butyl and diphenyl substituents (M9) (Scheme 6). In this case no reaction was apparent by GC-MS spectra.

**Scheme 7:**
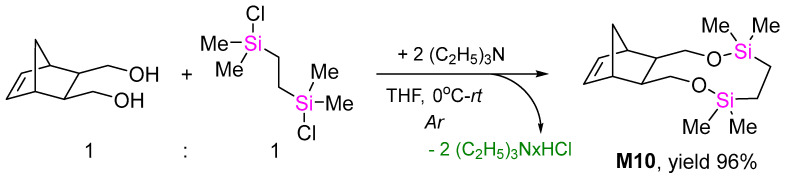
Condensation reaction of dichlorocarbosilane with norbornene diol.

We observed the disappearance of wide bands in the range of 3090 cm^−1^ to 3625 cm^−1^ affiliated to the hydroxyl groups of one substrate—norbornene diol (blue and brown spectra). In addition, an intense signal from the oxygen–carbon bond was visible at 1075 cm^−1^ (ether part, stretching), which can be attributed to the -H_2_C-O-Si- bonds system. Last but not least, by combining these two types of compounds it was possible to synthesise the cyclic bis-silyl ether monomer (M10) (Scheme 7):

While GC-MS in most cases provided certain information on the creation of desired products, the NMR studies gave a bit of insight on what happened during silyl ether formation. In most examples presented herein it could be seen that both monomers and further on polymers showed the occurrence of isomeric forms, which resulted in multiplication of certain peaks on the ^1^H NMR spectra and provided additional peaks on the ^13^C NMR and ^29^Si NMR. This phenomenon made it difficult to thoroughly analyse the synthesised products. Nevertheless, it was possible to distinguish the conversion of the monomer into the polymer, which represented the most important issue of this work.

### 3.2. Ring-Opening Metathesis Polymerisation

**Figure 2:**
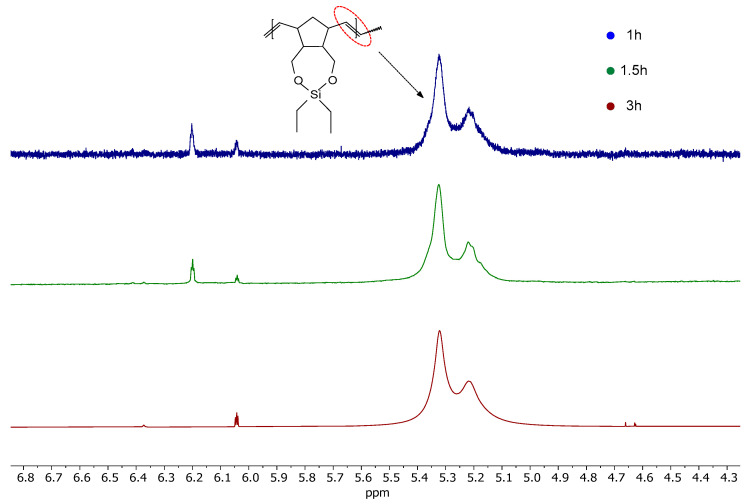
Polymerisation progress as seen on ^1^H NMR of P4 at 25 °C in CDCl_3_.

While the general monomer synthesis method proved to be quite efficient, the ring-opening metathesis polymerisation of obtained materials (P1–P6) demanded specific conditions in some cases. Previous works concentrated on ROMP of boronic esters [29,30] showed that the polymerisation process occurred rapidly, with full conversion, even after 5 min upon introduction of the ruthenium catalyst. Here, however, it was noticed that the reactions usually require longer propagation time (from 1 to 3 h) to observe the complete disappearance of the vinylene peak on ^1^H NMR spectra (Figure 2).

**Figure 3:**
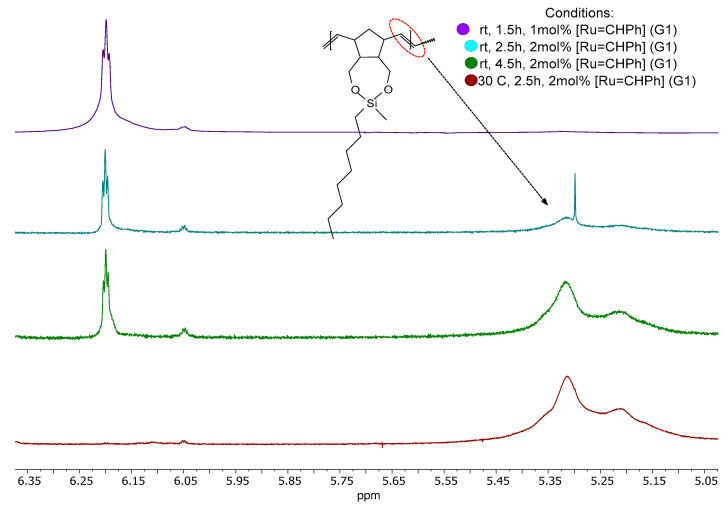
Polymerisation progress as seen on ^1^H NMR (P6) at 25 °C in CDCl_3_.

In each case, the progress of the reaction was monitored via ^1^H NMR. The conversion of the monomer was seen by the fading of the ‘sharp’ olefinic resonances of the vinylene groups inside norbornene units (≈6.2–6.3 ppm) while ‘broad’ polymer olefinic resonances (≈5.1–5.4 ppm) increased in intensity. Longer polymerisation time did not suffice for P6 as high residues of monomer were still present in the final product. It was necessary to provide additional optimisation of reaction conditions by introduction of changes in time, catalyst ratio and temperature. Full analysis of the proton spectrum confirmed the formation of a polymer consisting predominantly of *trans*-vinylene (63%) and lower amount of *cis*-vinylene (37%) fragments in the linear backbone. Eventually, polymerisation of M6 required slightly elevated temperature (30 °C) along with different catalyst ratio (2 mol%) (Figure 3).

The isolation was performed through the precipitation in non-polar solvent (n-hexane). Most of the polymers were received in solid state, giving white-beige solids, except P6, which formed a lightly brown membrane. P1, P3, P4, P5 were soluble in polar solvents, while P6 was easily soluble in both polar and non-polar solvents, probably due to the presence of a long, linear hydrocarbon substituent. Curiously, when it comes to the P2, ^1^H NMR analysis did not show any signs of polymerisation (Table 2). Its more complex studies are presented below.

Regarding the P2, tests P2a–P2c have been performed to assess the possibility of finding slight traces of polymerisation (Table 3). During the P2a test slight opacity was visible upon inserting the catalyst. After the termination of the reaction the mixture turned transparent; however, the precipitation process was not quite successful—only a very small portion of product precipitated, while the resulting mixture (n-hexane) turned slightly yellow. The ^1^H NMR studies showed that both the precipitate and the decanted fraction were not the expected products. It can be stated that the spectra showed no signs of conversion. The second test P2b was conducted at the slightly higher temperature of 30 °C. Here, however, a small portion of solid precipitated during the reaction while the resulting decant proved to be the monomer (^1^H NMR). The precipitated part was not soluble. To further investigate the problem the last test P2c was performed by extending the time of polymerisation under the same conditions. The prolongation of polymerisation resulted in precipitation of mostly insoluble solid. Analyses have been performed on a soluble fraction of the mixture that showed signs of decomposition of silyl ether as only methyl-based groups were observed. It is possible that further studies are required under different conditions, perhaps with use of another generation or type of catalyst.

**Scheme 8:**
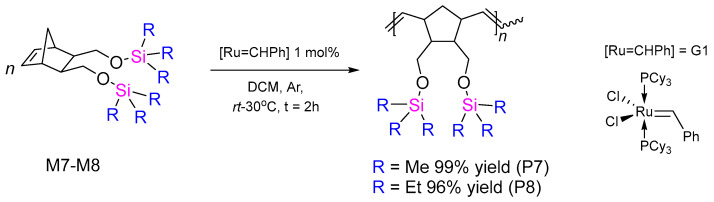
Polymerisations of bis(silyl)-acyclic monomer.

Ring-opening metathesis polymerisation of M7 and M8 also provided polymeric materials P7 and P8 (Scheme 8). Here however, the resulting polymers formed a brown membrane at the bottom of the reactor that was easily soluble in both polar and non-polar solvents, similarly to P6, due to the presence of hydrocarbons as substituents. That is why it was impossible to precipitate the formed polymers and receive solid state materials, and simultaneously the isolated yields reached approximately 100%. To achieve full conversion of the monomer P7 required elevated temperature as well (30 °C).

Considering monomer M10 a few different examinations have been performed P10a–d. The following polymerization reaction conditions were used: inert gas atmosphere Ar; room temperature and 30 °C; molar ratios [Mon.]:[Cat.] = 50:1, 100:1; reaction time: (a) 20, (b) 45, (c) 75, (d) 90 min. However, in this particular case, for the M10 monomer, only a brownish-beige, insoluble precipitate was formed after precipitation in n-hexane. P10a–P10c resulted in the formation of a black solid in the bottom of the vial that for the most part was insoluble. In contrast, for P10d the soluble fraction was not precipitated but only concentrated, resulting in a brownish membrane in the reactor. The NMR studies on small soluble fraction residues (P10a, P10d) of the material showed decomposition of the product rather than conversion of the monomer as the vinylene groups of the monomer were barely visible, but at the same time neither protons nor peaks normally attributed to the norbornene part of the compound in the elongating chains were spotted. These results show that further investigation is required to profoundly analyse the problem and find additional conditions and solutions to that problem.

### 3.3. GPC Analysis

Gel permeation chromatography measurements in THF provided good molecular weight values of the polymers P1: *M_n_* = 57,266 g/mol, *M_w_* = 116,420 g/mol, *Đ_M_* = 2.03. The second polymer P7 showed the following results: *M_n_* = 39,964 g/mol, *M_w_* = 45,311 g/mol, *Đ_M_* = 1.13. The third one P8 showed: *M_n_* = 36,036 g/mol, *M_w_* = 41,108 g/mol, *Đ_M_* = 1.14. It can be observed that linear-type polymers (P7–P8) showed much lower polydispersity index (PDI) in comparison to the cyclic one, P1. While P7 required higher temperature (30 °C) for full conversion, both of them have been polymerised for 2 h, which is one hour less than the P1, which theoretically should render higher *Đ_M_* (PDI = *M_w_/M_n_*), but that is not the case here. It seems that the presence of a sterically hindered enclosed single silyl ether group has an impact on the packing of polymer chains.

The above-described data is also shown in Table 4. Mass parameter analysis was performed only for good and highly soluble polymeric materials. For the figures regarding the GPC studies, see in the ESI.

### 3.4. TGA Analysis

The assessment of thermal stability of synthesised polymers showed interesting results concerning certain polymers. Highest weight losses for polymers P1, P2, P3, P4, P5 and P7 have been observed in the range of 300–525 °C (68–85% weight loss). Additionally, polymers P1 and P3, P5 bearing -SiPh_2_, -SiPhMe and -Si(i-Pr)_2_ functional groups showed very similar results with 5% weight loss ranging from 277 to 300 °C and 10% weight loss at 388–400 °C. P4 contains a fragment with a diethylsilyl group (-SiEt_2_) in its structure and is characterised by the lowest temperature values (155 °C and 221 °C) for 5 and 10% mass loss. However, the maximum mass loss was determined to be in the same range (303–504 °C) as for the other polymeric materials. The largest difference was observed for polymer P6, however. Its thermal stability was lower than that of the other compounds. Decomposition began gradually at 104 °C and progressed up to 329 °C in the first stage, resulting in a 40% mass reduction. The second stage of thermal decomposition ranged from 329 °C to 525 °C, resulting in a 48.3% mass loss.

Polymer P7, although containing two –OSiEt_3_ groups attached to the norbornene core, showed similar decomposition parameters as the rest of the polymers. For more information, TGA data results are presented in Table 5, and the ESI. To the best of our knowledge, there are no other similar linear materials with a mono- or disilyl ether structure documented in the existing literature. However, in comparison to cross-linked products comprising siloxane fragments, the stable ‘*plato*’ temperature range is lower, ranging from 78 °C to 194 °C. The exception to this is P4, for which the ‘*plato*’ ranges from 31 °C to 171 °C (maximum 2.5% sample weight loss). However, for Td5 and Td10, in a manner analogous to the final stages of decomposition of our homopolymers, temperatures are higher between 20 °C and 110 °C. The total mass loss of material P7 starts at 745–750 °C, and the products mentioned above completely decompose around 500 °C [31,32]. The process of thermal decomposition of organic compounds in the absence of oxygen is a complex process which can occur in multiple directions, leading to the formation of a variety of pyrolysates, including gases, semivolatile substances, and carbons. It is imperative to note that the TGA analysis process involves the decomposition of a polymeric material in an atmosphere of dry, inert nitrogen (pyrolysis). Consequently, the oxidation step of the double bond in the polynorbornene matrix should be omitted. In order to establish a probable decomposition mechanism, consideration was given to the bond energies of organosilicon and organic compounds that bear fragmentary similarity to the compounds under consideration. The single bond energies of silicon ethers (Si-O) range from 450 to 452 kJ/mol, those of silicon-carbon (Si-C) from 318 to 327 kJ/mol, and those of organic compounds (O-C) from 350 to 358 kJ/mol. The bond energies of carbon (single C-C) range from 350 to 410 kJ/mol, those of double C=C from 610 to 630 kJ/mol, and those of the C-H bond from 413 kJ/mol [33]. A comprehensive analysis of the numerical energy values indicates that the structural decomposition can be executed in the following sequence: firstly, concurrent cleavage of individual C-C and ether -O-C- carbon bonds, followed by fragmentation of the polymer matrix and silicon organic substituents. However, it has been demonstrated that the same type of matrix carbon bonds are also cleaved. Subsequently, thermal cleavage of the silicon–carbon bond, originating from the Si-organic substituents, fragments and of the norbornene (NB) itself, occurs. The decomposition process continues with the loss of a hydrogen atom from the organic substituents, followed by the separation of some of the norbornene at higher temperatures. This process leads to the formation of silicon and various hydrocarbons, including methane and acetylene. The decomposition mechanism of the silicon ether fragment, the -C-O-Si- bond, must be considered separately. It is hypothesised that this process will result in dehydration and condensation, leading to the formation of silicon anhydrides and siloxanes, in addition to the formation of a protective char layer. The process of forming a char is characterised by the cleavage of the silicon–oxygen–carbon (Si-O-C) bonds, resulting in the formation of silicon oxide (SiO_2_).

### 3.5. SEM Analysis

**Figure 4:**
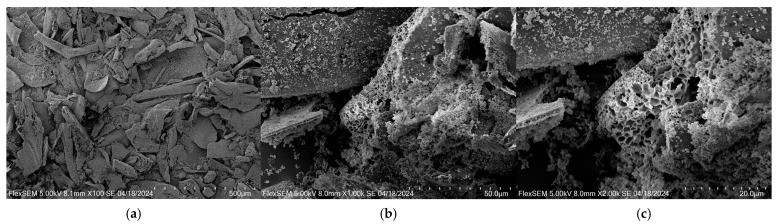
(**a**) SEM image of the surface of P1 (powder) in a 100× magnification; (**b**) SEM image of the surface of P1 (powder) in a 1000× magnification; (**c**) SEM image of the surface of P1 (powder) in a 2000× magnification.

In order to further investigate the physical properties, additional surface studies of the polymeric materials were performed using scanning electron microscopy (SEM). The polymers were examined in powder form and as films (preparation: the material was dissolved in DCM and allowed to slowly evaporate at room temperature). The powder surfaces demonstrate substantial porosity. The general structure for P1 is illustrated in Figure 4a–c (with =SiPh_2_ part). A cluster of porous structures with micropores—that is to say, spatial voids—can be observed across the entire surface.

**Figure 5:**
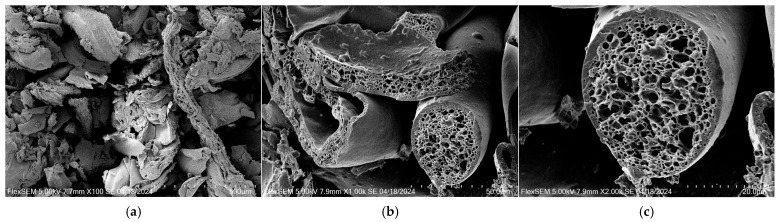
(**a**) SEM image of the surface of P3 (powder) in a 100× magnification; (**b**) SEM image of the surface of P3 (powder) in a 1000× magnification; (**c**) SEM image of the surface of P3 (powder) in a 2000× magnification.

The magnification of a cross-section of one such irregular plate showed the existence of pores underneath the smooth surface. It can be seen also that the cavities (pores) occur as a layer between the smooth surfaces of material. It can be observed on the 4b and 4c panels that one of the plates is broken and rotated almost 90° in the mid-left of the photo. It can also be seen that there are white crystallites on the smooth surfaces of the material. We were particularly intrigued by the SEM images of the P3 polymer, Figure 5a–c. The general structure of P3 (with =SiMePh part) is characterised by significant irregularity and fragmentation. It is possible to discern both plates and intertwined fibres.

However, analysis of the cross-sections of these structures indicates a high degree of porosity. Additionally, the presence of blisters on the lateral surfaces of the structures has been observed; see Figure 5b. As illustrated in the third image of polymer P3 (see Figure 5c), an isolated pore structure resembling a human bone in cross-section is clearly visible. The irregular pores are enclosed in single layers or oval tubes (see Figure 5b,c). The polymeric material films exhibited a high degree of homogeneity, with traces of contamination originating from beneath the film layers. Furthermore, minor fissures are also discernible. Nevertheless, the films are characterised by uniformity and smooth surfaces. The photographs do not reveal the presence of characteristic pores; however, the detection of small particles is possible. Representative images for a selection of polymers are appended in the ESI.

## 4. Conclusions

A series of norbornenyl silyl ethers has been prepared for the first time. Following the optimisation of the reaction conditions, it was found that the majority of the prepared monomers were susceptible to the ring-opening metathesis polymerisation (ROMP) process. This enabled the synthesis of both monomers and polymers with high isolation yields, indicating the efficiency and relative simplicity of the overall procedure. However, it should be noted that norbornene derivatives bearing silyl ether functionalities generally require a broader range of reaction conditions compared to other norbornene-based systems, such as previously described boronic esters [29,30]. It was established that a proportion of the materials obtained contained sterically hindered substituents, which consequently resulted in the formation of insoluble polymers. Consequently, it was not possible to determine the polydispersity (*Đ_M_*) for all materials. Notwithstanding, the polymers demonstrated heightened thermal stability and a substantial degree of porosity, as substantiated by SEM imaging. These properties may, in the future, enable the development of materials designed to operate at elevated temperatures or for applications in gas separation processes. Despite the fact that polymeric materials were not successfully obtained in all cases, further studies will be undertaken to investigate and address these limitations.

## Figures and Tables

**Table 1:** Condensation reaction of diol and mono-/dichlorosilanes.

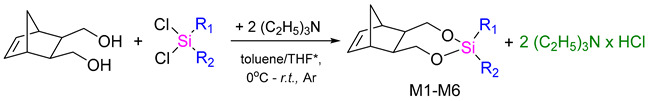					
**Entry**	**R_1_**	**R_2_**	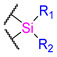	**Time** **[h]**	**Isolated Yield** **[%]**
M1	Ph	Ph	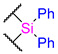	1.5	90
M2	Me	Me	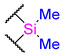	3	60
M3	Ph	Me	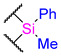	1.5	90
M4	Et	Et	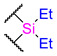	1.5	81
M5	i-Pr	i-Pr	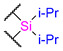	1.5	73
M6	Me	Oc	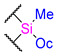	1	78

* Toluene and THF were dried and deoxygenated under argon.

**Table 2:** Ring-opening metathesis polymerisation of cyclic silyl ether monomers.

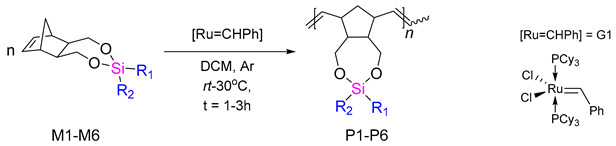							
**Entry**	**R_1_**	**R_2_**	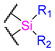	**Temperature** **[°C] ^(a)^**	**Molar Ratio** **Cat./Mon**	**Time** **[h]**	**Isolated** **Yield (%)**
P1	Ph	Ph	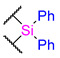	rt	1:100	3	97
P2	Me	Me	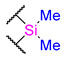	---	---	---	---
P3	Ph	Me	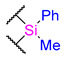	rt	1:100	1.5	93
P4	Et	Et	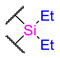	rt	1:100	1.5	85
P5	i-Pr	i-Pr	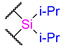	rt	1:100	1 h	79
P6	Me	Oct	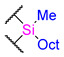	30	1:50	2	98

^(a)^ rt—23–25 °C; the solvent DCM was dried and deoxygenated under Ar; --- No reaction product.

**Table 3:** ROMP conditions of polymers P2.

Entry	Temperature [°C]	Molar Ratio Cat./Mon	Time [min]
P2a	rt ^(a)^	1:100	30
P2b	30	1:100	25
P2c	30	1:100	120

^(a)^ rt.—23–25 °C; the solvent DCM was dried and deoxygenated under argon; inert gas Ar.

**Table 4:** GPC results.

Polymer	*M_n_* ×(10^5^)	*M_w_* ×(10^5^)	*Đ_M_* (*M_w_*/*M_n_*)
P1	0.57	1.16	2.03
P7	0.40	0.45	1.13
P8	0.36	0.41	1.14

Analysis conditions: rt—23–25 °C; solvent THF was degassed before use; *Đ_M_*—PDI = *M_w_*/*M_n_.*

**Table 5:** TGA analysis results of polymeric materials.

Entry	T_d_^5^ (°C) ^(a)^	T_d_^10^ (°C) ^(a)^	1st Step of Thermal Decomposition (Weight Loss (%)) Top Temp. ^(a)^ (°C)	2nd Step of Thermal Decomposition (Weight Loss (%)) Top Temp. ^(a)^ (°C)	3rd Step of Thermal Decomposition (Weight Loss (%)) Top Temp. ^(a)^ (°C)	4th Step of Thermal Decomposition (Weight Loss (%)) Top Temp. ^(a)^ (°C)
P1	298.8	387.7	192.4–324.8 (4.7) Max. 256.1	324.8–536.0 (71.9) Max. 454.6	536.0–746.4 (19.9) Max. 568.7	----
P3	277.1	387.8	193.7–326.2 (5.2) Max. 251.4	326.2–539.3 (75.6) Max. 460.3	----	----
P4	155.3	221.1	76.7–172.9 (5.7) Max. 136.1	172.9–303.3 (9.9) Max. 234.5	303.3–504.4 (68.0) Max. 441.9	504.4–622.0 (11.2) Max. 535.7
P5	299.5	400.8	109.7–221.3 (2.6) Max. 167.1	221.3–326.2 (3.0) Max. 283.1	326.2–525.9 (84.8) Max. 453.5	----
P6	203.5	222.5	104.3–328.9 (40.1) Max. 246.5	328.9–525.2 (48.3) Max. 442.3	525.2–749.8 (4.7) Max. 661.2	----
P7	231.1	287.4	171.5–261.0 (5.1) Max. 232.5	261.0–346.4 (8.0) Max. 293.8	346.4–512.4 (80.2) Max. 440.4	512.4–732.3 (3.0) Max. 580.7

^(a)^ Determined by TGA analysis: N_2_, 10 °C/min; 25–1000 °C; total weight residue (%): P1 (3.6), P3 (7.8), P4 (5.0), P5 (5.4), P6 (6.6), P7 (1.6). Each measurement was performed twice.

## Data Availability

The original contributions presented in this study are included in the article/Supplementary Material. Further inquiries can be directed to the corresponding author.

## Supplementary Materials

The following supporting information can be downloaded at: https://www.mdpi.com/article/10.3390/ma19091681/s1, ESI consists of: NMR Spectra (^1^H NMR, ^13^C NMR, ^29^Si NMR) of monomers M1–M8, M10 as: Figures S1–S27, polymers P1–P8 as: Figures S28–S48; GPC analysis for polymers P1, P7, P8 as: Figures S49–S51; TGA curves for polymers P1, P3–P7 as: Figures S52–S57; SEM images as: Figures S58–S62; LIBS/ICP images as: Figures S63–S65. All ESI data is collected in PDF file. Ref. [34] is cited in the Supplementary Materials.

## Abbreviations

ROMP	Ring-opening metathesis polymerisation
NBE	Norbornene
NMR	Nuclear magnetic resonance
GC-MS	Gas chromatography–mass spectrometry
ĐM	polydispersity index
DCM	Dichloromethane
Oct	Octyl group (C_8_H_17_)
Ph	Phenyl group (C_5_H_6_)
*i*-Pr	isopropyl group (C_3_H_7_)
Et	Ethyl group (C_2_H_5_)
SEM	Scanning electron microscope
TGA	Thermogravimetric analysis
PCy_3_	Tricyclohexylphosphine

## Appendix A

It is imperative that these compounds are handled with the utmost care and in accordance with stringent safety protocols. This involves the utilisation of proper needle and syringe techniques, along with the employment of a vacuum Schlenk line. It is imperative to note that contact between the substance and the skin or the respiratory system must be strictly avoided. All manipulations with these compounds were performed in strict accordance with the established safety procedures [35].
