# Supplementary material for: Design, Synthesis and ROMP of Novel Exo-Norbornene Silyl Ethers for Functional Polymer Applications
Source: Materials (Basel). 2026 Apr 22;19(9):1681. doi: 10.3390/ma19091681 (PMC13165061; doi:10.3390/ma19091681)
Supplement: Supplementary file 1 [file materials-19-01681-s001.zip › materials-4236761-supplementary.pdf]

Electronic Supplementary Information

Design, Synthesis and ROMP of Novel *Exo*-Norbornene Silyl Ethers for Functional Polymer Applications

Mariusz Majchrzak[1*], Jerzy Garbarek[1] and Ahmed M. Eissa[2,3,4]

[1] Department of Organometallic Chemistry, Faculty of Chemistry, Adam Mickiewicz University in Poznań, 8 Uniwersytetu Poznańskiego Str., 61-614 Poznań, Poland; jerzy.garbarek@amu.edu.pl

[2] Research Institute of Healthcare Sciences, School of Pharmacy & Life Sciences, Faculty of Science and Engineering, University of Wolverhampton, Wolverhampton WV1 1LY, UK; a.m.eissa@wlv.ac.uk

[3] Department of Chemistry, School of Engineering, University of Warwick, Coventry CV4 7AL, UK

[4] Department of Polymers, Chemical Industries Research Division, National Research Centre, El-Bohouth St. 33, Dokki, Giza 12622, Cairo, Egypt

* Correspondence: mariusz.majchrzak@amu.edu.pl; Tel.: +48-(61)-8291847

Table of contents

Experimental 2

Techniques / Methods 2

Monomer synthesis 3

Polymer synthesis 7

NMR Spectra ^{1}H, ^{13}C, ^{29}Si NMR 9

Monomers 9

Polymers 27

GPC Analysis 41

TGA analysis (thermogravimetric curves) 43

SEM Images 46

LIBS/ICP analysis and images 46

Referencens 46

Experimental

Techniques

NMR measurement: ^{1}H NMR (400 MHz), ^{13}C NMR (105 MHz) and ^{29}Si NMR (99 MHz) spectra were recorded on a Mercury plus 400MHz spectrometer (Varian, Inc., Palo Alto, Ca, USA) and Bruker Avance 600 MHz (Bruker Corporation, Billerica, Ma, USA). In each method, $CDCl_3$ solution was used as residue solvent. Chemical shifts are reported in δ (ppm) with reference to the residue solvent used ($CDCl_3$) (^{1}H δ H = 7.26 ppm, ^{13}C δ C = 77.36 ppm).

GC-MS measurement: Mass spectra (GC-MS) of monomers were obtained on Varian 3300 instrument (Varian, Inc. 2700 Mitchell Drive Walnut Creek, Palo Alto, CA, USA) equipped with capillary column type DB-1 ResteckTM (Agilent Technologies/Glastron Inc., Vineland, NJ, USA), which was connected to Finnigan MAT 800 spectrometer (Thermo Fisher Scientific, Bremen, Germany) with ion trap detector. Spectra were registered by using ITD Finnigan MAT program.

HR-MS measurement: High-resolution mass spectroscopic (HRMS) analyses were performed on an AMD-402 mass spectrometer which is a two-sector (B/E geometry) mass spectrometer manufactured by AMD Intectra GmbH (Germany). Two measurements were made for each monomer.

Elemental Analysis measurement: An elemental analysis was performed on a Vario Elementar Analyser Vario EL III Instrument (German) three times and then averaged to obtain the final results. The temperature range of analysis was 25–1100°C. The method of analysis involves catalytic combustion of the sample at 1200°C with gas separation on adsorption columns. Detection of material components was identified by thermal conductivity difference. The duration of a single analysis varied from 10 to 20 minutes with simultaneous determination of the elements: C, H, N, S. Three E. A. measurements were made from three weighed portions of each polymer.

FT-IR measurement: Infrared spectroscopy (FT-IR) was performed on NICOLETTM iSTM50FT-IR spectrometer made by Thermo ScientificTM (Madison, WI, USA) with addition of iSTM50ATR, in the range between 4000 cm^{-1} and 400 cm^{-1}. For each spectrum 16 scans were registered.

GPC measurement: Gel permeation chromatography (GPC) analyses were performed using an Agilent 1260 Infinity system (Agilent Technologies, Waldbronn, Germany) equipped with a Phenogel 10 um Linear(2) 300 x 7.8 mm column and RI detector. The samples were dissolved in tetrahydrofuran (THF). Conditions of measurements: mobile phase tetrahydrofuran (THF); flow rate (THF) = 1 ml/min; column temperature = 35°C; RI detector temperature = 35°C; analysis time = 15 min. The molecular weights of the analyzed compounds and their mass scatterings were determined with reference to linear poly(styrene) standards with masses in the range of 1000-3500000. Data was processed using Agilent GPC/SEC software—1.2.3182.29519. The GPC measurements presented in this article were performed only on highly soluble materials.

SEM measurement: Scanning electron microscopy (SEM) was done on FlexSEM 1000 II apparatus (Hitachi, London/Manchester, UK) featuring electron optical and signal detection systems providing unparalleled imaging and analytical performance. Analysis parameters: accelerating voltage 5.00 kV (penetrating beam strength), spot intensity 20.00 and working

distance 5.00. The sample was put in high vacuum before purging with argon and then it was gold-coated by sputtering for 4 minutes (Gold coater: EMSCOPE).

TGA measurement: Thermal stability was investigated by thermogravimetric analyser -TGA Q50 V20.10 Build 36 (TA Instruments, New Castle, DE, USA). Samples of about 2.90 to 9.04 mg were heated in platinum pans at the rate of 10°C/min from 25°C to 900°C and 1000°C under nitrogen atmosphere (90 mL/min). Each measurement was performed twice.

LIBS measurement (laser-induced plasma (LIP)): Inductively Coupled Plasma was measured with a laser-based elemental analyzer, EA–300 (KEYENCE, Japan), which ensures a flexible switching between the LIBS head (EA–300) and high-performance microscope camera (with the VH–Z20 lens). The LIBS system is equipped with a nanosecond Nd:YAG laser with a wavelength of 355 nm, a fixed spot size (10 μm) and magnification (300x). The spectrometer software detects the emission lines of elements and reports their content automatically above the LOQ = 0.10% wt. (equal to LOD = 0.03% wt.). The display of the content of each element can be forced manually (as done in several examples).

Methods

Purification and drying of solvents: Purification, drying and deoxidation of solvents and reagents used in the work were carried out on the basis of standard methods described in the literature [1]. The solvents were stored in Schlenk vessels under an argon atmosphere and kept in the previously calcined molecular sieves A4.

Methodology of work in an inert gas atmosphere: Reactions in an inert gas atmosphere were carried out using a vacuum gas manifold (Schlenk line). The inert gas used was Class 5.0 argon. All solvents and liquid reagents used in the reactions were dehydrated and dried according to standard procedures described above.

Monomer synthesis

5-norbornene-2-exo,3-exo-dimethyl[2,2-diphenyl-1,3,2-dioxasilane] —**M1**— was obtained according to the preparation procedure mentioned in the article, using 0.15 g (0.97 mmol) of diol (p = 97%), 280 μL (0.2 g; 2 mmol) of triethylamine (p = 99%) and 0.244 g (0.199 mL; 0.96 mmol) of dichlorodiphenylsilane (p = 98%) in 5 mL of THF, mixed for 1.5 h, with 90% yield (0.293 g; 0.88 mmol) as white solid.

*Analytical data **M1***: ^{1}H NMR (400 MHz, $CDCl_3$): δ 7.76-7.29 (m, 10H, Ar-*H* (A)); 6.22 (t, 2H, -C*H*=C*H*- (B), J_{H-H}=1.6Hz); 4.14 (dd, 2H, -C*H*$_2$- (C), J_{H-H}=4.0, 12.0Hz); 3.96 (t, 2H, -C*H*$_2$- (D), J_{H-H}=11.6Hz); 2.48 (p, 2H, -C*H*- (E), J_{H-H}=2.0Hz); 2.17-2.09 (m, 2H, -C*H*- (F)); 1.32 (dt, 1H, -C*H*$_2$- (G), J_{H-H}=1.6, 8.8Hz); 1.23 (dt, 1H, -C*H*$_2$- (H), J_{H-H}=1.6, 8.8Hz). ^{13}C NMR (105 MHz, $CDCl_3$): δ 137.7; 135.3; 134.5; 130.6; 128.3; 128.0; 66.8; 45.4; 44.24; 44.16. ^{29}Si NMR (99 MHz, $CDCl_3$): δ -26.6. MS (EI) [m/z (relat. int. %)]: 333.8 ($M^{+\bullet}$) (4), 268.9 (100), 267.0 (53), 66.2 (45), 160.1 (31), 190.2 (30), 91.2 (20). MS (FAB, m/z (%) (M^+, 100): 334. HRMS (M/Z) calcd. for $C_{21}H_{22}O_2Si$: 334.1389; found 334.1387.

5-norbornene-2-exo,3-exo-dimethyl[2,2-dimethyl-1,3,2-dioxasilane] —**M2**— was obtained according to the preparation procedure mentioned in the article, using 0.15 g (0.97 mmol) of

diol (p = 97%), 280 μL (0.2 g; 2 mmol) of triethylamine (p = 99%) and 0.123 g (0.116 mL; 0.95 mmol) of dichlorodimethylsilane (p = 99%) in 5 mL of THF, mixed for 3 h, with 60% yield (0.123 g; 0.59 mmol) as colorless liquid.

*Analytical data **M2***: ^{1}H NMR (400 MHz, $CDCl_3$): δ 6.20 (t, 2H, -C*H*=C*H*- (A), J_{H-H}=2.0Hz); 3.93 (dd, 2H, -C*H*$_2$- (B), J_{H-H}=3.6, 12.0Hz); 3.83 (t, 2H, -C*H*$_2$- (C), J_{H-H}=11.2Hz); 3.77 (t, 2H, -C*H*$_2$- (C-2nd isomer), J_{H-H}=6.8Hz); 2.54 (p, 2H, -C*H*- (D- 2nd isomer), J_{H-H}=1.6Hz); 2.47 (p, 2H, -C*H*- (D), J_{H-H}=1.6Hz); 2.00-1.93 (m, 2H, -C*H*- (E)); 1.86-1.81 (m, 2H, -C*H*- (E- 2nd isomer)); 1.42(dt, 1H, -C*H*$_2$- (F), J_{H-H}=1.6, 8.8Hz); 1.27(dt, 1H, -C*H*$_2$- (G), J_{H-H}=1.6, 8.8Hz); 0.18(s, 3H, -CH_3 (H)); 0.14(s, 3H, -C*H*$_3$ (H)). ^{13}C NMR (105 MHz, $CDCl_3$): δ 137.7; 65.9; 45.4; 44.15; 44.09; -2.1. ^{29}Si NMR (99 MHz, $CDCl_3$): δ -10.5, -12.2, -19.6 (silicon grease), -29.7 (silicon grease). MS (EI) [m/z (relat. int. %)]: 210.9 ($M^{+\bullet}$) (4), 144.9 (100), 143.2 (88), 91.0 (35), 66.0 (63). HRMS (M/Z) calcd. for $C_{11}H_{18}O_2Si$: 210.1076; found 210.1075.

5-norbornene-2-exo,3-exo-dimethyl[2-methyl-2-phenyl-1,3,2-dioxasilane] —**M3**— was obtained according to the preparation procedure mentioned in the article, using 0.15 g (0.97 mmol) of diol (p = 97%), 280 μL (0.2 g; 2 mmol) of triethylamine (p = 99%) and 0.184 g (0.156 mL; 0.96 mmol) of dichloromethylphenylsilane (p = 98%) in 5 mL of THF, mixed for 1.5 h, with 90% yield (0.239 g; 0.88 mmol)) as white solid.

*Analytical data **M3***: ^{1}H NMR (400 MHz, $CDCl_3$): δ 7.74-7.27 (m, 5H, Ar-*H* (A)); 6.19 (t, 2H, -C*H*=C*H*- (B), J_{H-H}=2.4Hz); 3.76 (d, 2H, -C*H*$_2$- (C), J_{H-H}=7.6Hz); 3.64 (s, 2H, -C*H*$_2$- (D)); 2.53 (p, 2H, -C*H*- (E), J_{H-H}=2.4Hz); 1.88-1.80 (m, 2H, -C*H*- (F)); 1.36 (dt, 1H, -C*H*$_2$- (G), J_{H-H}=2.0, 12.0Hz); 1.26 (dt, 1H, -C*H*$_2$- (H), J_{H-H}=2.0, 12.0Hz); 0.56-0.15 (m, 3H, (I)). ^{13}C NMR (105 MHz, $CDCl_3$): δ 137.7; 137.6; 133.4; 130.3; 127.9; 65.1; 45.9; 44.0; 43.6; -2.3. ^{29}Si NMR (99 MHz, $CDCl_3$): -11.9. MS (EI) [m/z (relat. int. %)]: 273.0 ($M^{+\bullet}$) (16), 257.0 (26), 207.2 (100), 205.2 (49), 129.2 (28), 119.2 (97), 91.0 (40), 66.2 (23). MS (FAB, m/z (%) (M^+, 100): 273. HRMS (M/Z) calcd. for $C_{16}H_{20}O_2Si$: 272.1233; found 272.1232.

5-norbornene-2-exo,3-exo-dimethyl[2,2-ethyl-1,3,2-dioxasilane] —**M4**— was obtained according to the preparation procedure mentioned in the article, using 0.2 g (1.3 mmol) of diol (p = 97%), 372 μL (0.27 g; 2.67 mmol) of triethylamine (p = 99%) and 0.204 g (0.194 mL; 1.3 mmol) of dichlorodiethylsilane (p = 97%) in 6.7 mL of THF, mixed for 1.5 h, with 81% yield (0.25 g; 1.05 mmol) as colorless/slightly white oleic liquid.

*Analytical data **M4***: ^{1}H NMR (400 MHz, $CDCl_3$): δ 6.20 (t, 2H, -C*H*=C*H*- (A), J_{H-H}=2.0Hz); 3.95 (dd, 2H, -C*H*$_2$- (B), J_{H-H}=5.6, 16.0Hz); 3.85 (t, 2H, -C*H*$_2$- (C), J_{H-H}=16.0Hz); 2.46 (p, 2H, -C*H*- (D), J_{H-H}=2.0Hz); 2.00-1.91 (m, 2H, -C*H*- (E)); 1.40 (dt, 1H, -C*H*$_2$- (F), J_{H-H}=2.0, 11.6Hz); 1.26 (dt, 1H, -C*H*$_2$- (G), J_{H-H}=2.4, 11.6Hz); 1.01-0.91 (m, 6H, -C*H*$_3$ (H)); 0.72-0.47(m, 4H, -C*H*$_2$- (I)). ^{13}C NMR (105 MHz, $CDCl_3$): δ 137.7; 66.1; 45.4; 44.12; 44.09; 7.5; 6.8; 6.4; 6.1; 4.6; 2.9. ^{29}Si NMR (99 MHz, $CDCl_3$): -0.3; -9.2. MS (EI) [m/z (relat. int. %)]: 239.0 ($M^{+\bullet}$) (36), 209.0 (17), 173.1 (59), 119.0 (100), 117.3 (24), 91.0 (26). MS (FAB, m/z (%) (M^+, 100): 239. HRMS (M/Z) calcd. for $C_{13}H_{22}O_2Si$: 238.1389; found 238.1388.

5-norbornene-2-exo,3-exo-dimethyl[2,2-diisopropyl-1,3,2-dioxasilane] —**M5**— was obtained according to the preparation procedure mentioned in the article, using 0.1 g (0.65 mmol) of diol (p = 97%), 186 μL (0.14 g; 1.33 mmol) of triethylamine (p = 99%) and 0.12 g (0.117 mL; 0.65 mmol) of dichlorodipropylsilane (p = 97%) in 3.34 mL of THF, mixed for 1.5 h, with 73% yield (0.126 g; 0.47 mmol) as colorless oleic liquid.

*Analytical data **M5***: ^{1}H NMR (400 MHz, $CDCl_3$): δ 6.20 (t, 2H, -C*H*=C*H*- (A), J_{H-H}=1.6Hz); 3.98 (dd, 2H, -C*H*$_2$- (B), J_{H-H}=4.0, 11.6Hz); 3.91 (t, 2H, -C*H*$_2$- (C), J_{H-H}=11.2Hz), 2.45 (p, 2H, -C*H*- (D), J_{H-H}=2.0Hz); 1.97-1.90 (m, 2H, -C*H*- (E)); 1.37 (dt, 1H, -C*H*$_2$- (F), J_{H-H}=1.6, 8.8Hz); 1.24 (dt, 1H, -C*H*$_2$- (G), J_{H-H}=1.6, 8.8Hz); 1.10-0.83 (m, 14H, -CH-(CH_3)$_2$) (H,I); 0.06 (s, silicone grease). ^{13}C NMR (105 MHz, $CDCl_3$): δ 137.7; 66.7; 45.4; 44.2; 44.0; 17.4; 16.8; 12.3; 11.7. ^{29}Si NMR (99 MHz, $CDCl_3$): -5.5. MS (EI) [m/z (relat. int. %)]: 267.1 ($M^{+\bullet}$) (3), 224.5 (31), 223.5 (100), 157.1 (27), 127.3 (29), 119.2 (59), 115.2 (45), 91.0 (53). MS (FAB, m/z (%) (M^+, 100): 267. HRMS (M/Z) calcd. for $C_{15}H_{26}O_2Si$: 266.1702; found 266.1701.

5-norbornene-2-exo,3-exo-dimethyl[2-methyl-2-octyl-1,3,2-dioxasilane] —**M6**— was obtained according to the preparation procedure mentioned in the article, using 0.2 g (1.30 mmol) of diol (p = 97%), 372 μL (0.27 g; 2.67 mmol) of triethylamine (p = 99%) and 0.289 g (1.27 mmol) of dichloromethyloctylsilane (p = 99%) in 6.7 mL of THF, mixed for 1 h, with 78% yield (0.312 g; 1.01 mmol) as slightly white oleic liquid.

*Analytical data **M6***: ^{1}H NMR (400 MHz, $CDCl_3$): δ 6.20 (d, 2H, -C*H*=C*H*- (A), J_{H-H}=2.8Hz); 3.92-3.76 (m, 4H, -C*H*$_2$- (B)); 2.50 (dt, 2H, -C*H*- (C), J_{H-H}=2.0, 32.0Hz); 1.97-1.84 (m, 2H, -C*H*- (D)); 1.26 (s, 14H, -C*H*$_2$- (E)); 0.88-0.84 (m, 3H, -C*H*$_3$ (F)); 0.64 (dt, 2H, -C*H*$_2$- (G), J_{H-H}=9.2, 44.0Hz); 0.15-0.07(m, 3H, (H)). ^{13}C NMR (105 MHz, $CDCl_3$): δ 137.6; 65.1; 45.9; 44.0; 43.5; 33.3; 32.0; 29.5; 29.3; 23.1; 22.8; 17.4; 14.2; 1.1. ^{29}Si NMR (99 MHz, $CDCl_3$): 0.5, -0.1. MS (EI) [m/z (relat. int. %)]: 293.0 (10), 241.2 (22), 131.0 (100), 128.2 (10), 119.2 (33), 103.0 (10), 91.0 (18), 66.2 (29). HRMS (M/Z) calcd. for $C_{18}H_{32}O_2Si$: 308.2172; found 308.2171.

2-exo,3-exo-dimethyl[2,3-bis(trimethylsilyloxy]-5-norbornene —**M7**— was obtained according to the preparation procedure mentioned in the article, using 0.15 g (0.97 mmol) of diol (p = 97%), 411 μL (0.3 g; 2.96 mmol) of triethylamine (p = 99%) and 0.285 g (0.333 mL; 2.62 mmol) of chlorosilane (p = 99%) in 5 mL of THF, mixed for 2 h, with 78% yield (0.226 g; 0.76 mmol) as yellow liquid.

*Analytical data **M7***: ^{1}H NMR (400 MHz, $CDCl_3$): δ 6.15 (t, 2H, -C*H*=C*H*- (A), J_{H-H}=2.4Hz); 3.79 (dd, 2H, -C*H*$_2$- (B), J_{H-H}=5.6, 10.0Hz); 3.43 (dd, 2H, -C*H*$_2$- (C), J_{H-H}=8.8, 10.0Hz); 2.73 (p, 2H, -C*H*- (D), J_{H-H}=2.4Hz); 1.69-1.62 (m, 2H, -C*H*- (E)); 1.58 (s, water); 1.45 (dt, 1H, -C*H*$_2$- (F), J_{H-H}=1.6, 8.8Hz); 1.23 (dt, 1H, -C*H*$_2$- (G), J_{H-H}=1.6, 8.8Hz); 0.11 (s, 18H, -C*H*$_3$ (H)). ^{13}C NMR (105 MHz, $CDCl_3$): δ 137.6; 63.7; 44.6; 43.1; 42.4; -0.3. ^{29}Si NMR (99 MHz, $CDCl_3$): δ 20.2; 16.9; 7.3. MS (EI) [m/z (relat. int. %)]: 297.8 ($M^{+\bullet}$) (2), 146.9 (56), 143.0 (84), 129.0 (37), 91.0 (24), 73.0 (100), 69.0 (38), 66.0 (33), 45.0 (25). MS (FAB, m/z (%) (M^+, 100): 299. HRMS (M/Z) calcd. for $C_{15}H_{30}O_2Si_2$: 298.1784; found 298.1783.

2-exo,3-exo-dimethyl[2,3-bis(triethylsilyloxy]-5-norbornene —**M8**— was obtained according to the preparation procedure mentioned in the article, using 0.15 g (0.97 mmol) of diol (p = 97%), 411 μL (0.3 g; 2.96 mmol) of triethylamine (p = 99%) and 0.45 g (0.5 mL; 2.99 mmol) of chlorosilane (p = 98%) in 5 mL of THF, mixed for 2 h, with 80% yield (0.298 g; 0.78 mmol) as colorless liquid.

*Analytical data **M8***: ^{1}H NMR (400 MHz, $CDCl_3$): δ 6.15 (t, 2H, -C*H*=C*H*- (A), $J_{H\text{-}H}$=2.8Hz); 3.83 (dd, 2H, -C*H*$_2$- (B), $J_{H\text{-}H}$=7.2, 13.6Hz); 3.49 (dd, 2H, -C*H*$_2$- (C), $J_{H\text{-}H}$=11.2, 13.6Hz); 2.75 (p, 2H, -C*H*- (D), $J_{H\text{-}H}$=2.4Hz); 1.69-1.59 (m, 2H, -C*H*- (E)); 1.48 (d, 1H, -C*H*$_2$- (F), $J_{H\text{-}H}$=11.6Hz); 1.22 (dt, 1H, -C*H*$_2$- (G), $J_{H\text{-}H}$=2.4, 11.6Hz); 0.96 (t, 18H, -C*H*$_3$ (H), $J_{H\text{-}H}$=10.8Hz), 0.60 (q, 12H, -C*H*$_2$- (I), $J_{H\text{-}H}$=10.0Hz); 0.07 (s, silicone grease). ^{13}C NMR (105 MHz, $CDCl_3$): δ 137.7; 65.7; 46.1; 44.1; 43.4; 6.9; 6.6. ^{29}Si NMR (99 MHz, $CDCl_3$): δ 18.3. MS (EI) [m/z (relat. int. %)]: 383.0 ($M^{+\bullet}$) (2), 353.2 (27), 316.2 (27), 315.2 (100), 251.5 (64), 219.0 (20), 119.2 (73), 118.2 (36). MS (FAB, m/z (%) (M^+, 100): 382. HRMS (M/Z) calcd. for $C_{21}H_{42}O_2Si_2$: 382.2723; found 382.2722.

5-norbornene-2-exo,3-exo-dimethyl[2,2,5,5-tetramethyl-1,6,2,3-dioxasilane] —**M10**— was obtained according to the preparation procedure mentioned in the article, using 0.15 g (0.97 mmol) of diol (p = 97%), 280 μL (0.2 g; 2 mmol) of triethylamine (p = 99%) and 0.209 g (0.97 mmol) of dichlorosilane (p = 97%) in 5 mL of THF, mixed for 2 h, with 96% yield (0.276 g; 0.931 mmol) as slightly white oleic liquid.

*Analytical data **M10***: ^{1}H NMR (400 MHz, $CDCl_3$): δ 6.18 (t, 2H, -C*H*=C*H*- (A), $J_{H\text{-}H}$=2.4Hz); 3.98 (m, 2H, -CH2- (B)); 3.76-3.72 (m, 2H, -CH2- (C)); 2.52 (p, 2H, -C*H*- (D), $J_{H\text{-}H}$=2.0Hz); 1.88-1.80 (m, 2H, -C*H*- (E)); 1.35 (dt, 1H, -C*H*$_2$- (F), $J_{H\text{-}H}$=2.4, 12.0Hz); 1.24 (dt, 1H, -C*H*$_2$- (G), $J_{H\text{-}H}$=2.0, 12.0Hz); 0.41 (s, 4H, -C*H*$_2$- (H)); 0.03 (s, 12H, -C*H*$_3$ (I)). ^{13}C NMR (105 MHz, $CDCl_3$): δ 137.6; 65.1; 45.9; 44.0; 43.5; 10.0; -0.3. ^{29}Si NMR (99 MHz, $CDCl_3$): δ 17.0. MS (EI) [m/z (relat. int. %)]: 296.8 ($M^{+\bullet}$) (12), 281.0 (14), 162.0 (26), 161.2 (100), 156.2 (19), 145.0 (16), 119.0 (11), 91.0 (13), 66.0 (17), 69.0 (13). HRMS (M/Z) calcd. for $C_{15}H_{28}O_2Si_2$: 296.1628; found 296.1625.

Polymer synthesis

Poly(norbornene-2-exo,3-exo-dimethyl-[(2,2-diphenyl-1,3,2-dioxasilane])s —**P1**— was obtained according to the preparation procedure mentioned in the article, using 0.138 g of monomer **M1**, 0.02 M solution of 1st generation Grubbs catalyst (207 µL; 4.13x10^{-6} mol), in 16.5 mL of dichloromethane, mixed for 3 h at rt, with 97% yield (0.134 g) as white solid.

*Analytical data **P1***: ^{1}H NMR (400 MHz, $CDCl_3$): δ 7.70-7.30 (br d, 10H, Ar-*H* (A)); 5.36-5.24 (br d, 2H, -C*H*=C*H*- (B)); 3.95-3.84 (br d, 4H, -C*H*$_2$- (C)); 2.58 (br s, 2H, -C*H*- (D)); 2.21-1.92 (br t, 2H, -C*H*- (E)); 1.56 (s, water); 1.28 (br s, 2H, -C*H*$_2$- (F)); 1.26 (s, grease) 0.86 (m, grease). ^{13}C NMR (105 MHz, $CDCl_3$): δ 134.8; 134.5; 134.1; 130.5; 128.1; 127.9; 63.2; 50.3; 49.8; 44.2; n-hexane: 31.7; 22.8; 14.3. ^{29}Si NMR (99 MHz, $CDCl_3$): δ -25.5. Elemental Analyses calcd. for $C_{22}H_{23}O_2Si$: C 76.04, H 6.67; found C 76.02, H 6.66.

Poly(norbornene-2-exo,3-exo-dimethyl-[(2-methyl-2-phenyl-1,3,2-dioxasilane])s —**P3**— was obtained according to the preparation procedure mentioned in the article, using 0.142 g of monomer **M3**, 0.02 M solution of 1st generation Grubbs catalyst (261 µL; 5.213x10^{-6} mol), in 11.0 mL of dichloromethane, mixed for 1.5 h at rt, with 93% yield (0.132 g) as white-beige solid.

*Analytical data **P3***: ^{1}H NMR (400 MHz, $CDCl_3$): δ 7.74-7.29 (br d, 5H, Ar-*H* (A)); 5.46-5.16 (br t, 2H, -C*H*=C*H*- (B)); 3.92-3.70 (br d, 4H, -C*H*$_2$- (C)); 2.45 (br s, 2H, -C*H*- (D)); 2.20-1.76 (br d, 2H, -C*H*- (E)); 1.26 (br s, 2H, -C*H*$_2$- (F)); 0.89 (t, grease); 0.56-0.24 (br m, 3H, -C*H*$_3$ (G)). ^{13}C NMR (105 MHz, $CDCl_3$): δ 134.3; 133.5; 130.0; 128.2; 128.1; 127.9; 63.3; 50.4; 49.7; 44.6; -0.1; n-hexane: 31.7; 22.8; 14.3. ^{29}Si NMR (99 MHz, $CDCl_3$): δ -10.5. Elemental Analyses calcd. for $C_{17}H_{21}O_2Si$: C 71.53, H 7.42; found C 71.51, H 7.40.

Poly(norbornene-2-exo,3-exo-dimethyl-[(2,2-diethyl-1,3,2-dioxasilane])s —**P4**— was obtained according to the preparation procedure mentioned in the article, using 0.05 g of monomer **M4**, 0.02 M solution of 1st generation Grubbs catalyst (105 µL; 2.097x10^{-6} mol), in 4.0 mL of dichloromethane, mixed for 1.5 h at rt, with 85% yield (0.0425 g) as white-beige solid.

*Analytical data **P4***: ^{1}H NMR (400 MHz, $CDCl_3$): δ 5.33-5.32 (br d, 2H, -C*H*=C*H*- (A)); 3.81 (br s, 4H, -C*H*$_2$- (B)); 2.58 (br s, 2H, -C*H*- (C)); 2.20-2.00 (br d, 2H, -C*H*- (D)); 1.25 (br s, 2H, -C*H*$_2$- (E)); 0.98 (t, 6H, -C*H*$_3$ (F), J_{H-H}=8.0Hz); 0.59 (q, 4H, -C*H*$_2$- (G), J_{H-H}=8.0Hz); 0.06 (s, silicone grease). ^{13}C NMR (105 MHz, $CDCl_3$): δ 133.5; 62.5; 50.5; 49.9; 44.1; 7.5; 6.4; n-hexane: 31.7; 22.8; 14.3. ^{29}Si NMR (99 MHz, $CDCl_3$): δ 1.1; -9.2. Elemental Analyses calcd. for $C_{14}H_{23}O_2Si$: C 66.88; H 9.22; found C 66.74, H 9.19.

Poly(norbornene-2-exo,3-exo-dimethyl-[(2,2-diisopropyl-1,3,2-dioxasilane])s—**P5**— was obtained according to the preparation procedure mentioned in the article, using 0.17 g of monomer **M5**, 0.02 M solution of 1st generation Grubbs catalyst (320 µL; 6.38x10^{-6} mol), in 12.5 mL of dichloromethane, mixed for 1 h at rt, with 79% yield (0.134 g) as beige solid.

*Analytical data **P5***: ^{1}H NMR (400 MHz, $CDCl_3$): δ 5.32-5.23 (br d, 2H, -C*H*=C*H*- (A)); 3.84-3.71 (br d, 4H, -C*H*$_2$- (B)); 2.55 (br s, 2H, -C*H*- (C)); 2.14-1.87 (br t, 2H, -C*H*- (D)); 1.25 (br s, 2H, -C*H*$_2$- (E)); 1.06-0.83 (m, 14H, -C*H*-(C*H*$_3$)$_2$) (F,G); 0.06 (s, silicone grease). ^{13}C NMR (105 MHz, $CDCl_3$) δ 133.4; 63.1; 50.5; 50.0; 44.3; 17.6; 17.3; 12.2; 11.8. ^{29}Si NMR (99 MHz, $CDCl_3$): δ -4.2. Elemental Analyses calcd. for $C_{16}H_{27}O_2Si$: C 68.76, H 9.74; found C 68.35, H 9.73.

Poly(norbornene-2-exo,3-exo-dimethyl-[(2-methyl-2-octyl-1,3,2-dioxasilane])s —**P6**— was obtained according to the preparation procedure mentioned in the article, using 0.054 g of monomer **M6**, 0.02 M solution of 1st generation Grubbs catalyst (175 μL; 3.5x10^{-6} mol), in 4.7 mL of dichloromethane, mixed for 2 h at 30^{0}C, with 98% yield (0.053 g) as brown membrane.

*Analytical data **P6***: ^{1}H NMR (400 MHz, $CDCl_3$): δ 5.31-5.21 (br d, 2H, -C*H*=C*H*- (A)); 3.78 (br s, 4H, -C*H*$_2$- (B)); 2.57 (br s, 2H, -C*H*- (C)); 2.17-1.90 (br t, 2H, -C*H*- (D)); 1.26 (br s, 14H, -C*H*$_2$- (E)); 0.89-0.88 (br t, 3H, -C*H*$_3$ (F)); 0.68-0.49 (br s, 2H, -C*H*$_2$ (G)); 0.13-0.05 (m, 3H, -C*H*$_3$ (H)). ^{13}C NMR (105 MHz, $CDCl_3$): δ 133.5; 62.3; 50.2; 49.8; 44.0; 33.2; 32.1; 29.4; 26.6; 25.8; 22.8; 17.4; 14.2; 1.2. ^{29}Si NMR (99 MHz, $CDCl_3$): 0.50, -0.14. Elemental Analyses calcd. for $C_{19}H_{33}O_2Si$: C 70.97, H 10.34; found C 71.01, H 10.28.

Poly(norbornene-2-exo,3-exo-dimethyl-(2,3-bis(trimethylsilyloxy))s —**P7**— was obtained according to the preparation procedure mentioned in the article, using 0.057 g of monomer **M7**, 0.02 M solution of 1st generation Grubbs catalyst (95 μL; 1.91x10^{-6} mol), in 5.0 mL of dichloromethane, mixed for 2 h at 30^{0}C, with 99% yield (0.056 g) as brown membrane.

*Analytical data **P7***: ^{1}H NMR (400 MHz, $CDCl_3$): δ 5.27-5.15 (br d, 2H, -C*H*=C*H*- (A)); 3.61-3.58 (br d, 4H, -C*H*$_2$- (B)); 2.64 (br s, 2H, -C*H*- (C)); 2.30 (br s, 2H, -C*H*- (D)); 1.88 (br s, 2H, -C*H*$_2$- (E)); 1.62 (s, water); 0.13-0.08 (m, 18H, -C*H*$_3$ (F)). ^{13}C NMR (105 MHz, $CDCl_3$): δ 133.8; 62.1; 49.7; 48.9; 44.9; 2.1; grease: 29.8. ^{29}Si NMR (99 MHz, $CDCl_3$): 19.8; 16.5; 6.9. Elemental Analyses calcd. for $C_{16}H_{31}O_2Si_2$: C 61.68, H 10.03; found C 61.34, H 10.14.

Poly(norbornene-2-exo,3-exo-dimethyl-(2,3-bis(triethylsilyloxy))s —**P8**—was obtained according to the preparation procedure mentioned in the article, using 0.15 g of monomer **M8**, 0.02 M solution of 1st generation Grubbs catalyst (196 μL; 3.92x10^{-6} mol), in 12.2 mL of dichloromethane, mixed for 2 h at rt, with 96% yield (0.144 g) as brown membrane.

*Analytical data **P8***: ^{1}H NMR (400 MHz, $CDCl_3$): δ 5.29-5.15 (br d, 2H, -C*H*=C*H*- (A)); 3.70-3.62 (br d, 4H, -C*H*$_2$- (B)); 2.45 (br s, 2H, -C*H* (C)); 1.87 (br s, 2+2H, (D,E)); 1.05-0.90 (br m, 18H, -C*H*$_3$ (F)); 0.63-0.51 (br m, 12H, -C*H*$_2$- (G)); 0.07 (s, silicone grease). ^{13}C NMR (105 MHz, $CDCl_3$): δ 134.1; 62.3; 50.3; 49.0; 44.7; 7.0; 4.7. ^{29}Si NMR (99 MHz, $CDCl_3$): δ 17.6. Elemental Analyses calcd. for $C_{22}H_{43}O_2Si_2$: C 66.77, H 10.95; found C 66.63, H 10.91.

NMR Spectra ^{1}H, ^{13}C, ^{29}Si NMR

MONOMERS

Compound **M1**

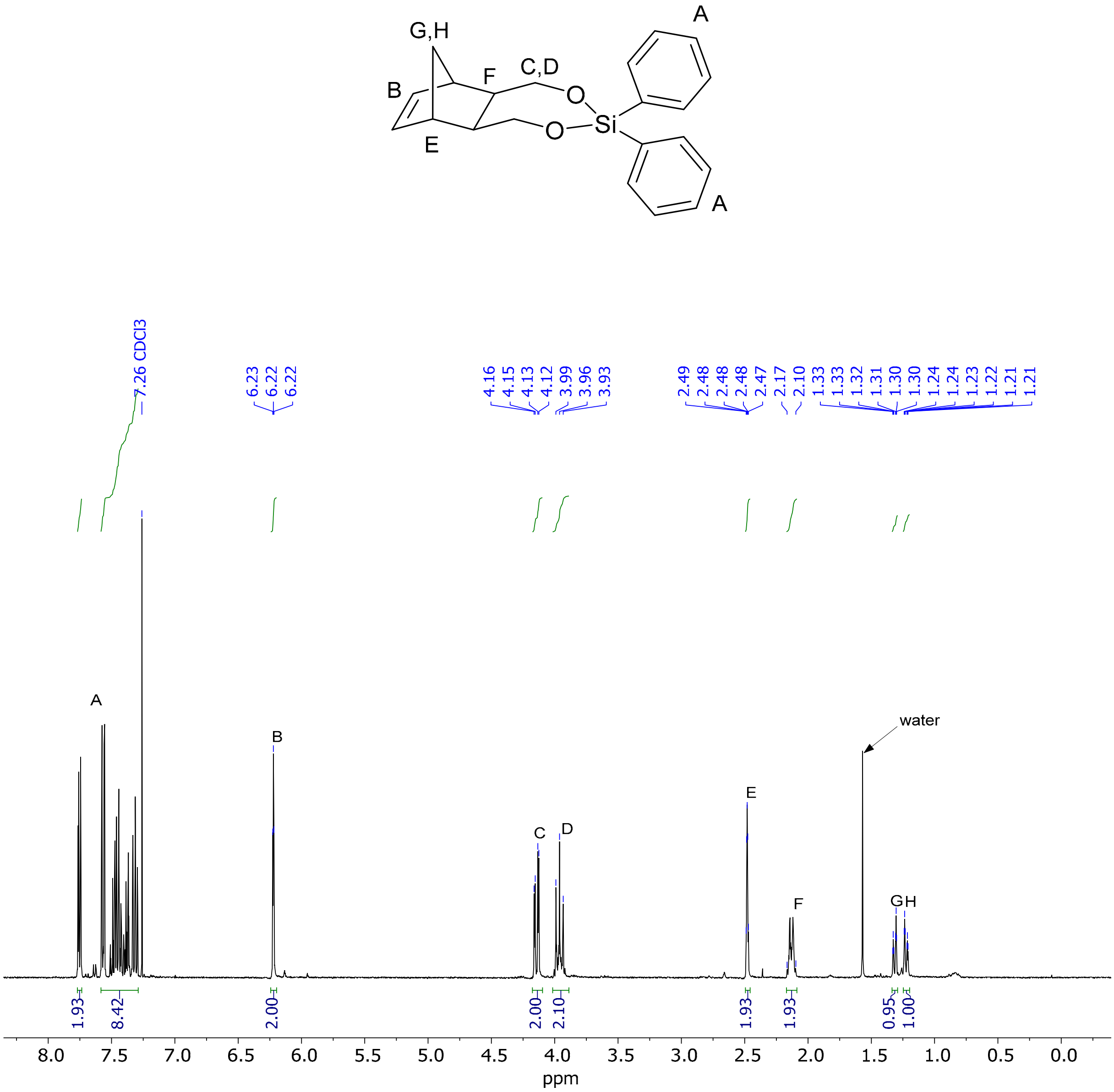

Figure S1. Compound **M1** – ^{1}H NMR in $CDCl_3$

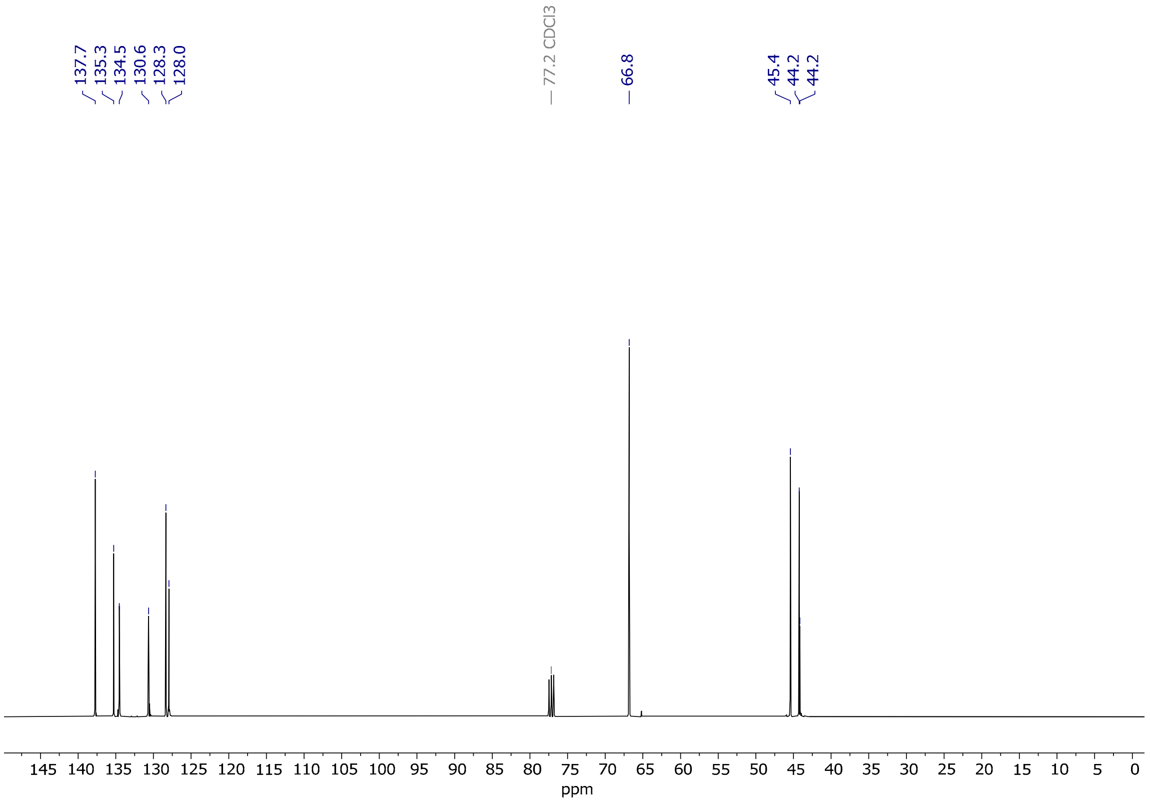

Figure S2. Compound **M1** – ^{13}C NMR in $CDCl_3$

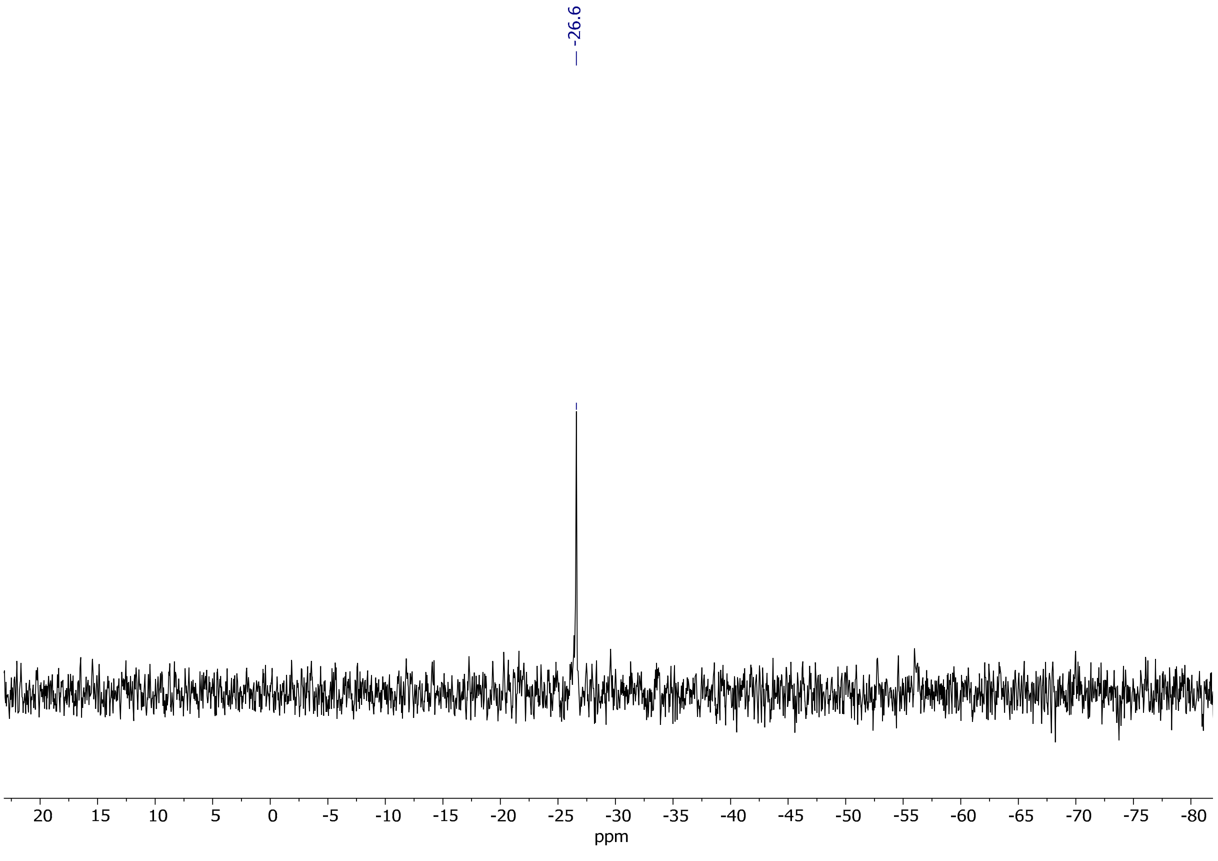

Figure S3. Compound **M1** – ^{29}Si NMR in $CDCl_3$

Compound **M2**

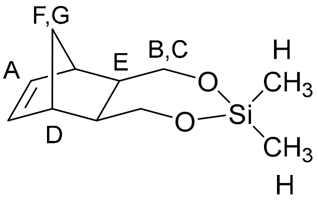

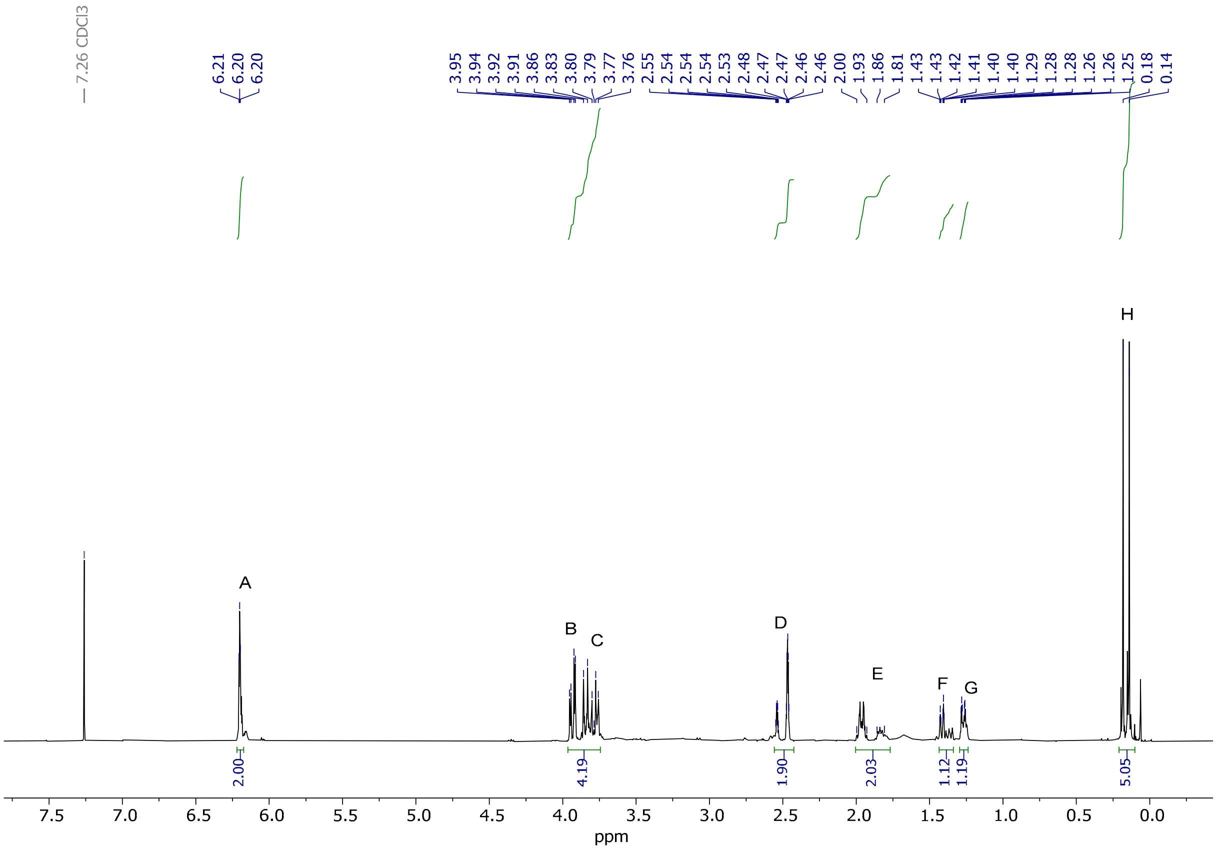

Figure S4. Compound **M2** – ^{1}H NMR in $CDCl_3$

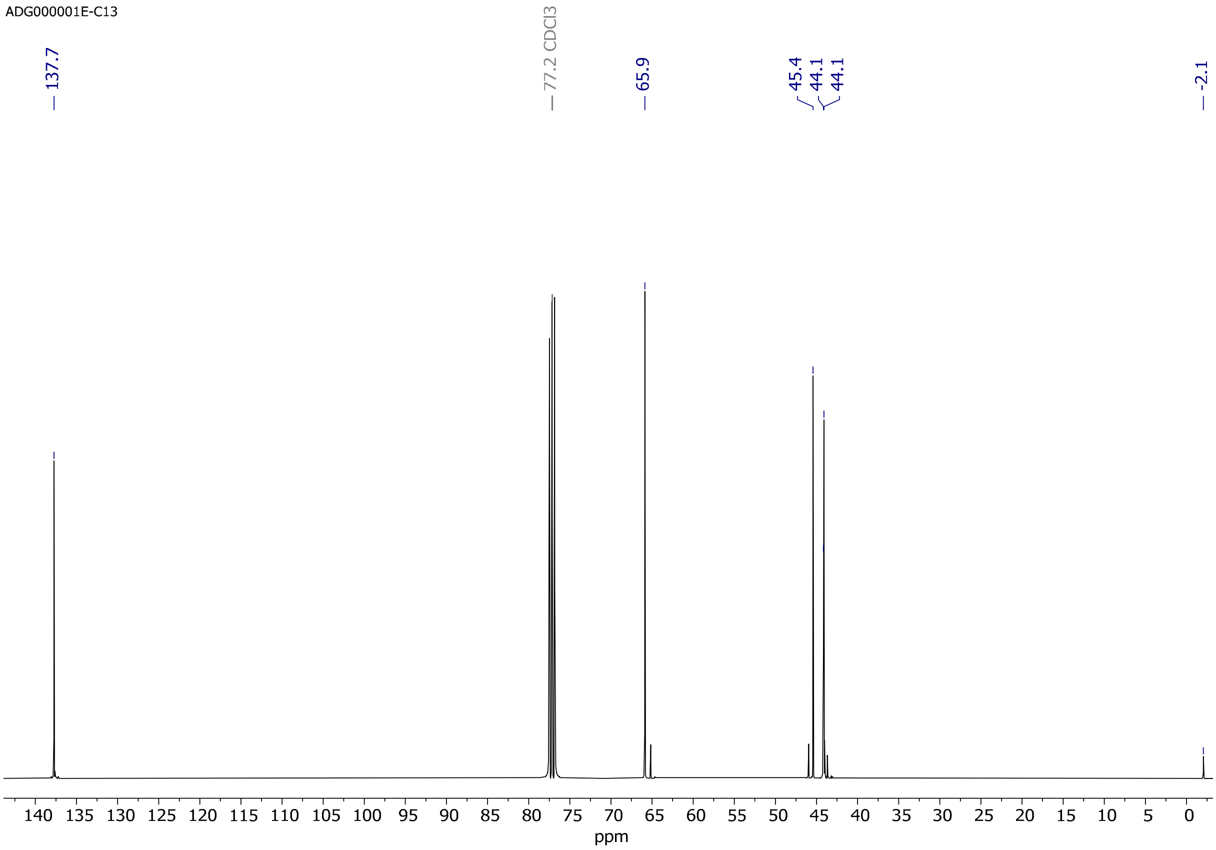

Figure S5. Compound **M2** – ^{13}C NMR in $CDCl_3$

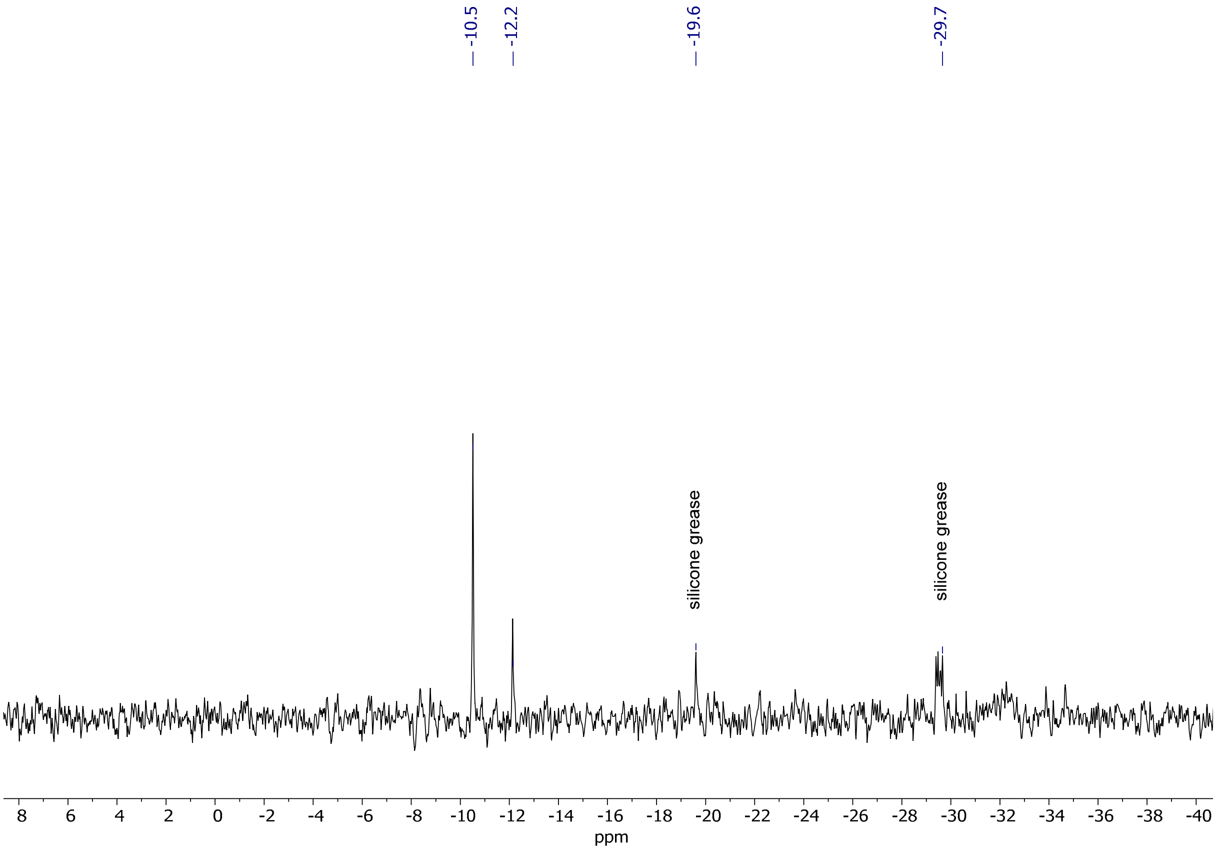

Figure S6. Compound **M2** – ^{29}Si NMR in $CDCl_3$

Compound **M3**

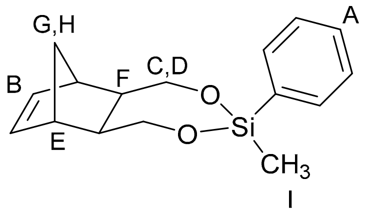

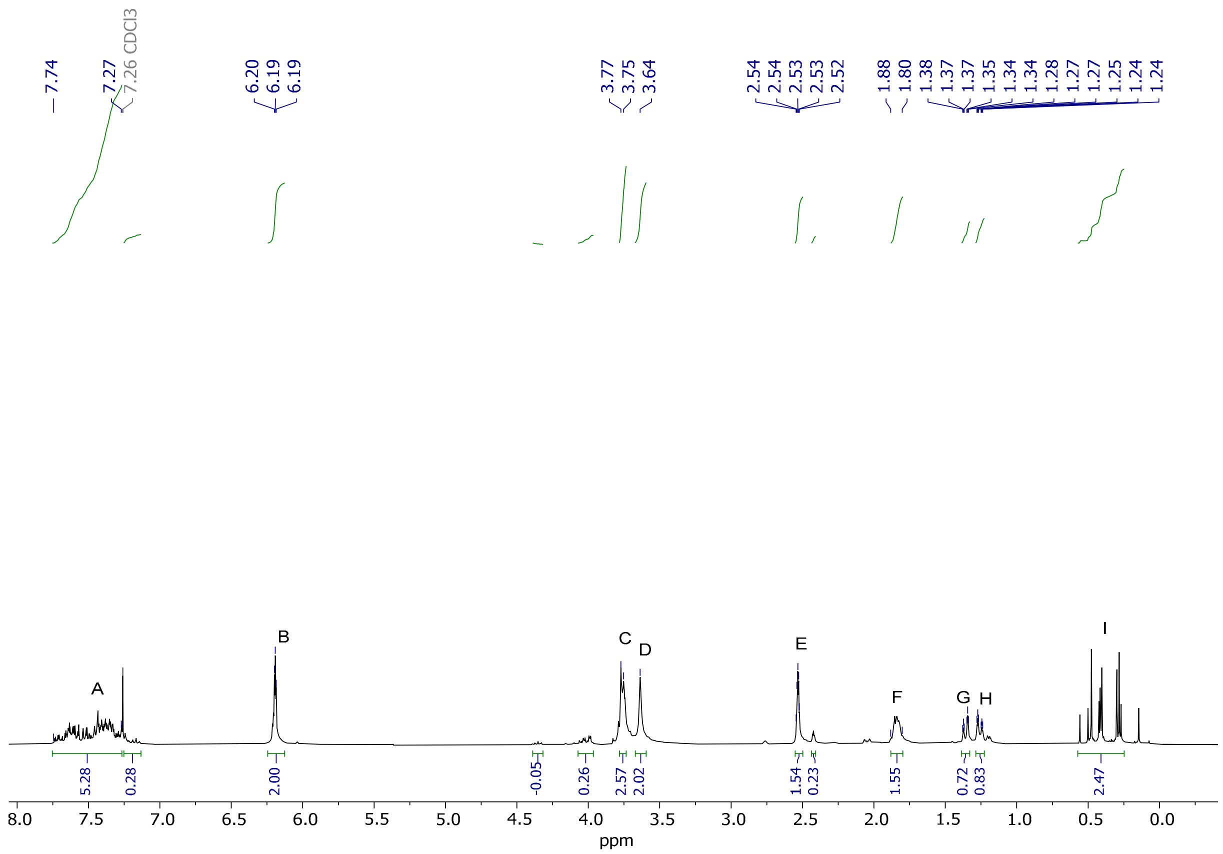

Figure S7. Compound **M3** – ^{1}H NMR in $CDCl_3$

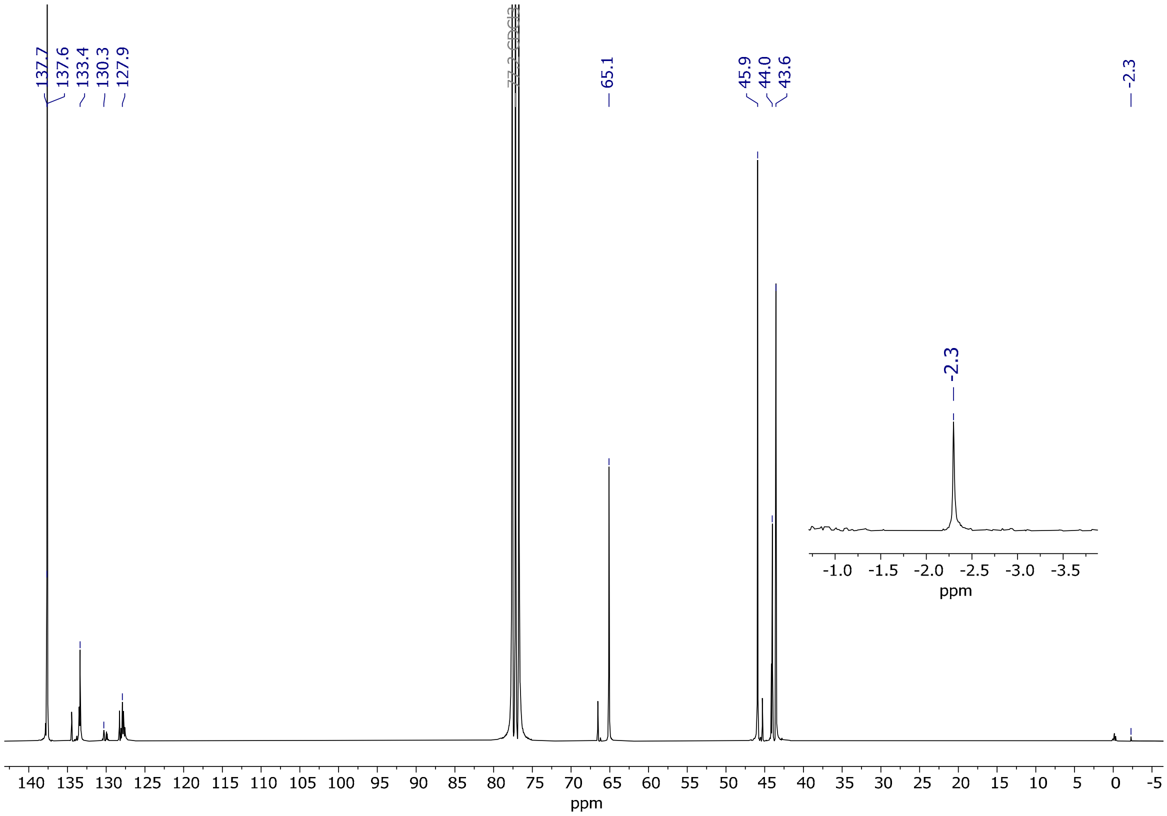

Figure S8. Compound **M3** – ^{13}C NMR in $CDCl_3$

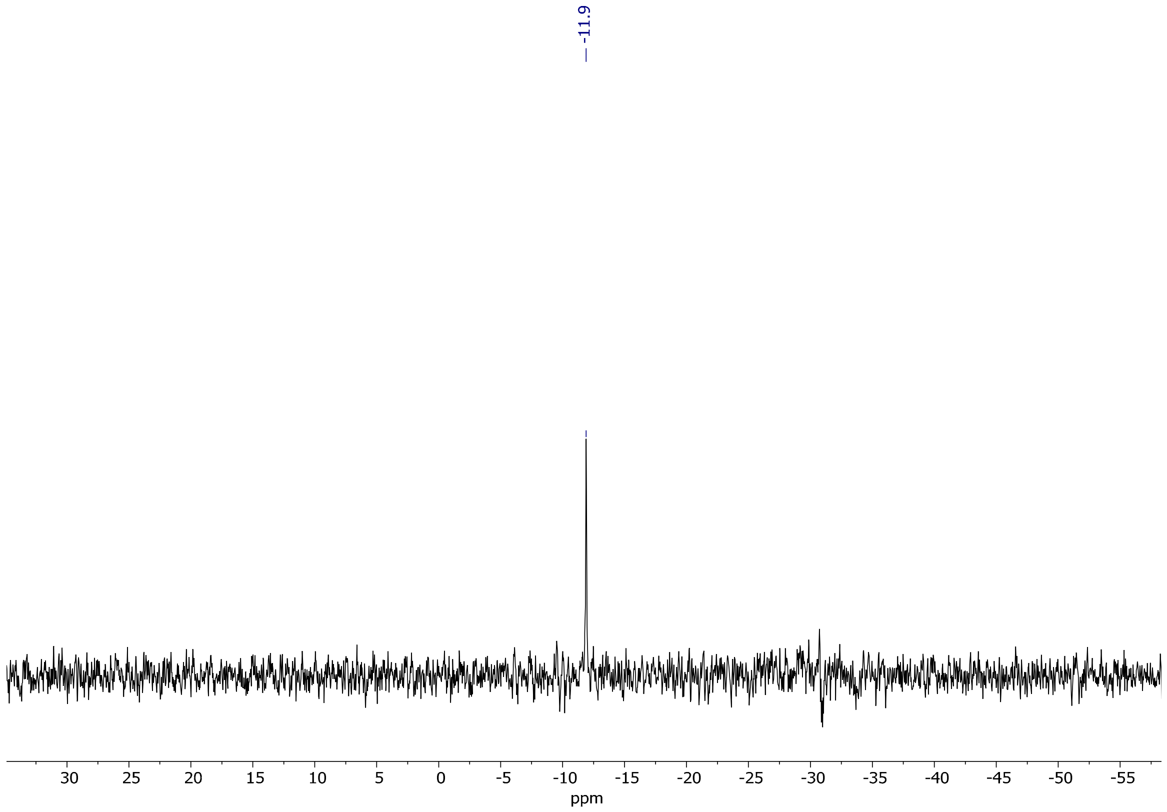

Figure S9. Compound **M3** – ^{29}Si NMR in $CDCl_3$

Compound **M4**

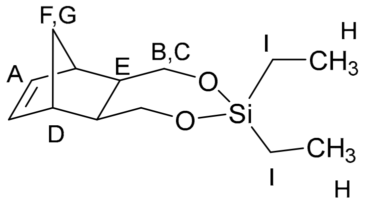

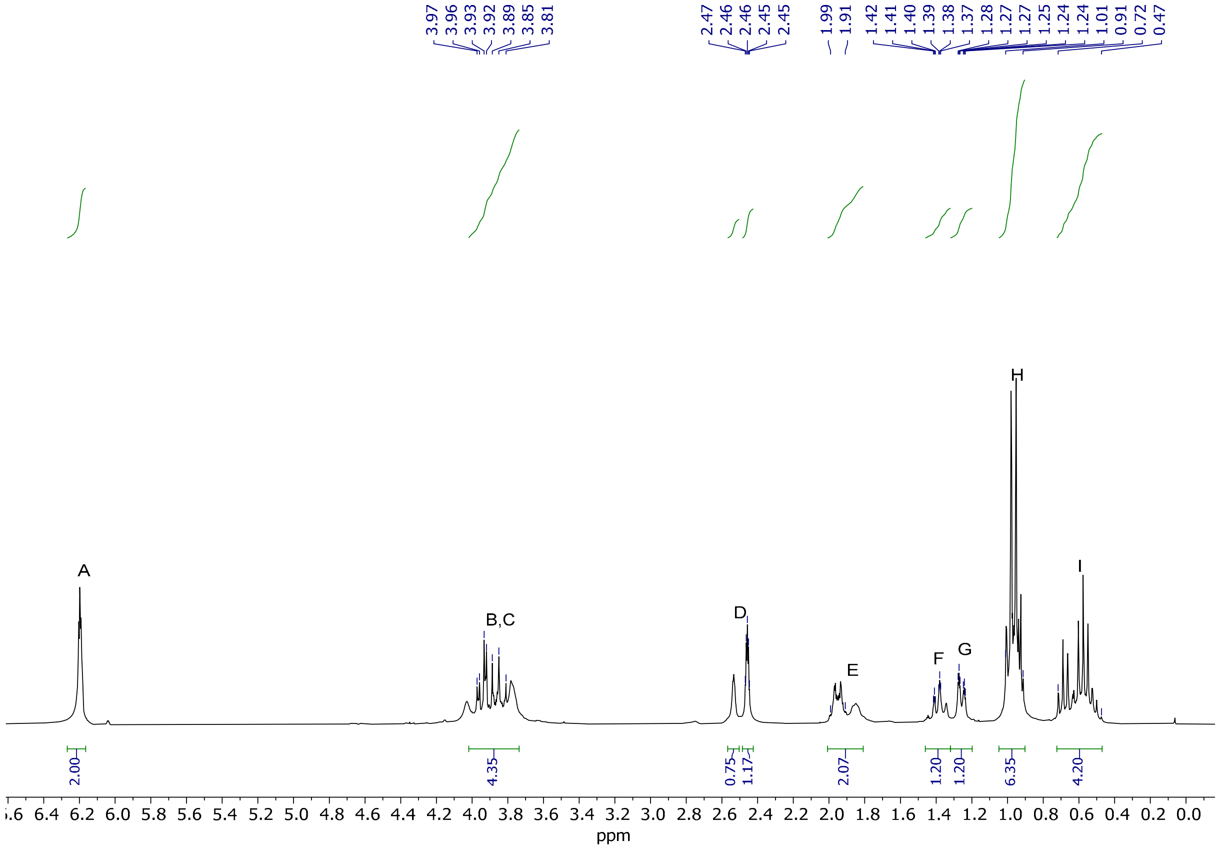

Figure S10. Compound **M4** – ^{1}H NMR in $CDCl_3$

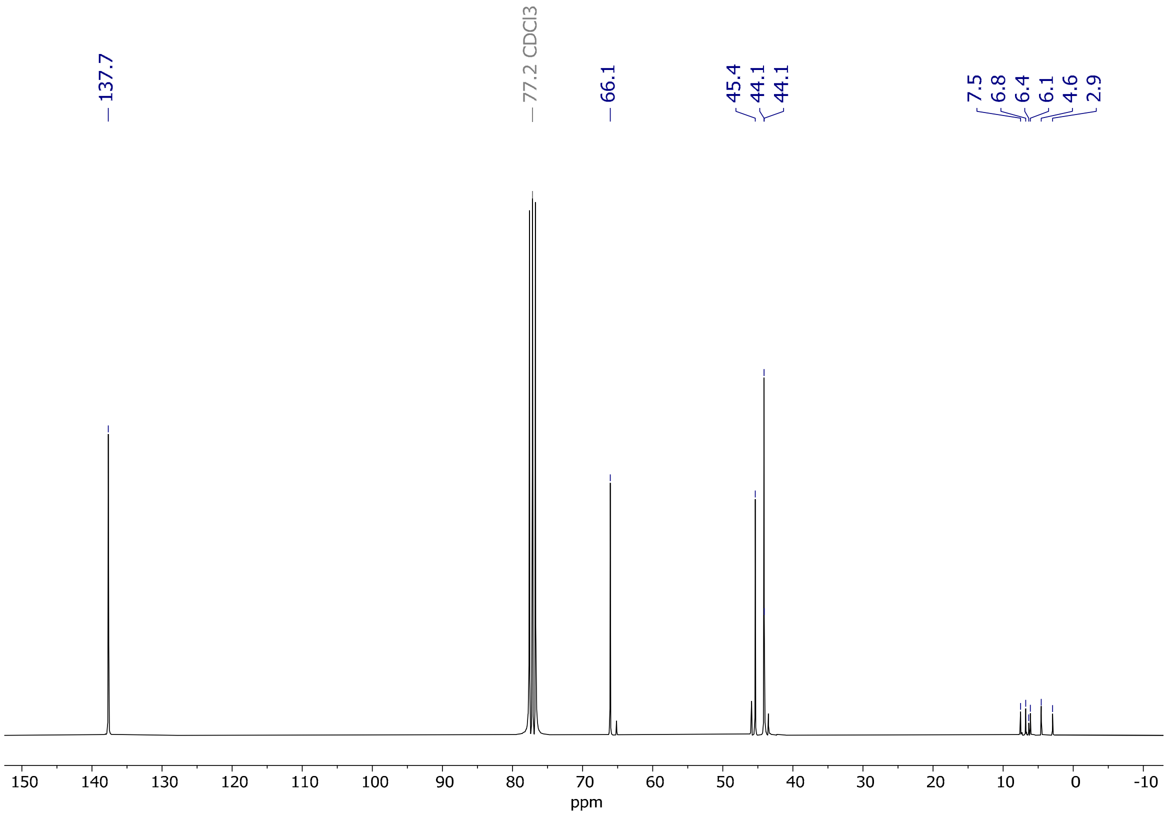

Figure S11. Compound **M4** – ^{13}C NMR in $CDCl_3$

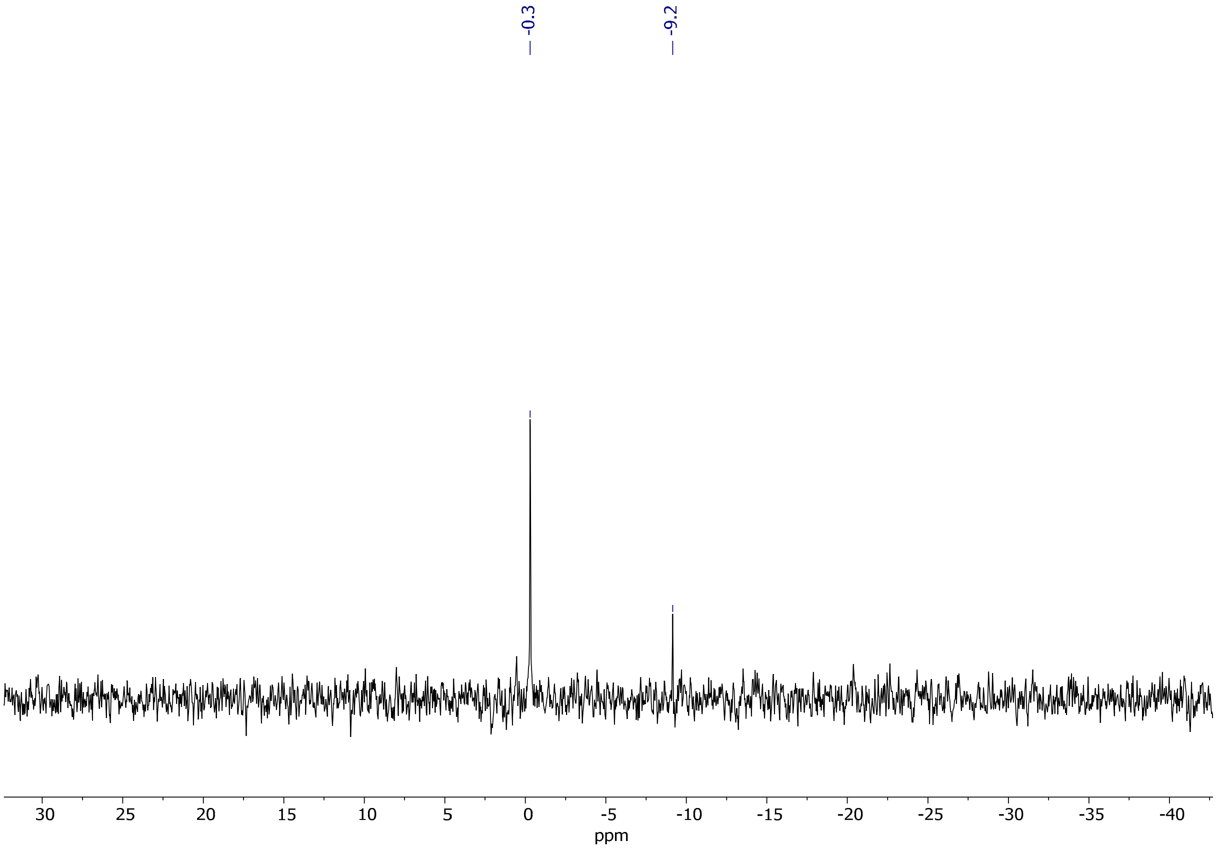

Figure S12. Compound **M4** – ^{29}Si NMR in $CDCl_3$

Compound **M5**

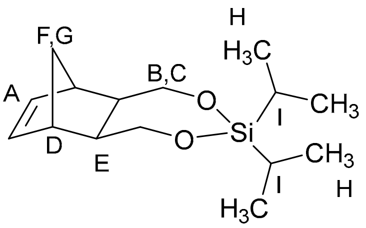

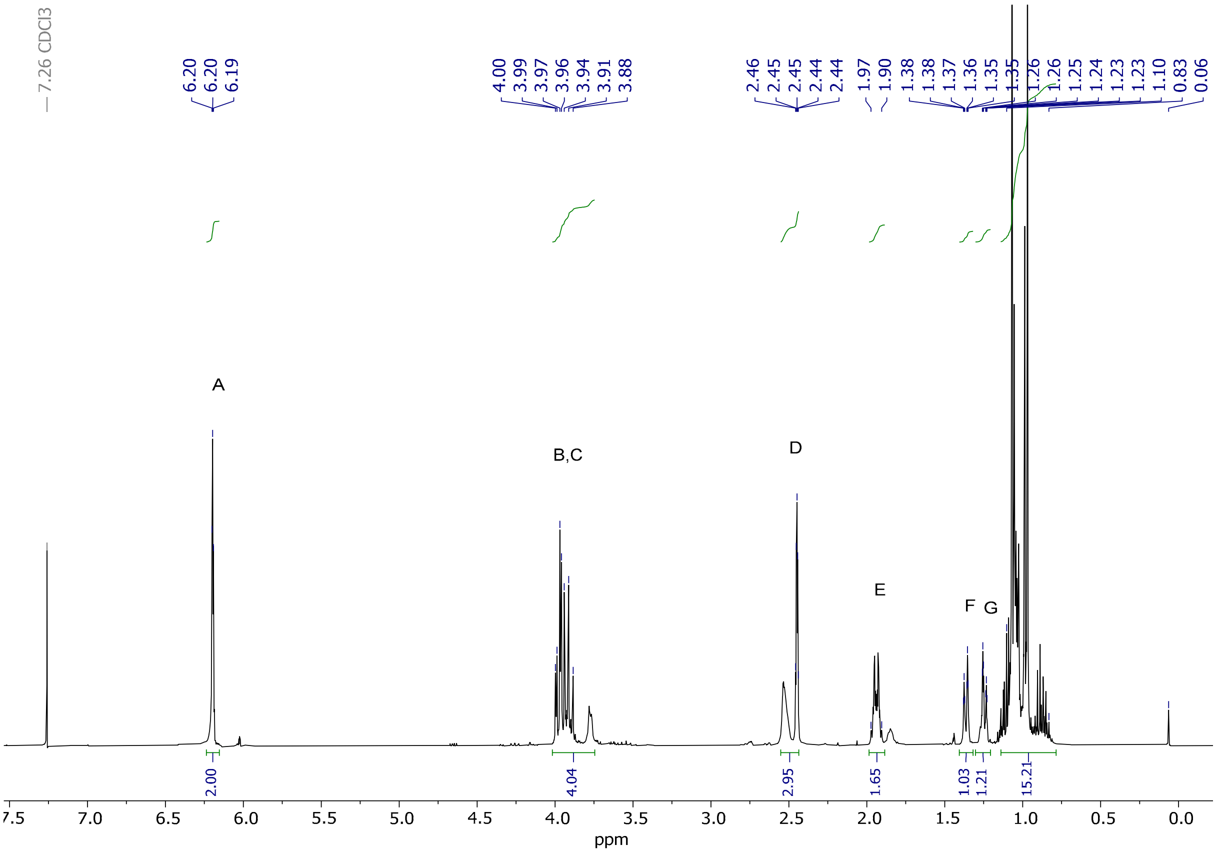

Figure S13. Compound **M5** – ^{1}H NMR in $CDCl_3$

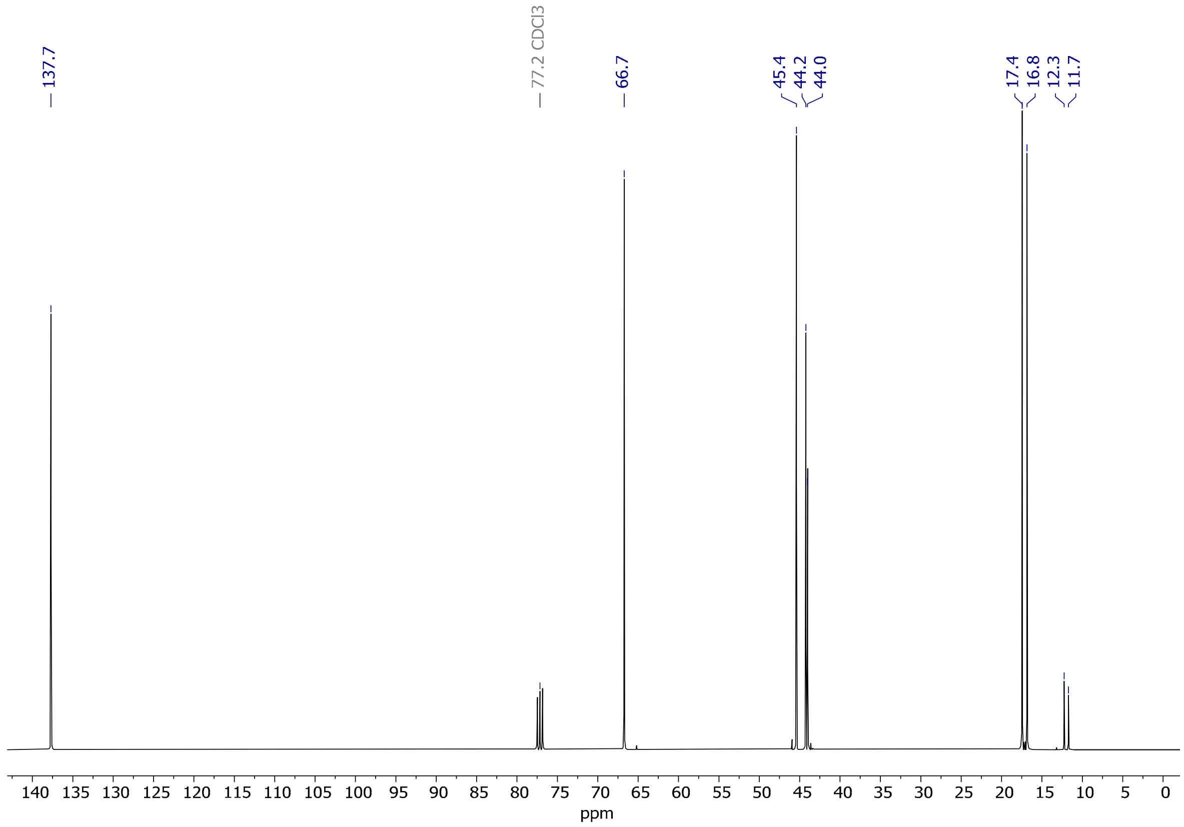

Figure S14. Compound **M5** – ^{13}C NMR in $CDCl_3$

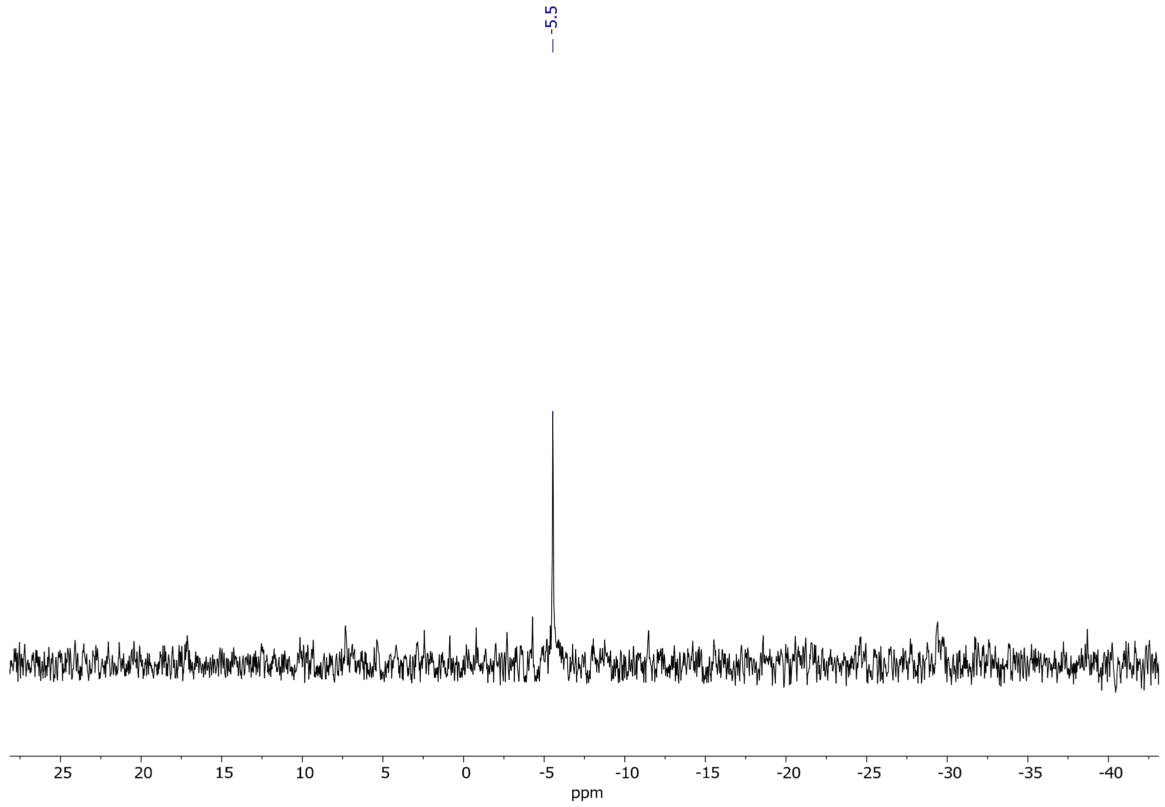

Figure S15. Compound **M5** – ^{29}Si NMR in $CDCl_3$

Compound **M6**

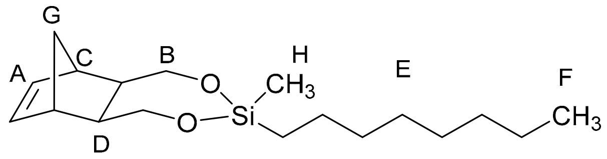

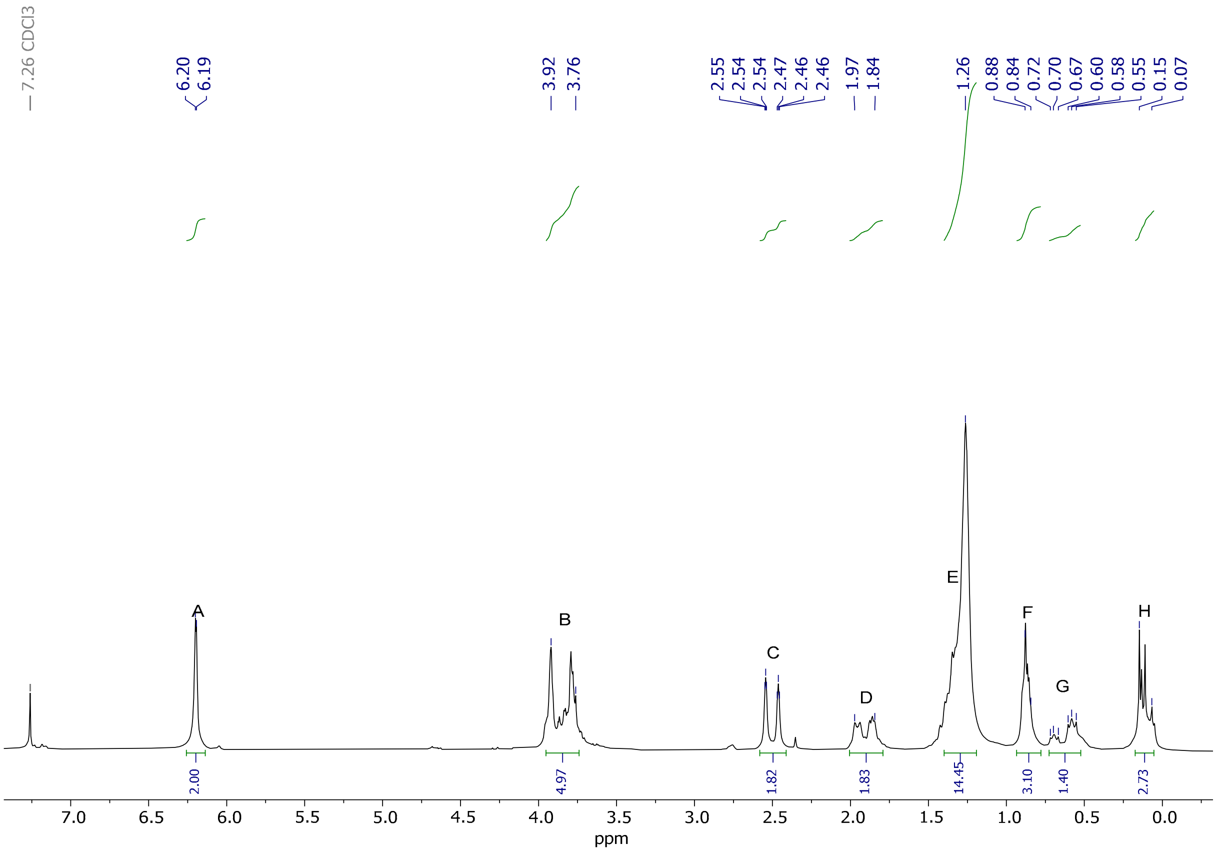

Figure S16. Compound **M6** – ^{1}H NMR in $CDCl_3$

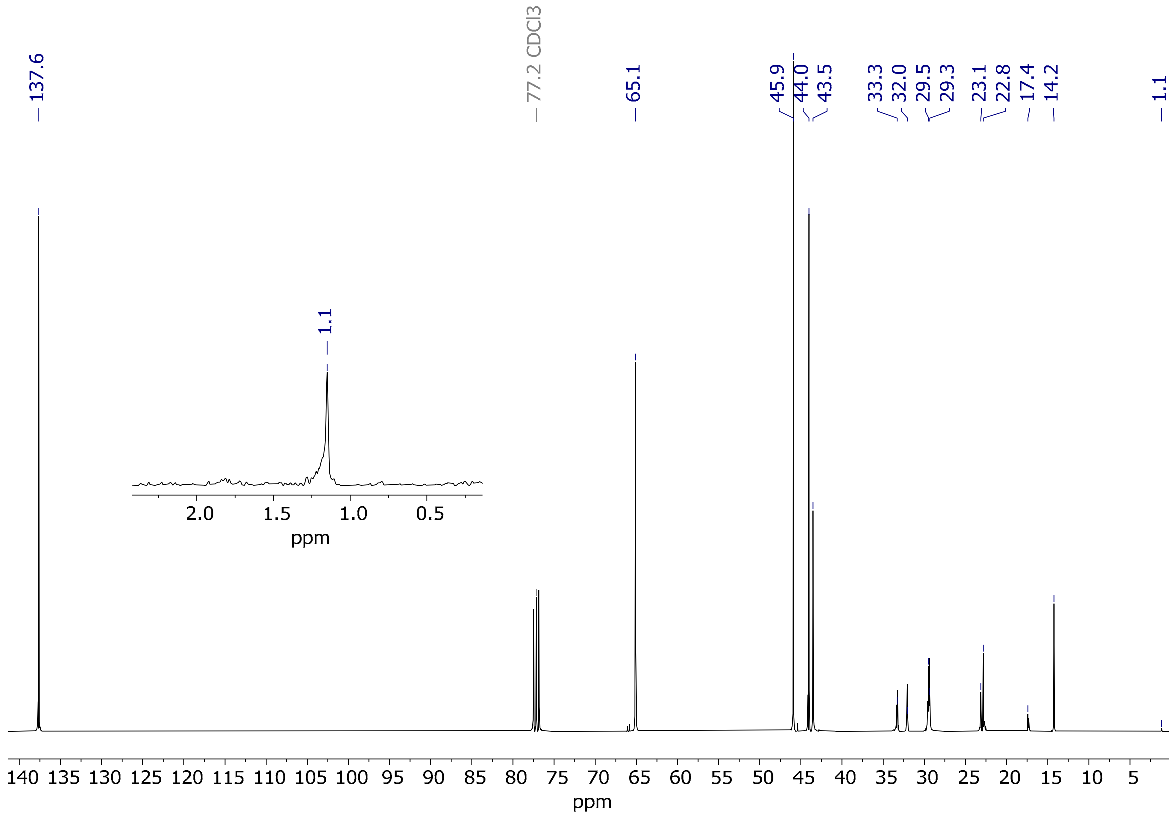

Figure S17. Compound **M6** – ^{13}C NMR in $CDCl_3$

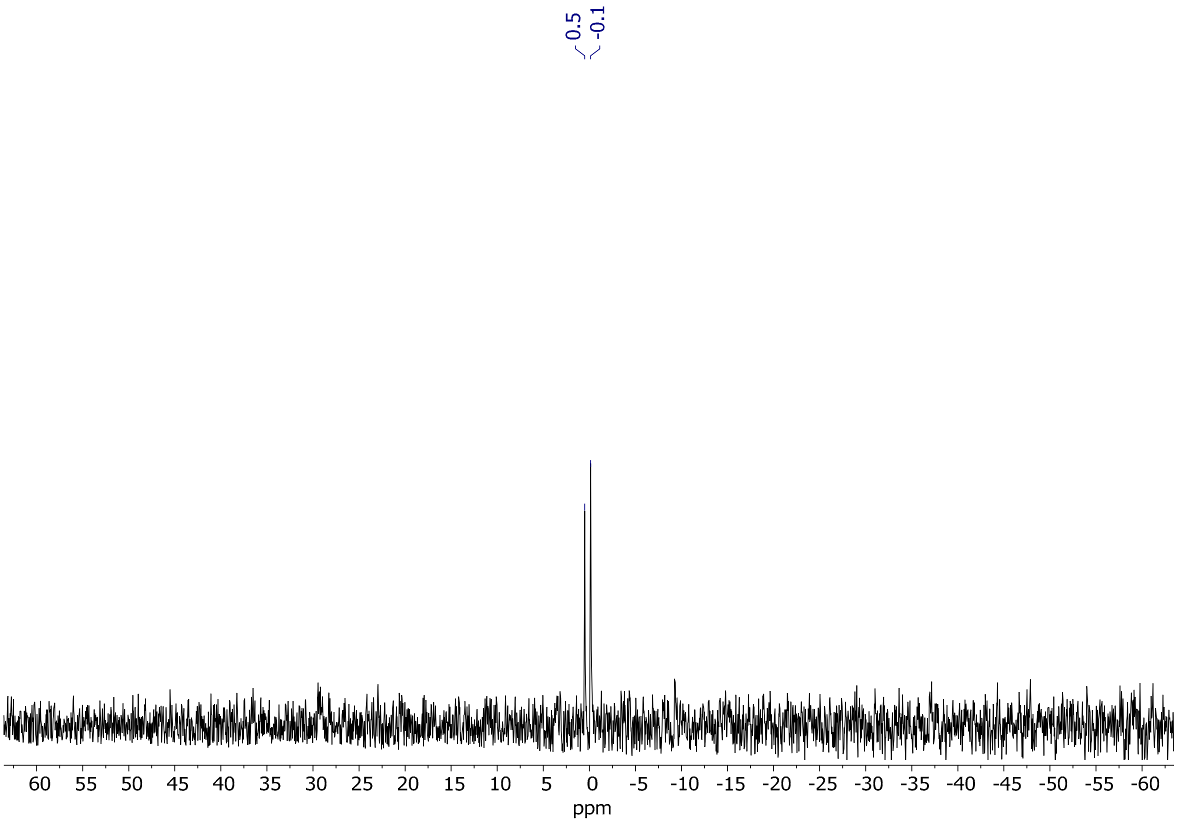

Figure S18. Compound **M6** – ^{29}Si NMR in $CDCl_3$

Compound **M7**

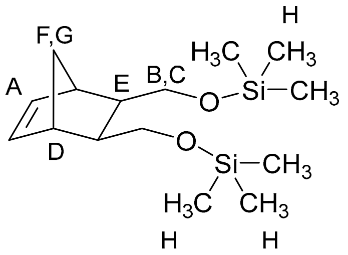

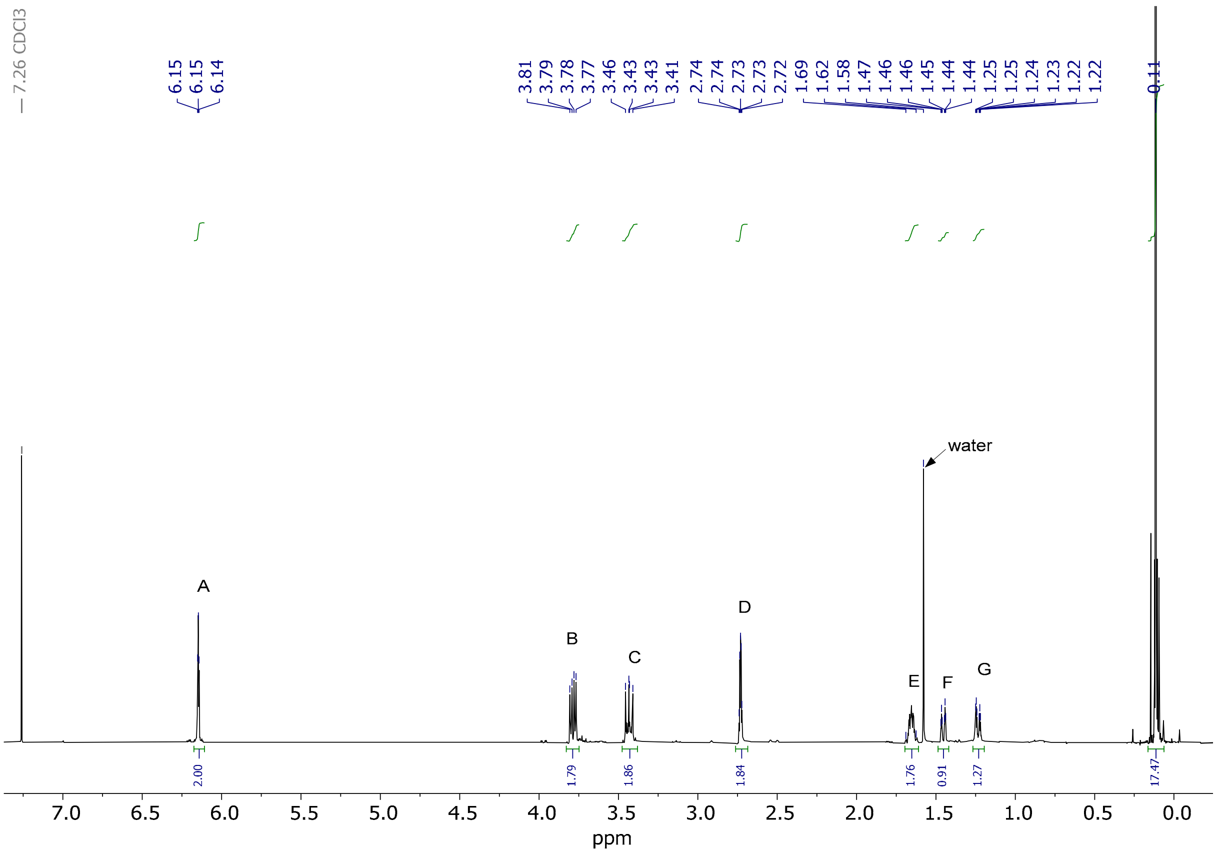

Figure S19. Compound **M7** – ^{1}H NMR in $CDCl_3$

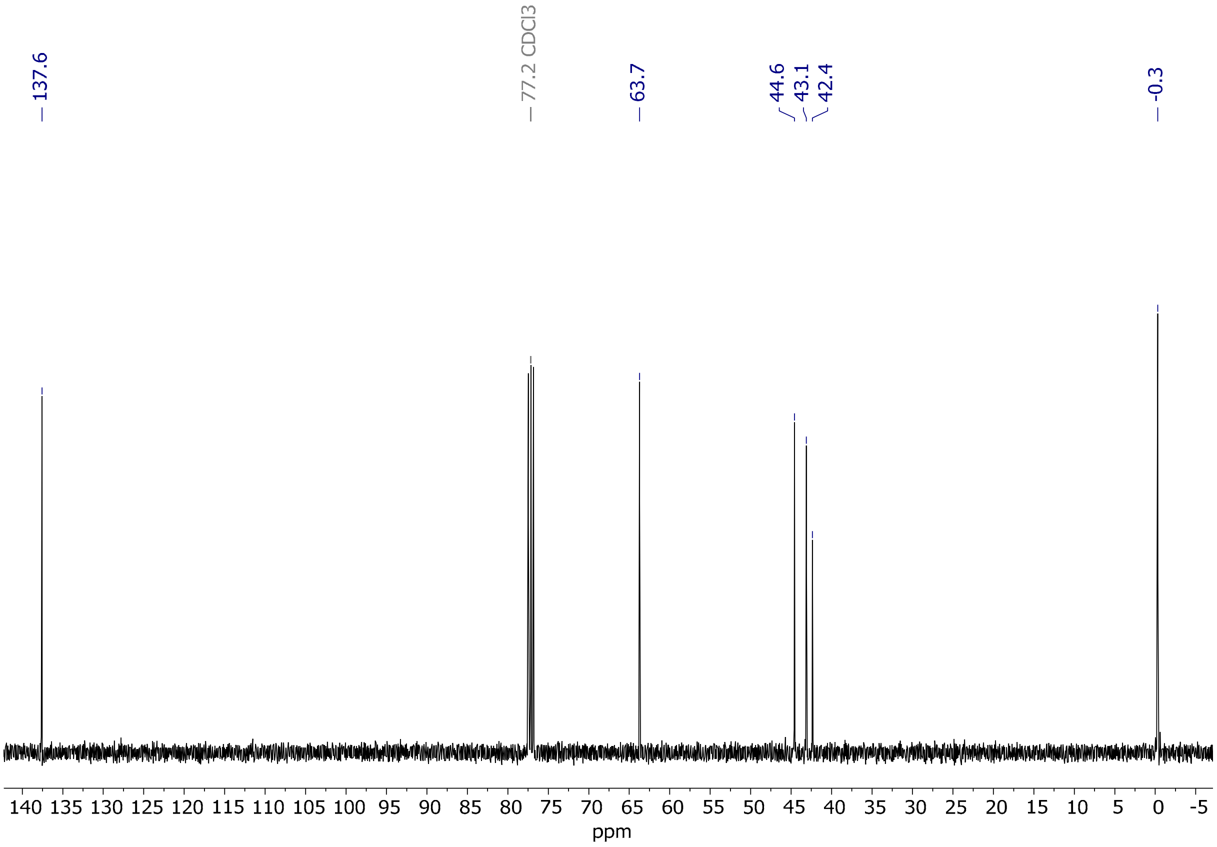

Figure S20. Compound **M7** – ^{13}C NMR in $CDCl_3$

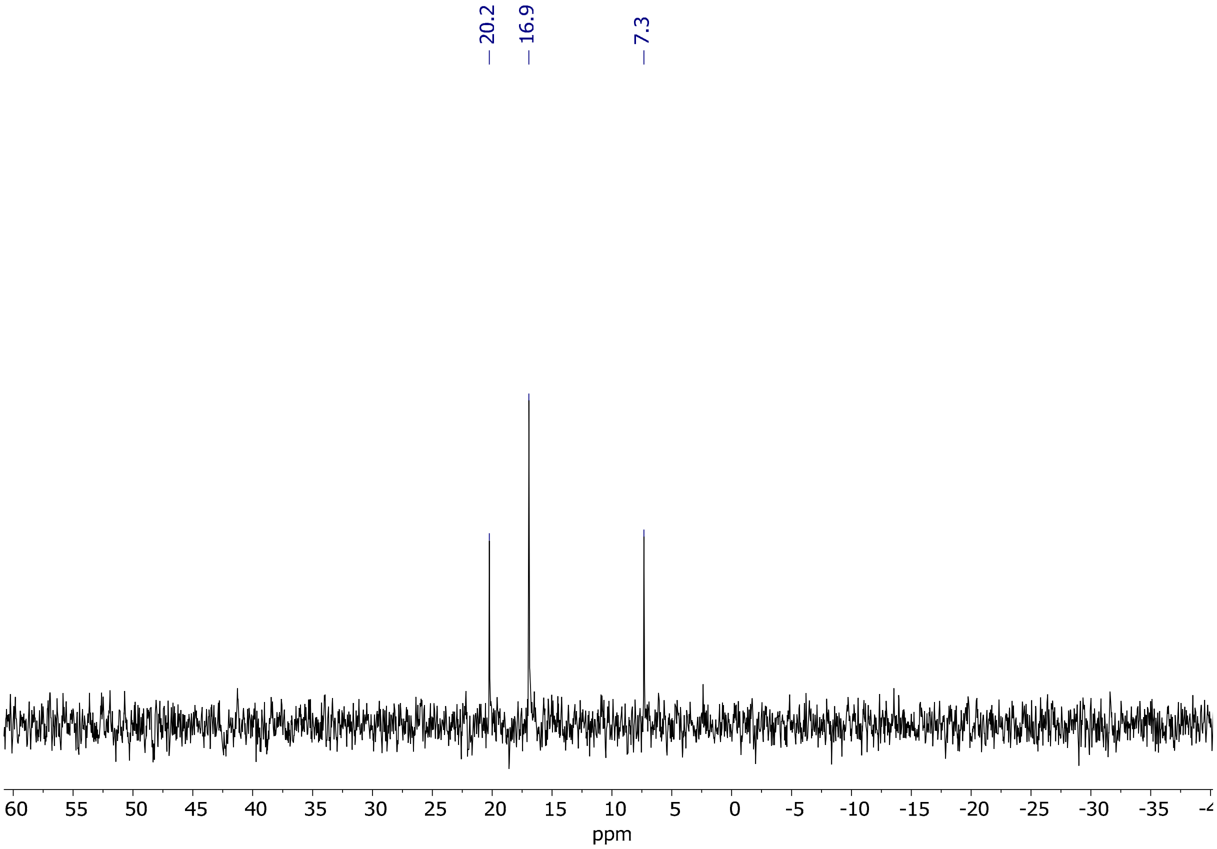

Figure S21. Compound **M7** – ^{29}Si NMR in $CDCl_3$

Compound **M8**

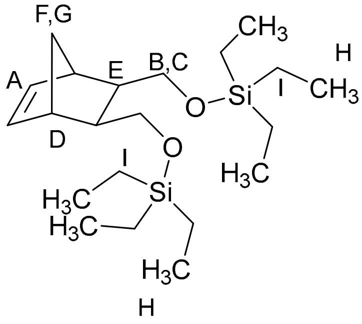

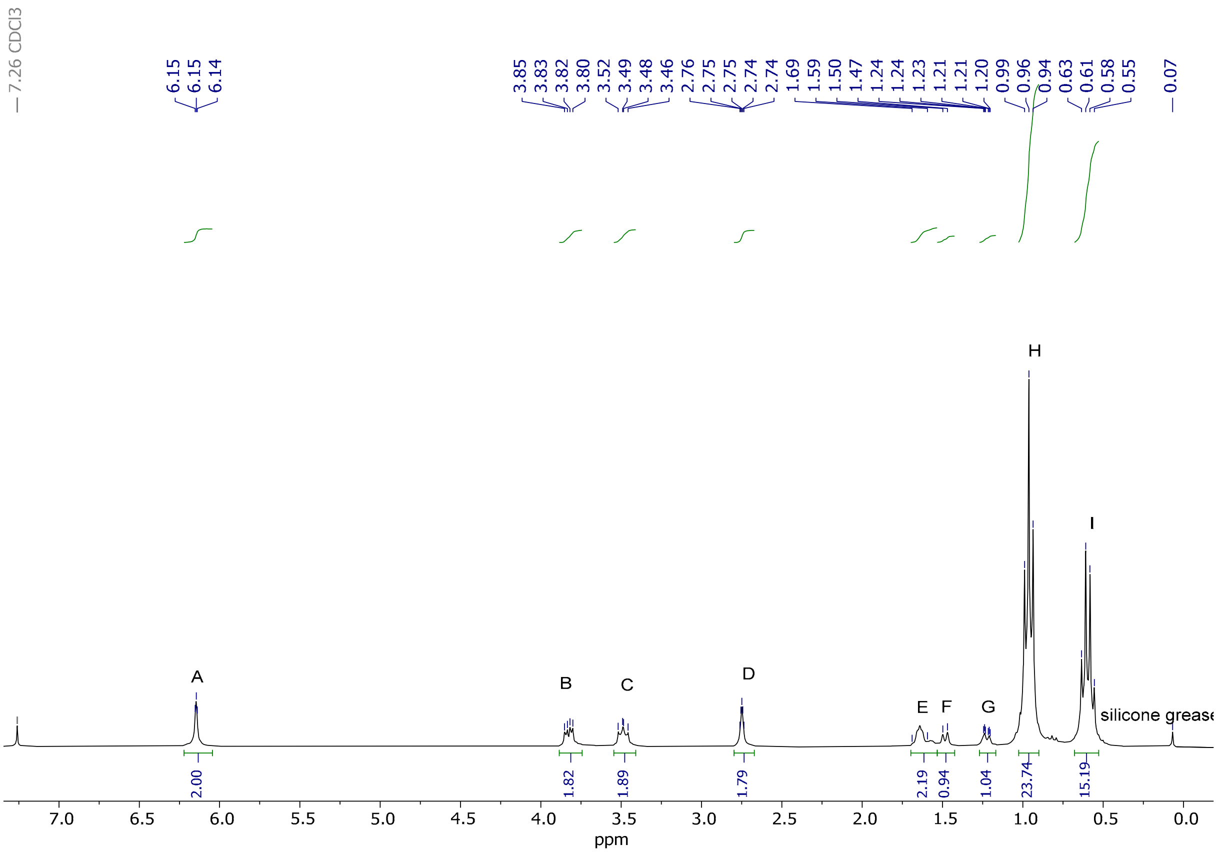

Figure S22. Compound **M8** – ^{1}H NMR in $CDCl_3$

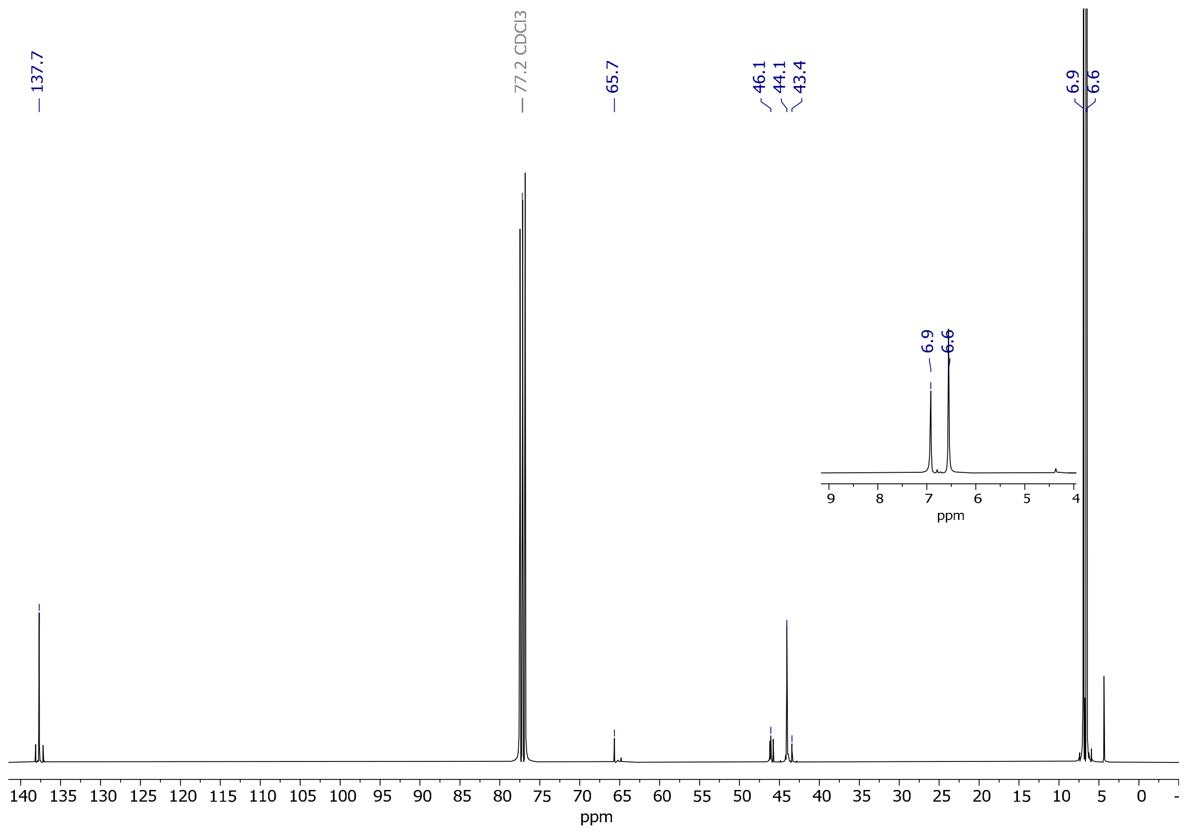

Figure S23. Compound **M8** – ^{13}C NMR in $CDCl_3$

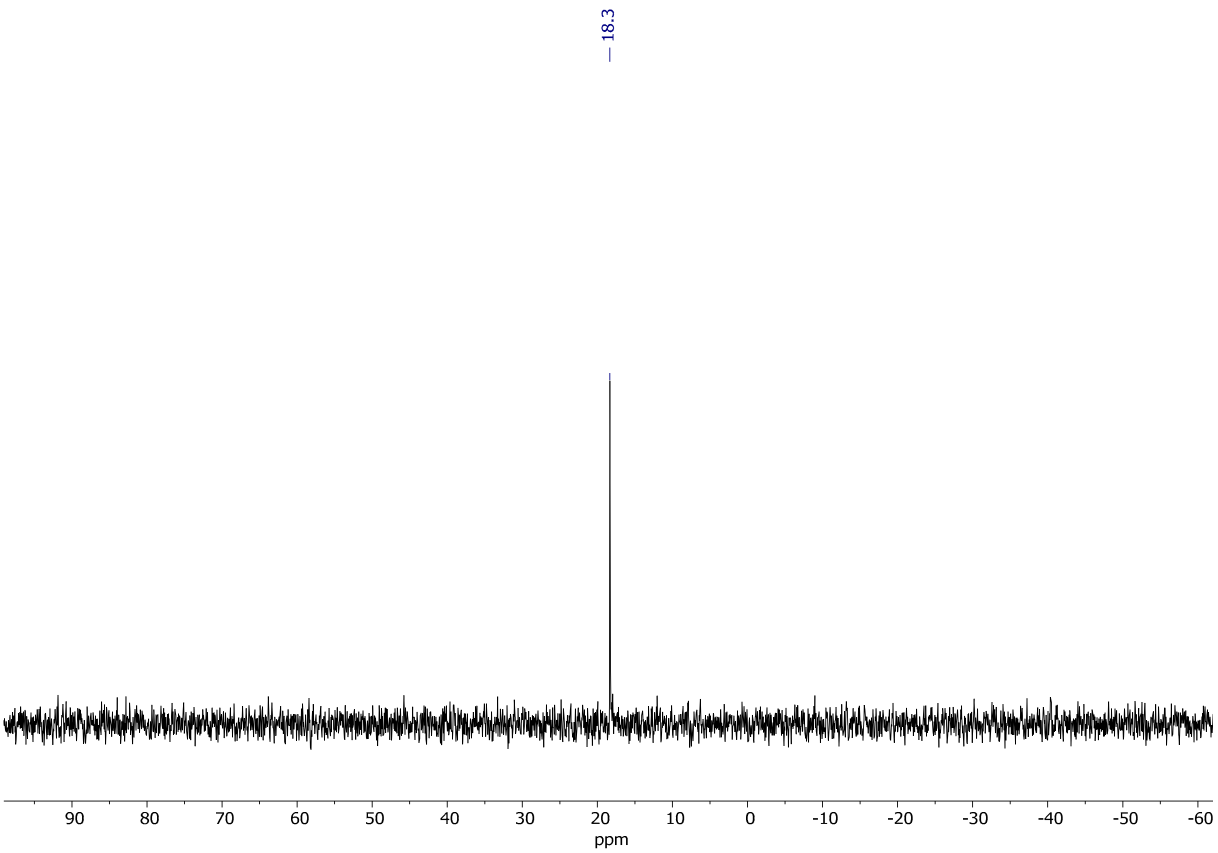

Figure S24. Compound **M8** – ^{29}Si NMR in $CDCl_3$

Compound **M10**

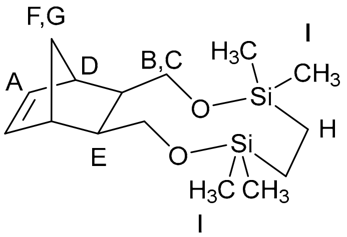

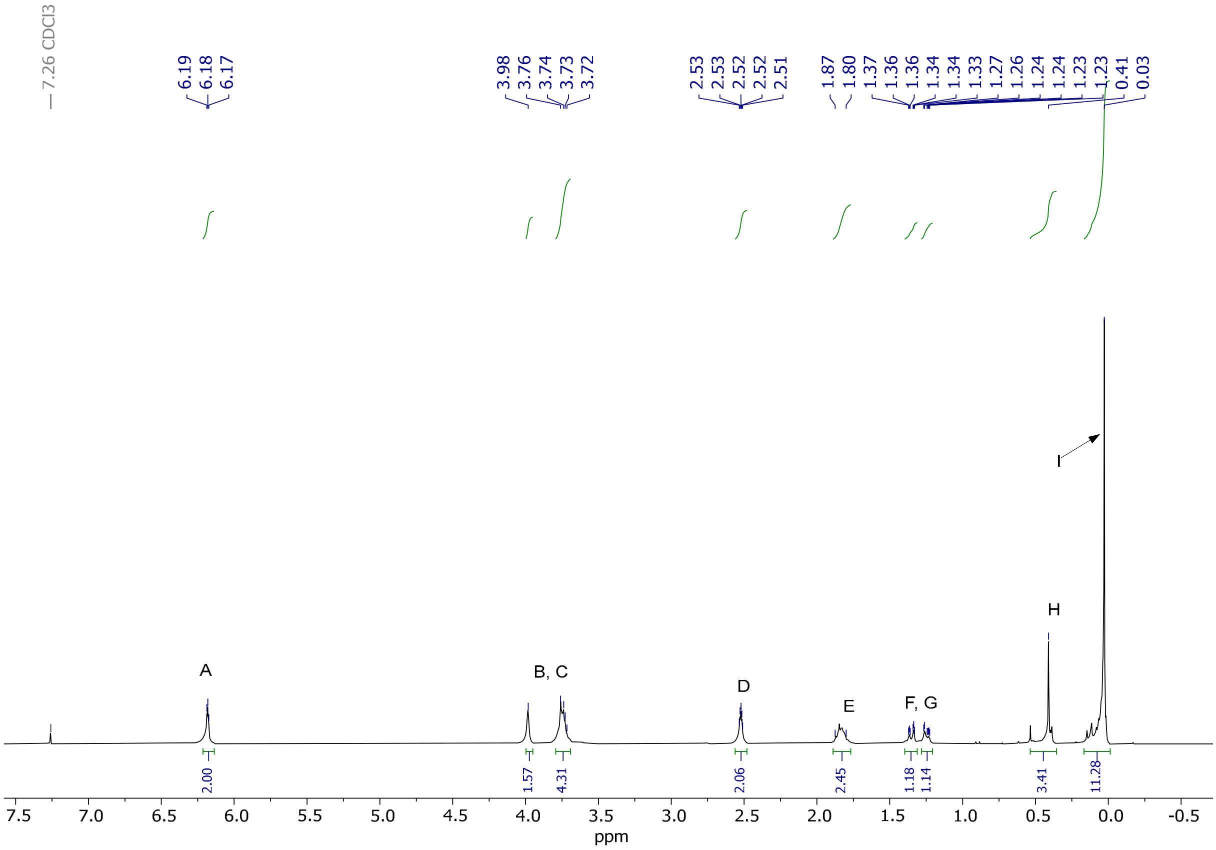

Figure S25. Compound **M10** – ^1H NMR in $CDCl_3$

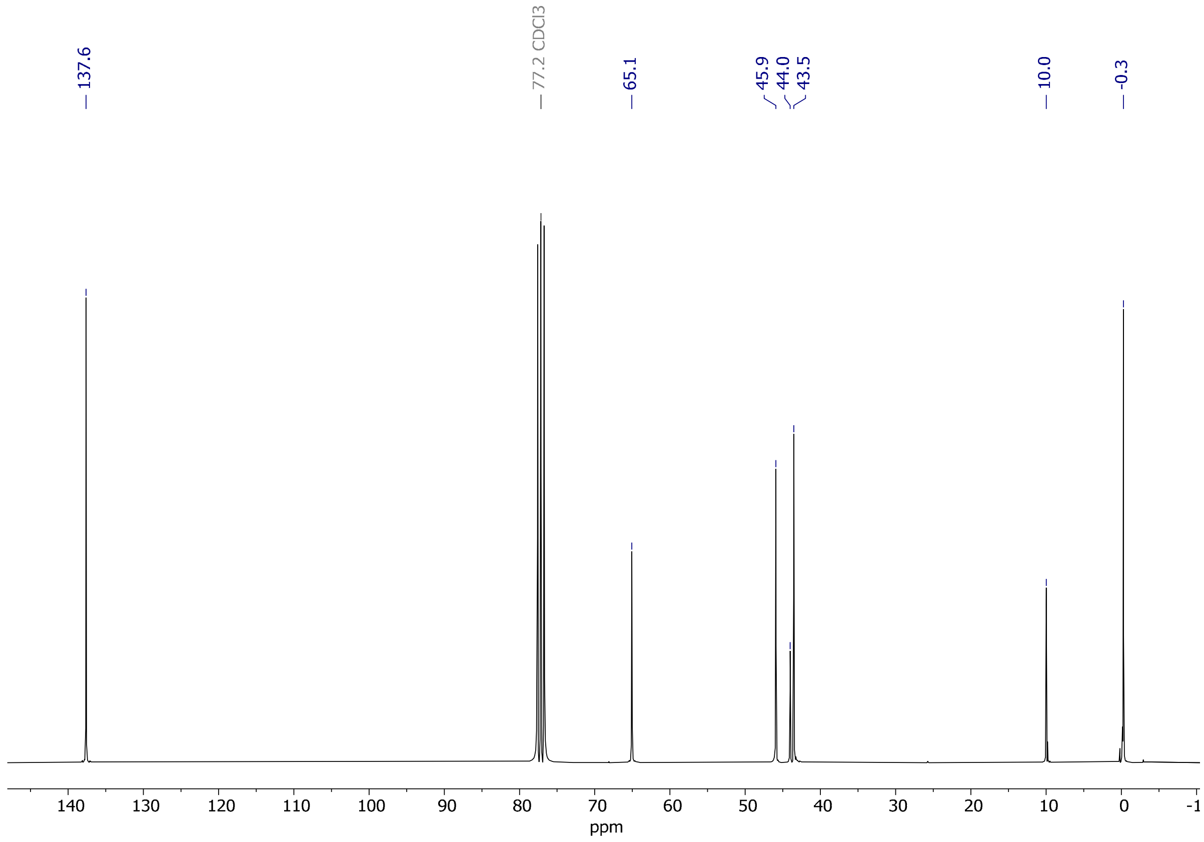

Figure S26. Compound **M10** – ^{13}C NMR in $CDCl_3$

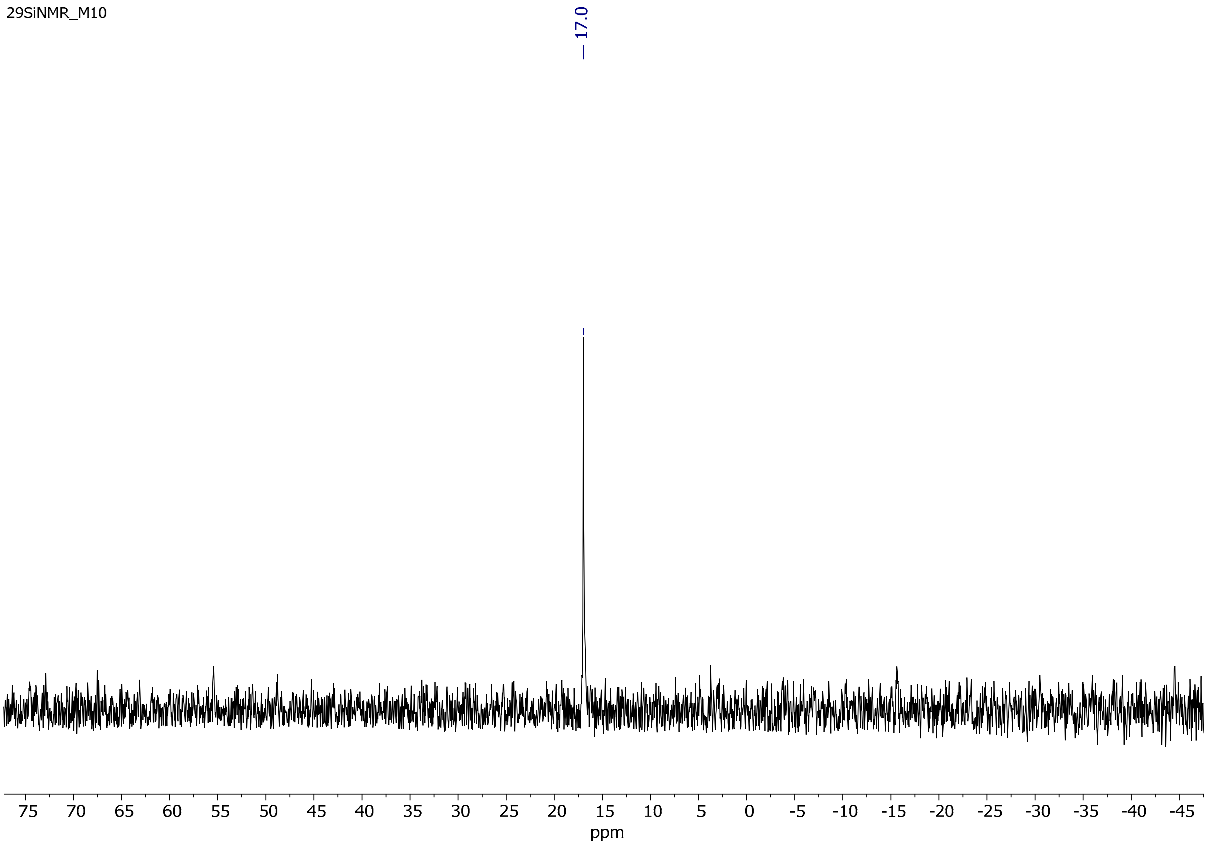

Figure S27. Compound **M10** – ^{29}Si NMR in $CDCl_3$

POLYMERS

Compound P1

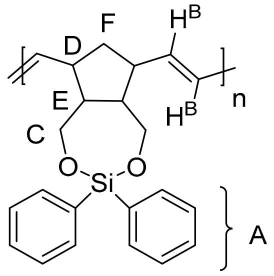

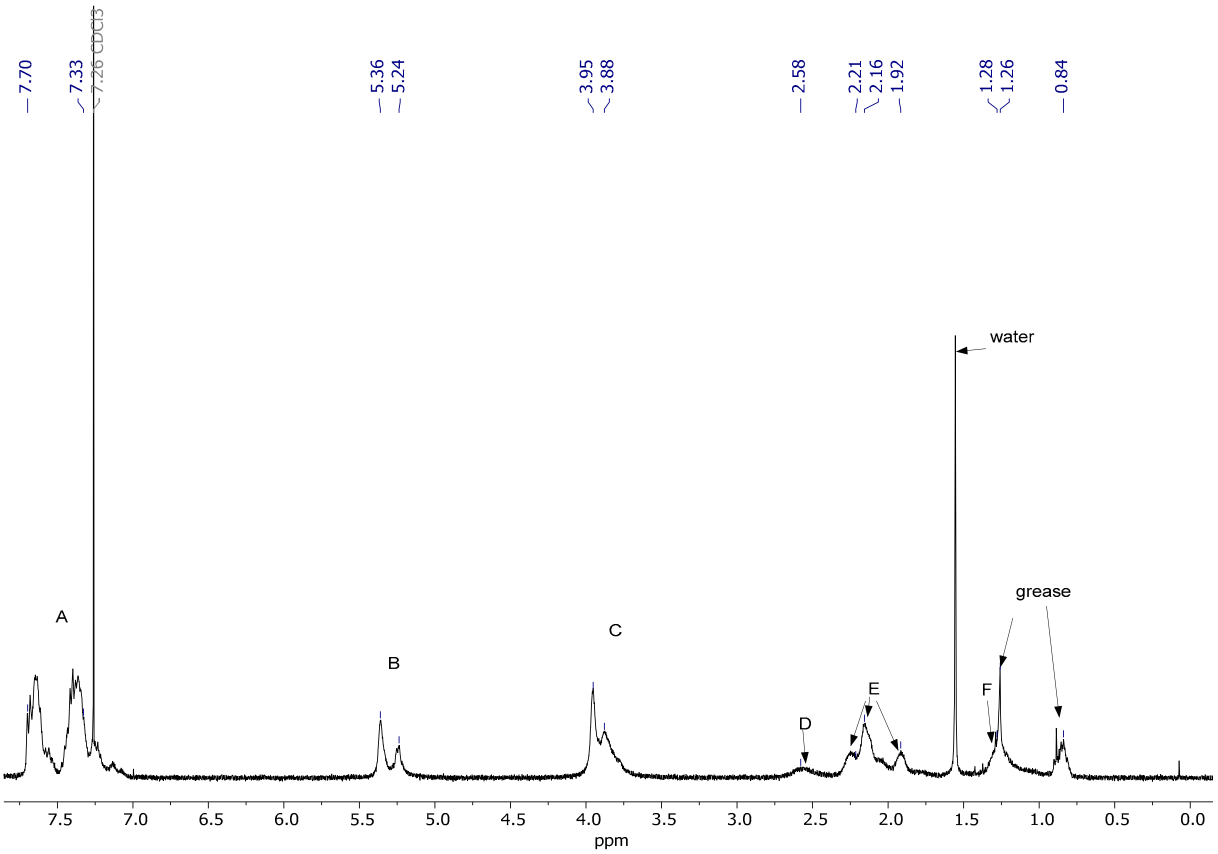

Figure S28. Compound **P1** – ^{1}H NMR in $CDCl_3$

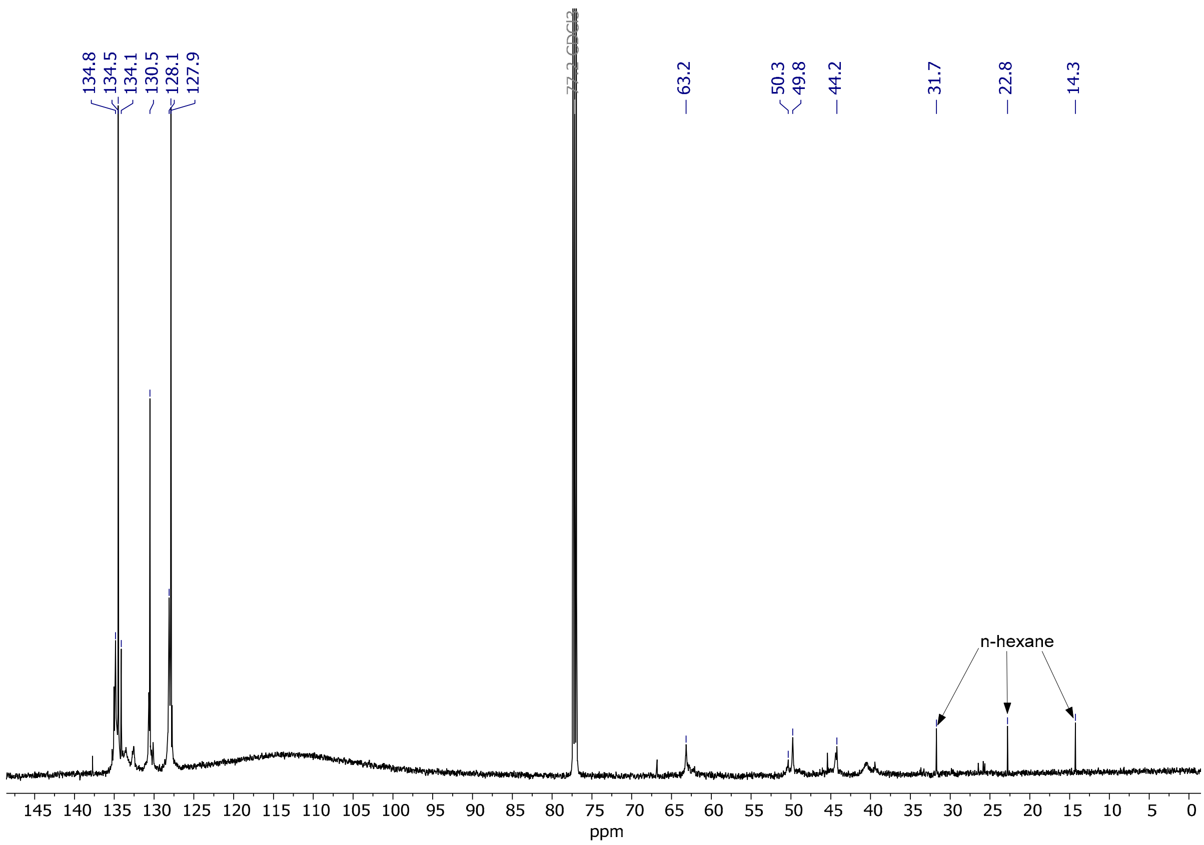

Figure S29. Compound **P1** – ^{13}C NMR in $CDCl_3$

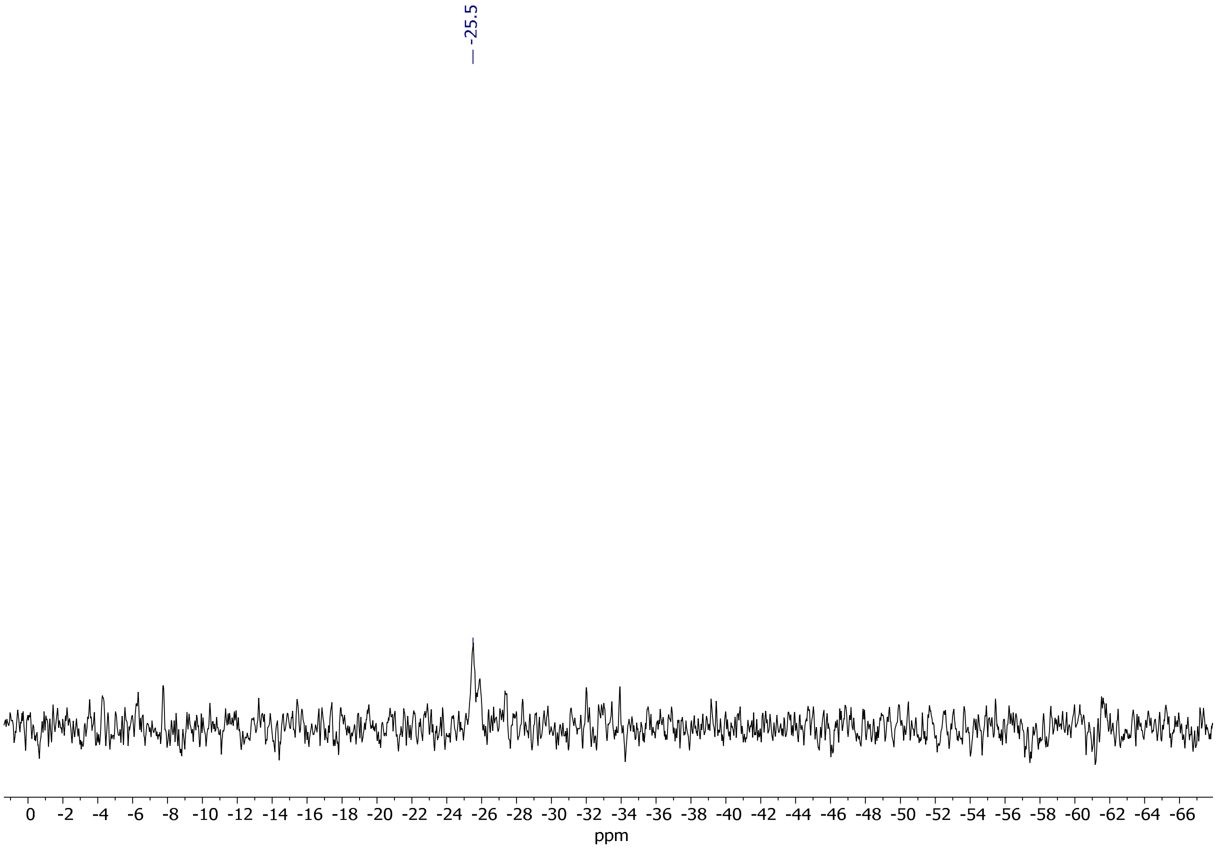

Figure S30. Compound **P1** – ^{29}Si NMR in $CDCl_3$

Compound P3

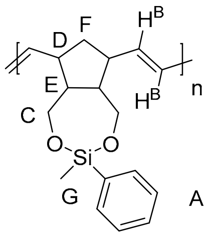

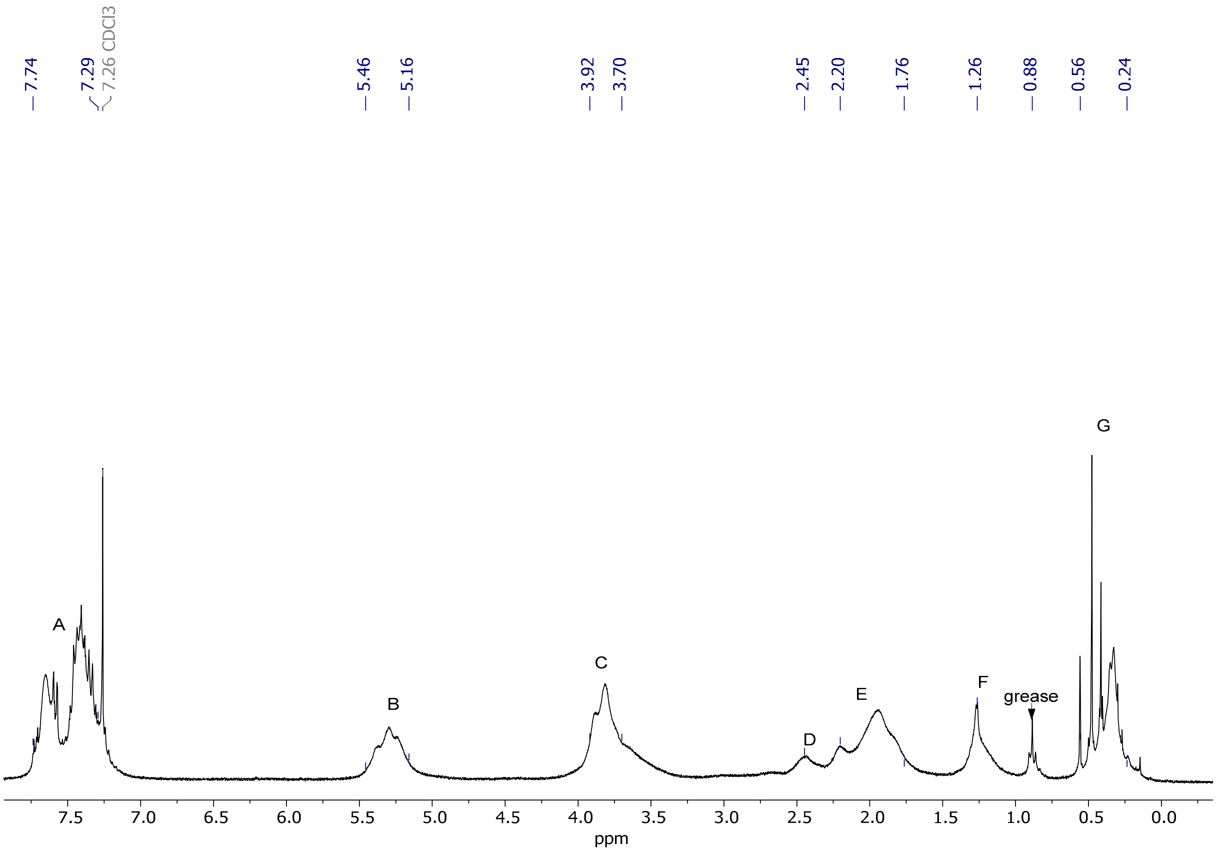

Figure S31. Compound **P3** – ^{1}H NMR in $CDCl_3$

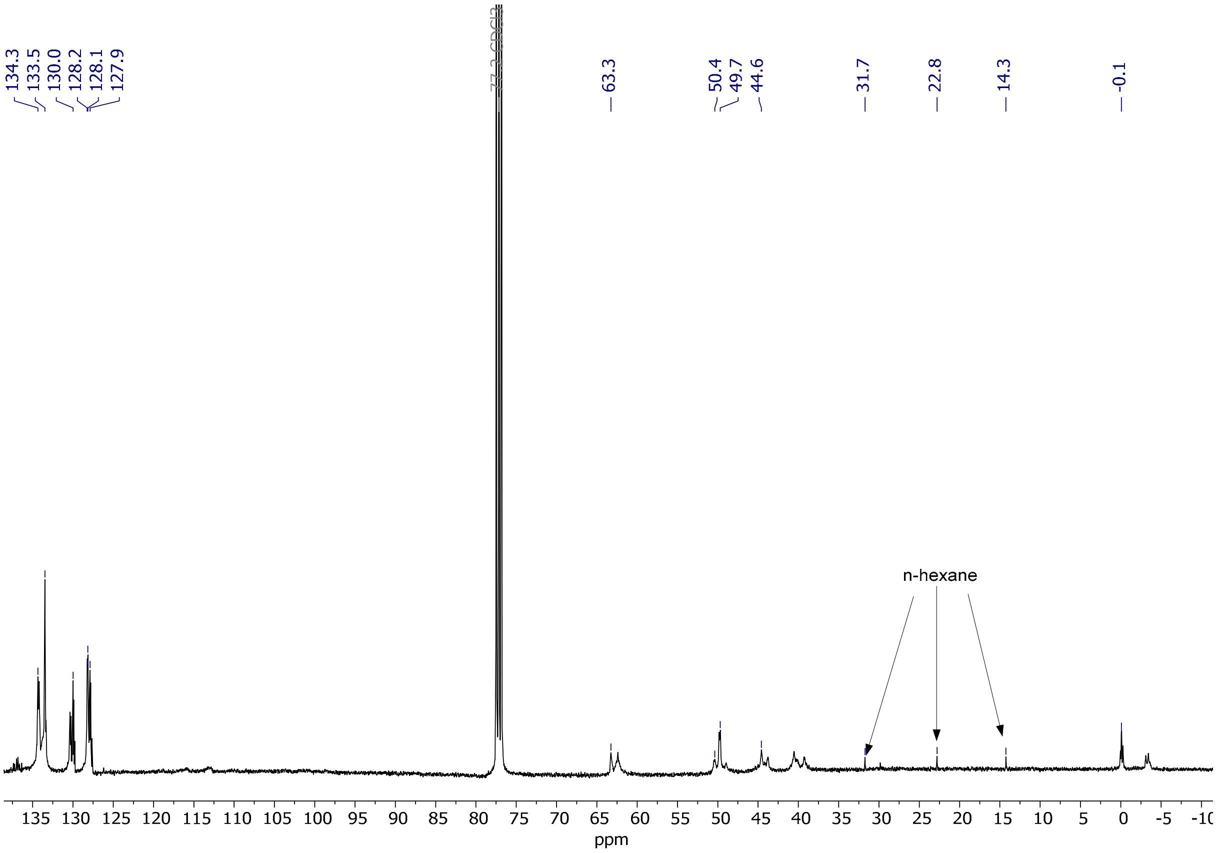

Figure S32. Compound **P3** – ^{13}C NMR in $CDCl_3$

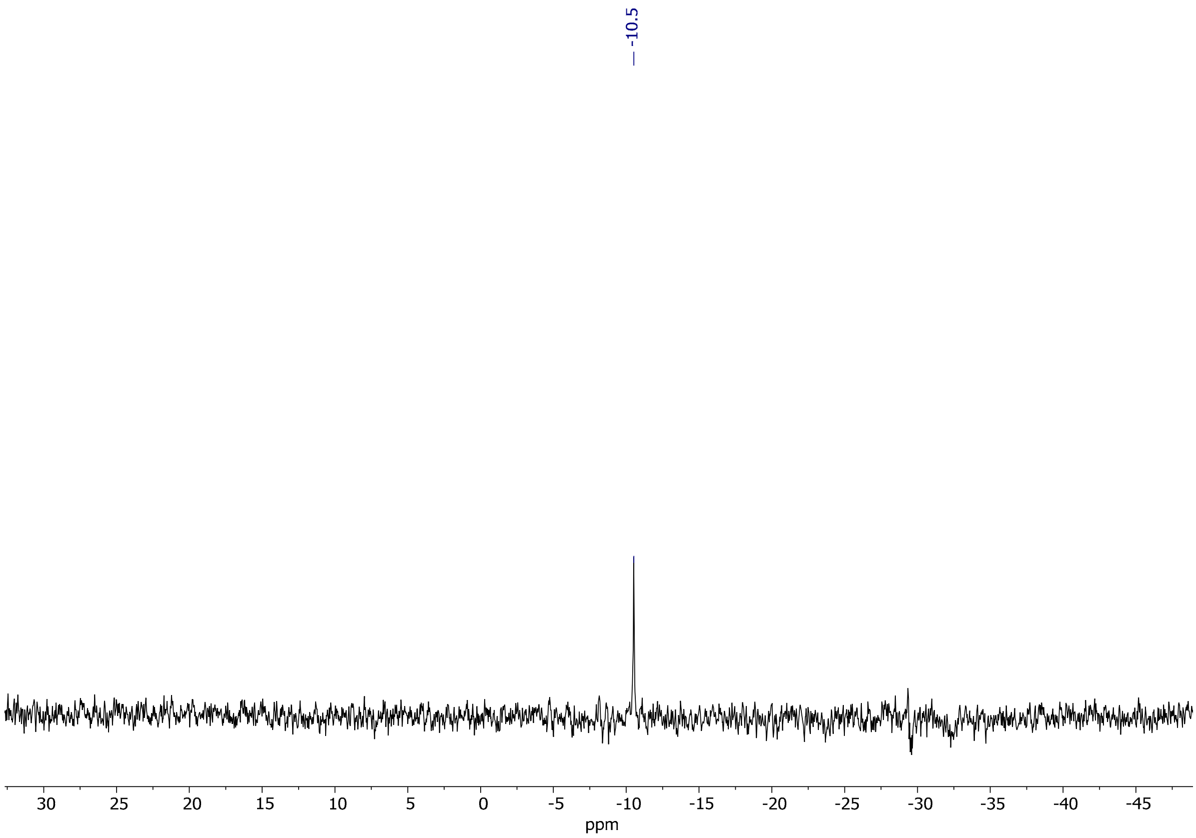

Figure S33. Compound **P3** – ^{29}Si NMR in $CDCl_3$

Compound P4

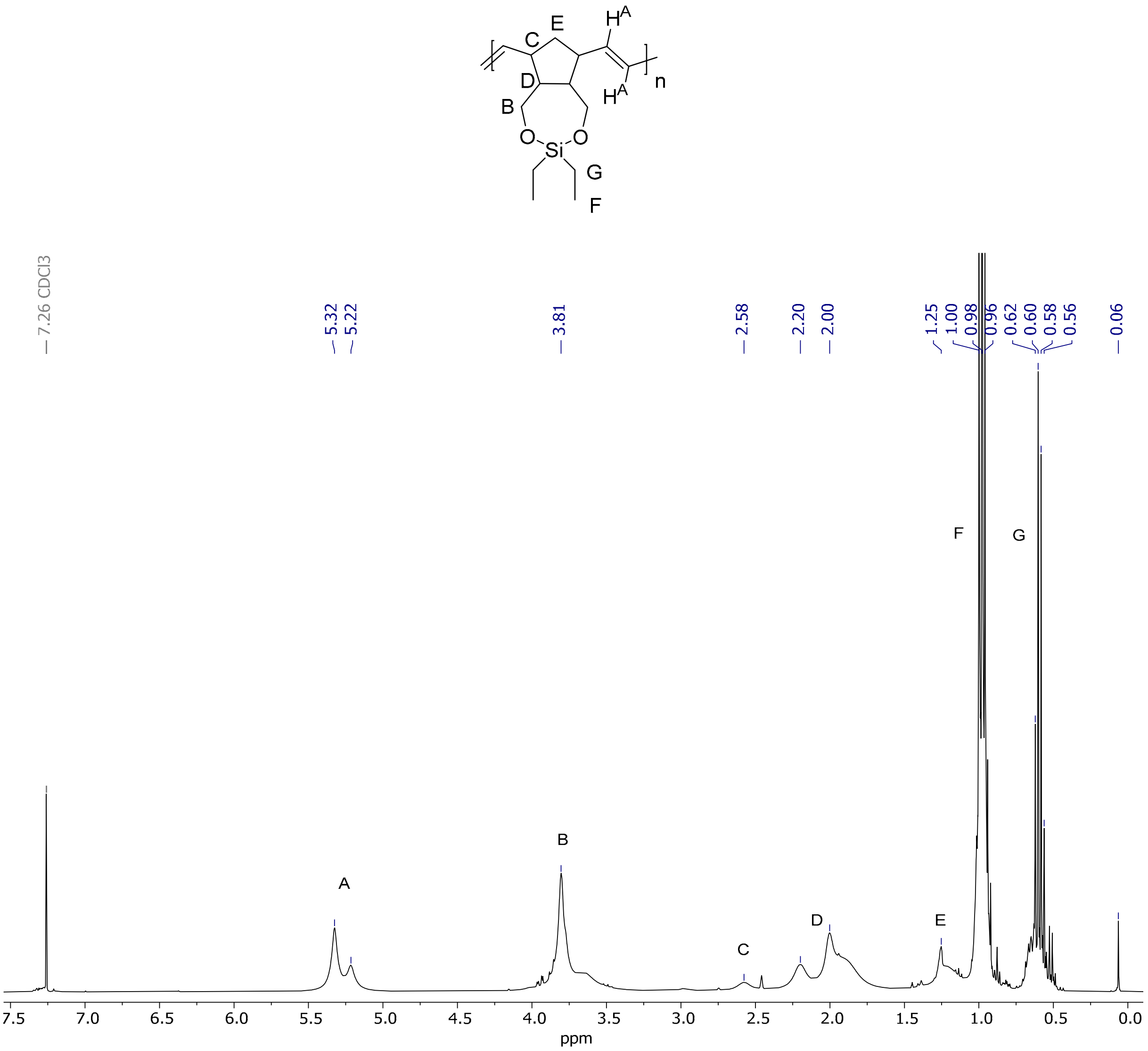

Figure S34. Compound **P4** – ^{1}H NMR in $CDCl_3$

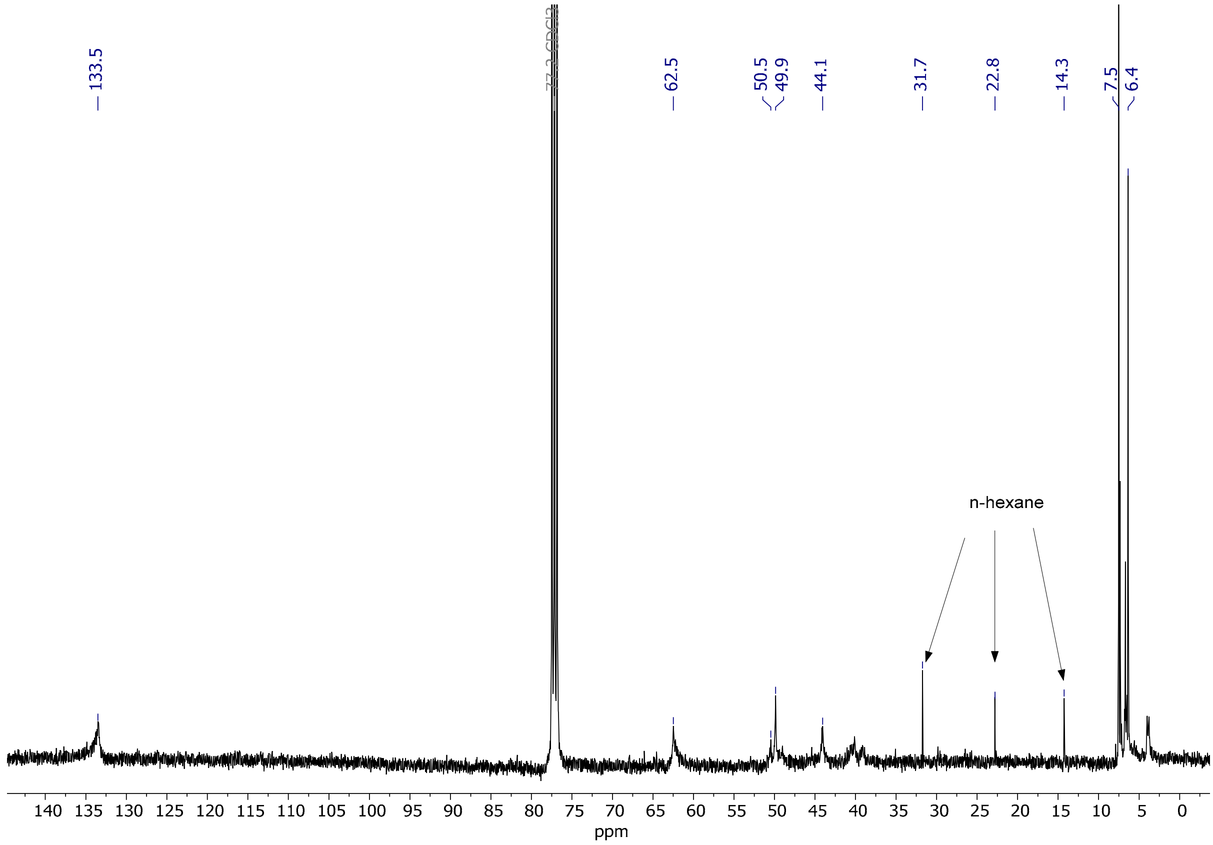

Figure S35. Compound **P4** – ^{13}C NMR in $CDCl_3$

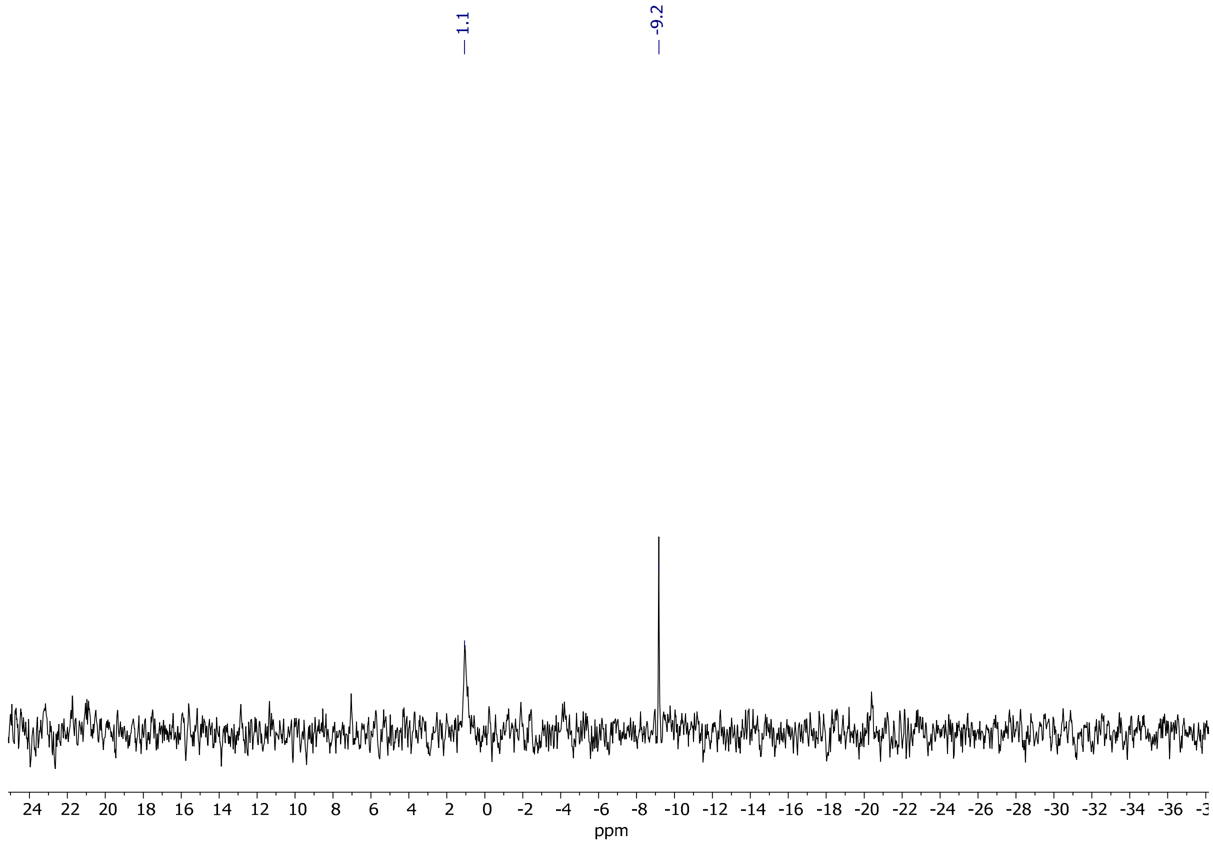

Figure S36. Compound **P4** – ^{29}Si NMR in $CDCl_3$

Compound P5

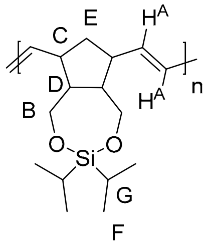

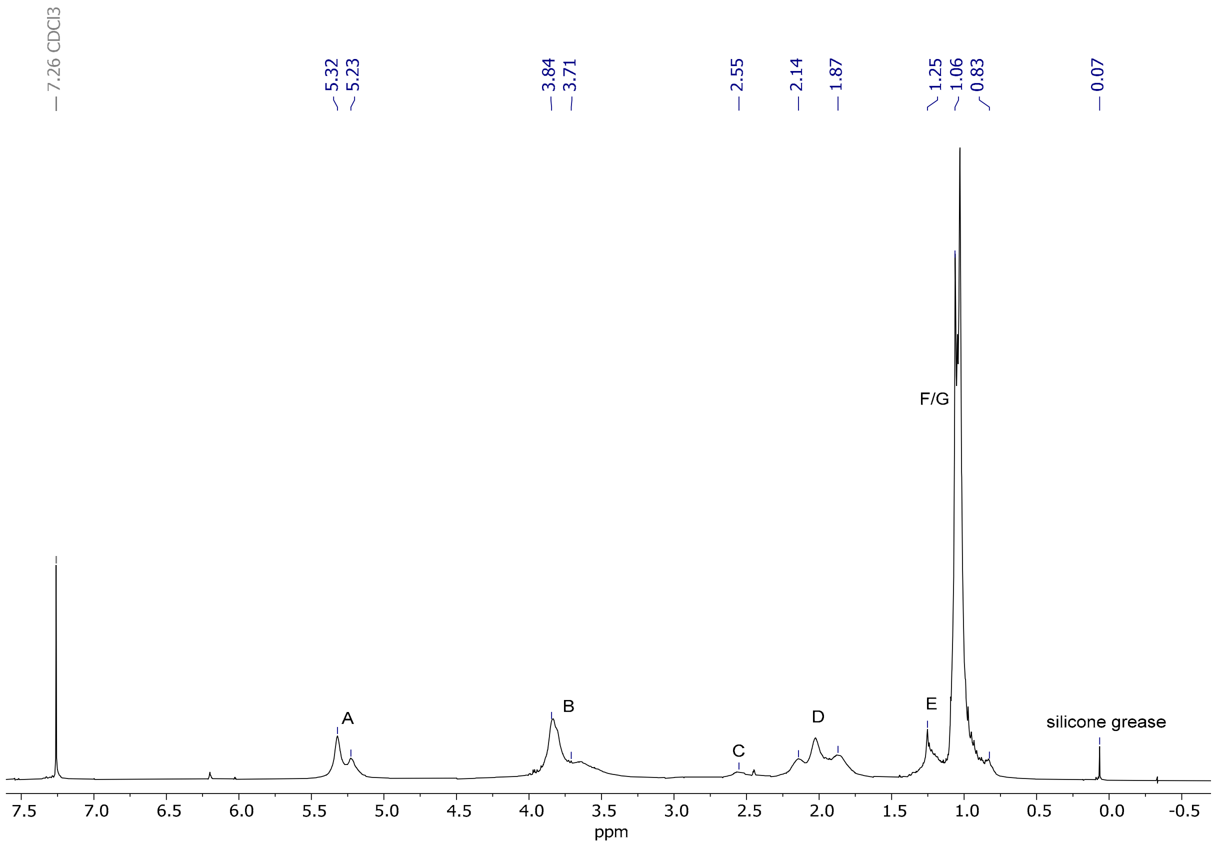

Figure S37. Compound **P5** – ^{1}H NMR in $CDCl_3$

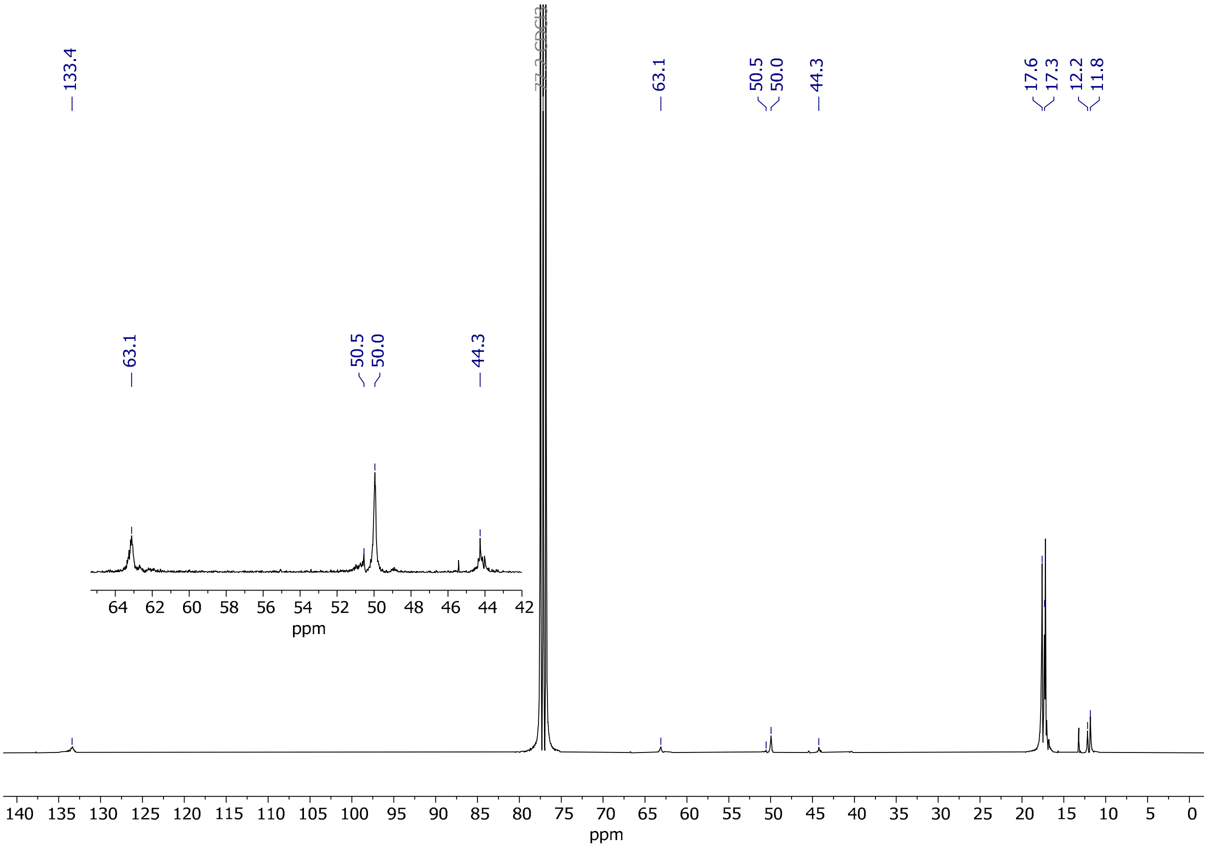

Figure S38. Compound **P5** – ^{13}C NMR in $CDCl_3$

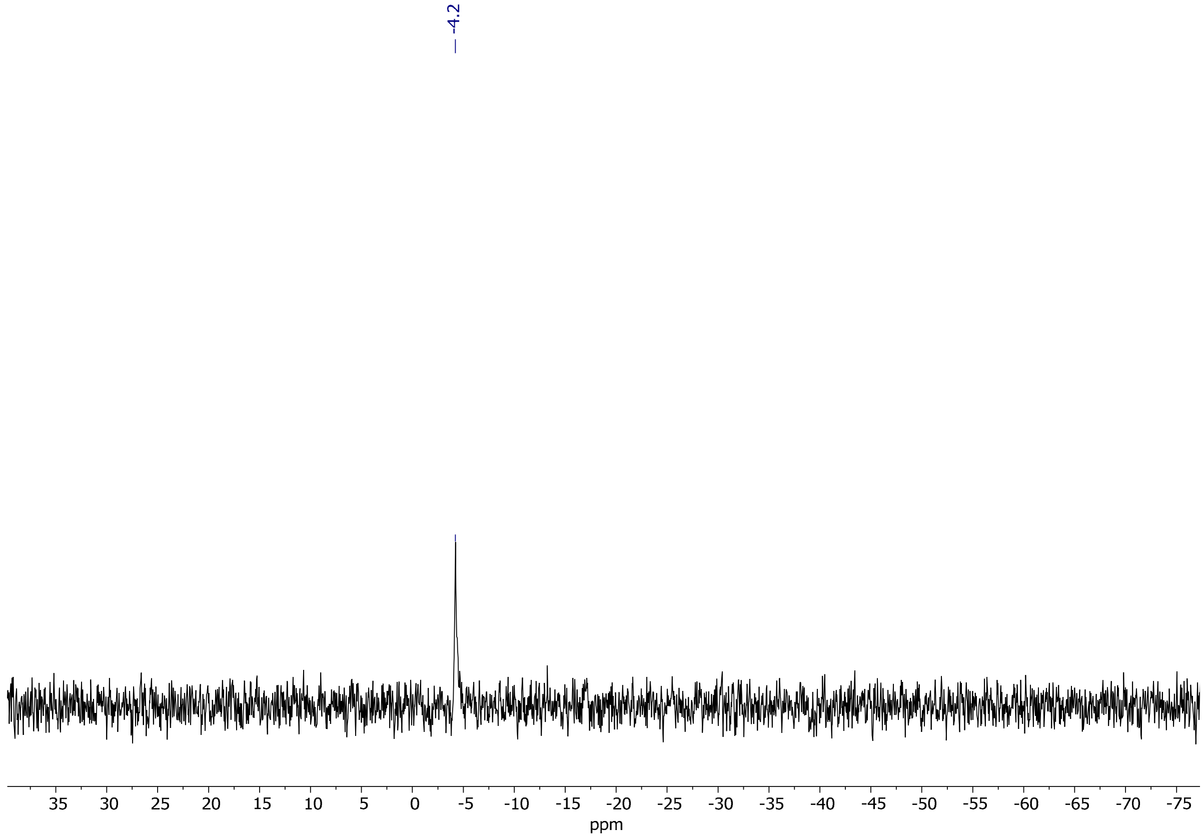

Figure S39. Compound **P5** – ^{29}Si NMR in $CDCl_3$

Compound P6

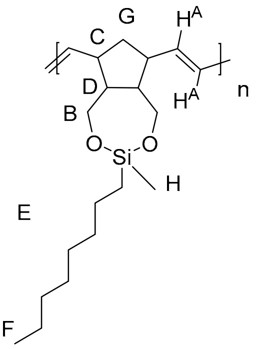

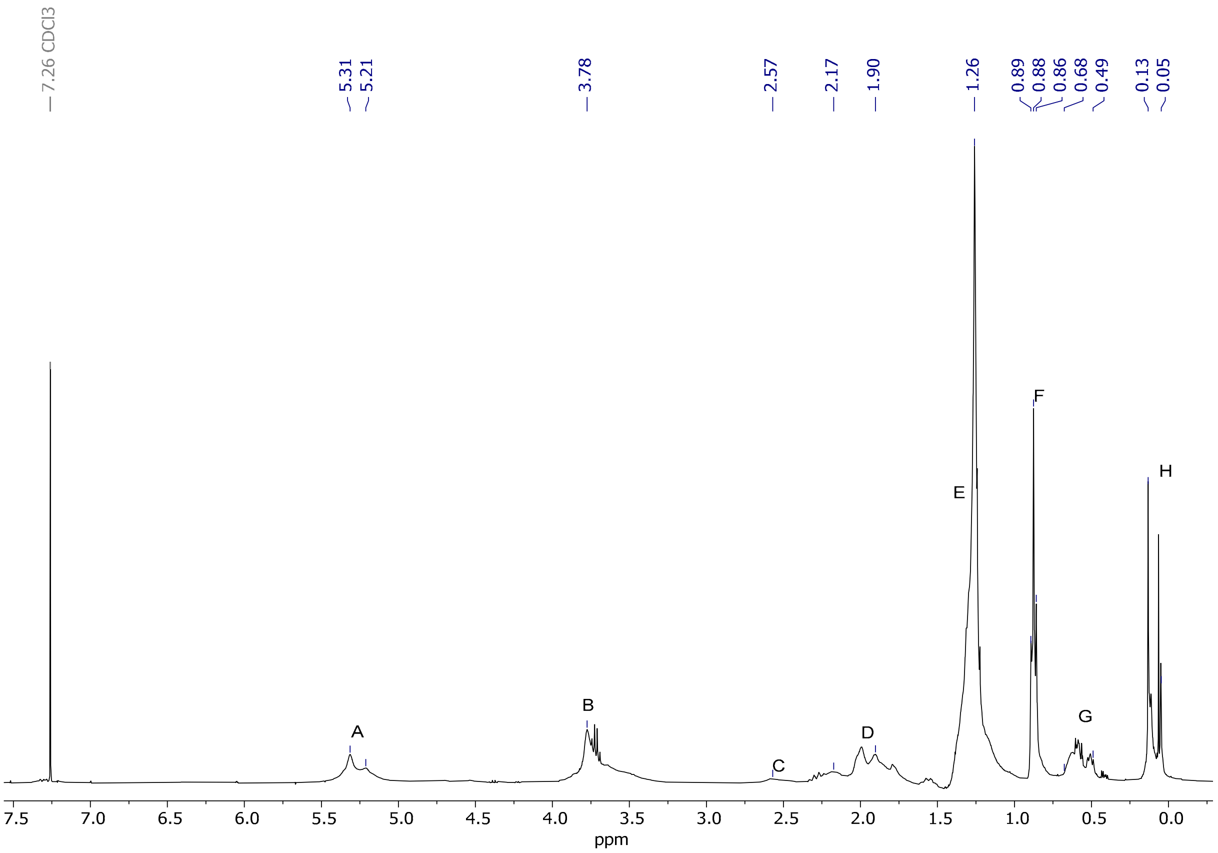

Figure S40. Compound **P6** – ^{1}H NMR in $CDCl_3$

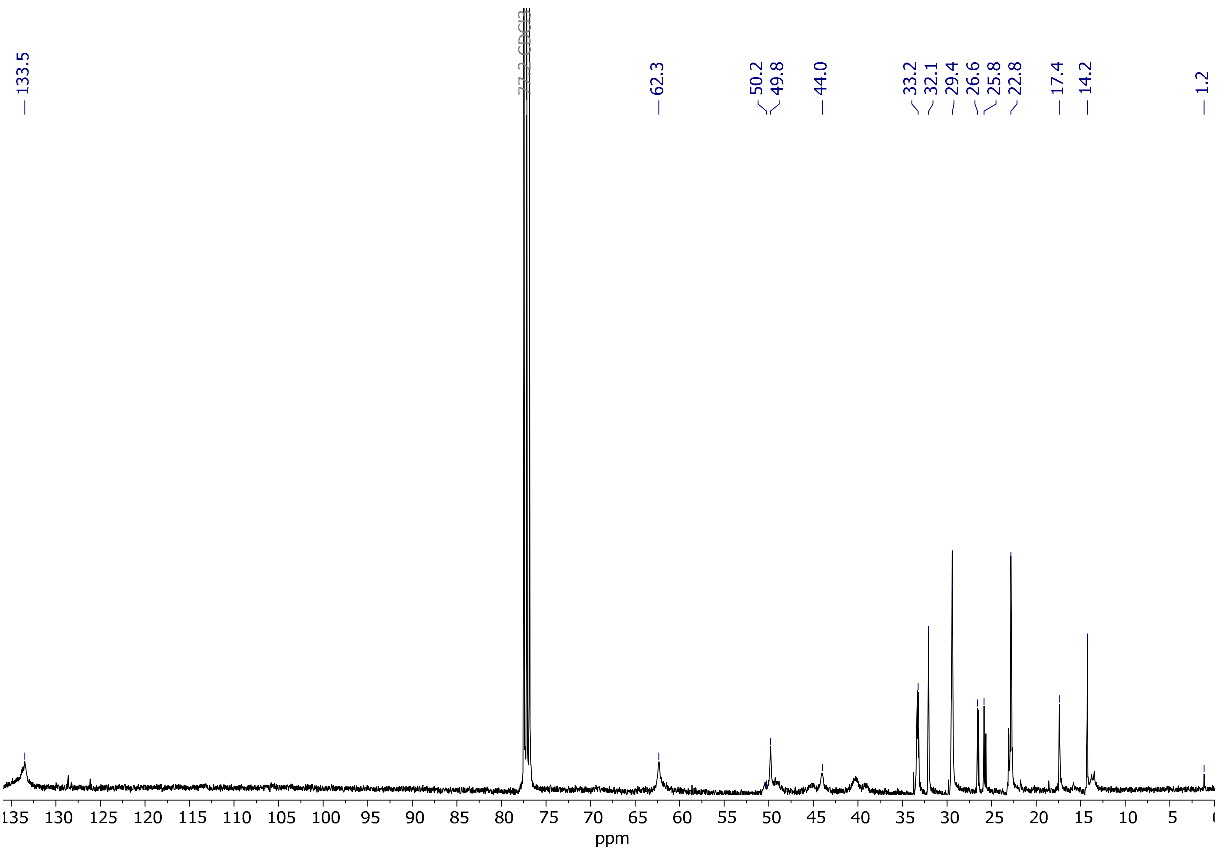

Figure S41. Compound **P6** – ^{13}C NMR in $CDCl_3$

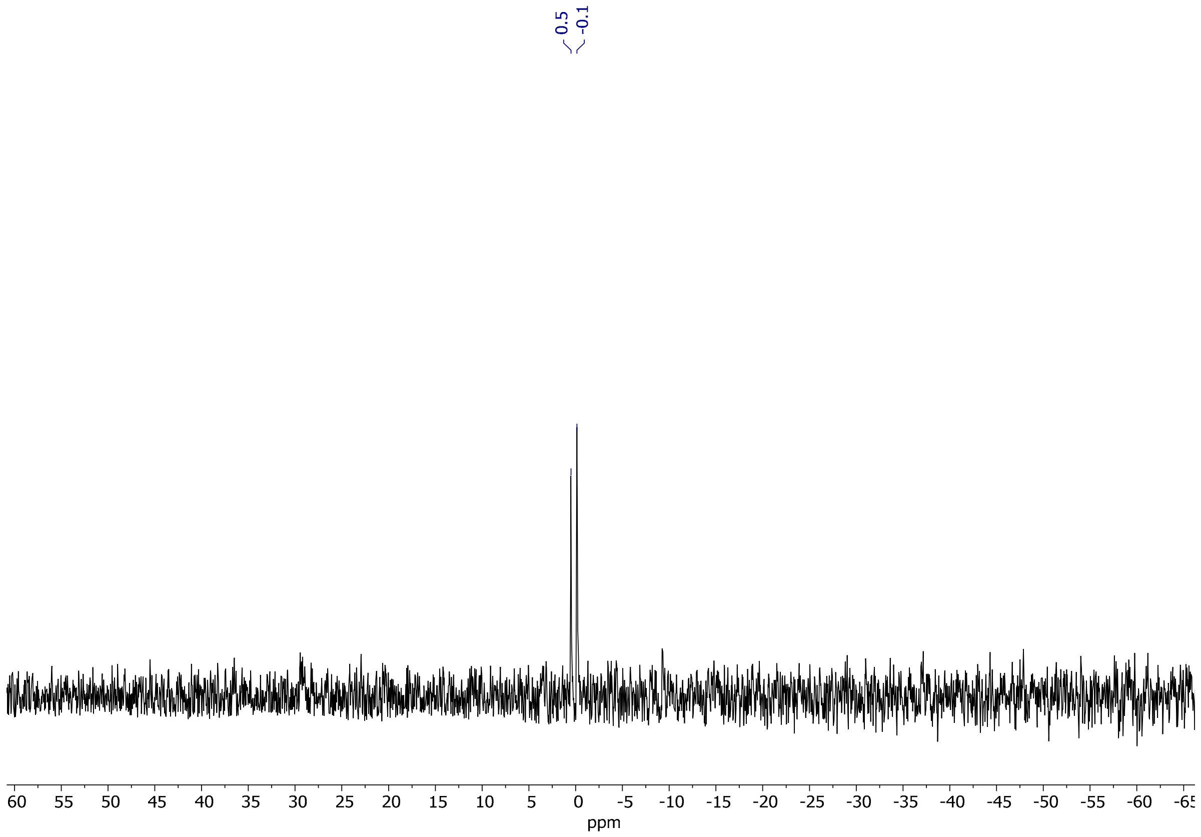

Figure S42. Compound **P6** – ^{29}Si NMR in $CDCl_3$

Compound P7

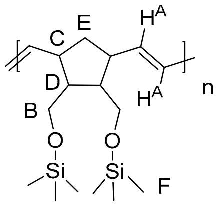

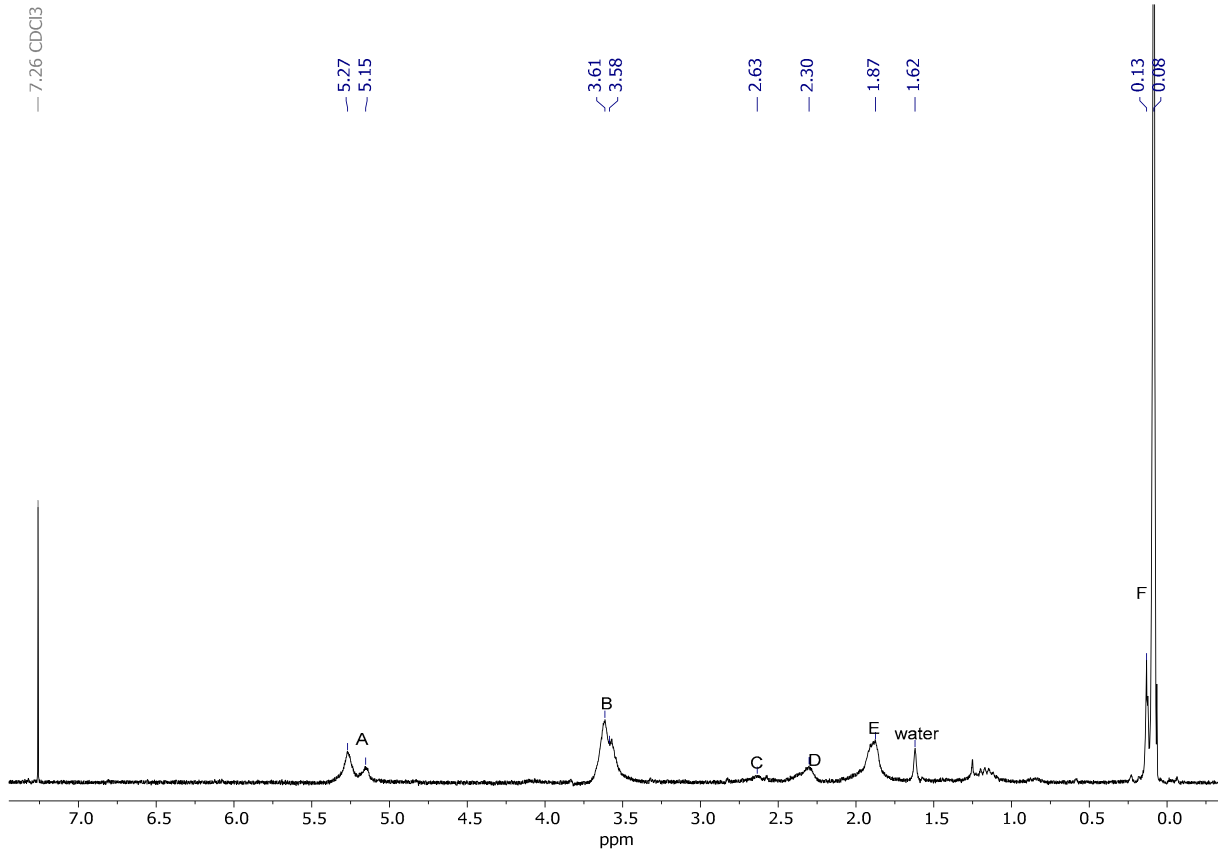

Figure S43. Compound **P7** – ^{1}H NMR in $CDCl_3$

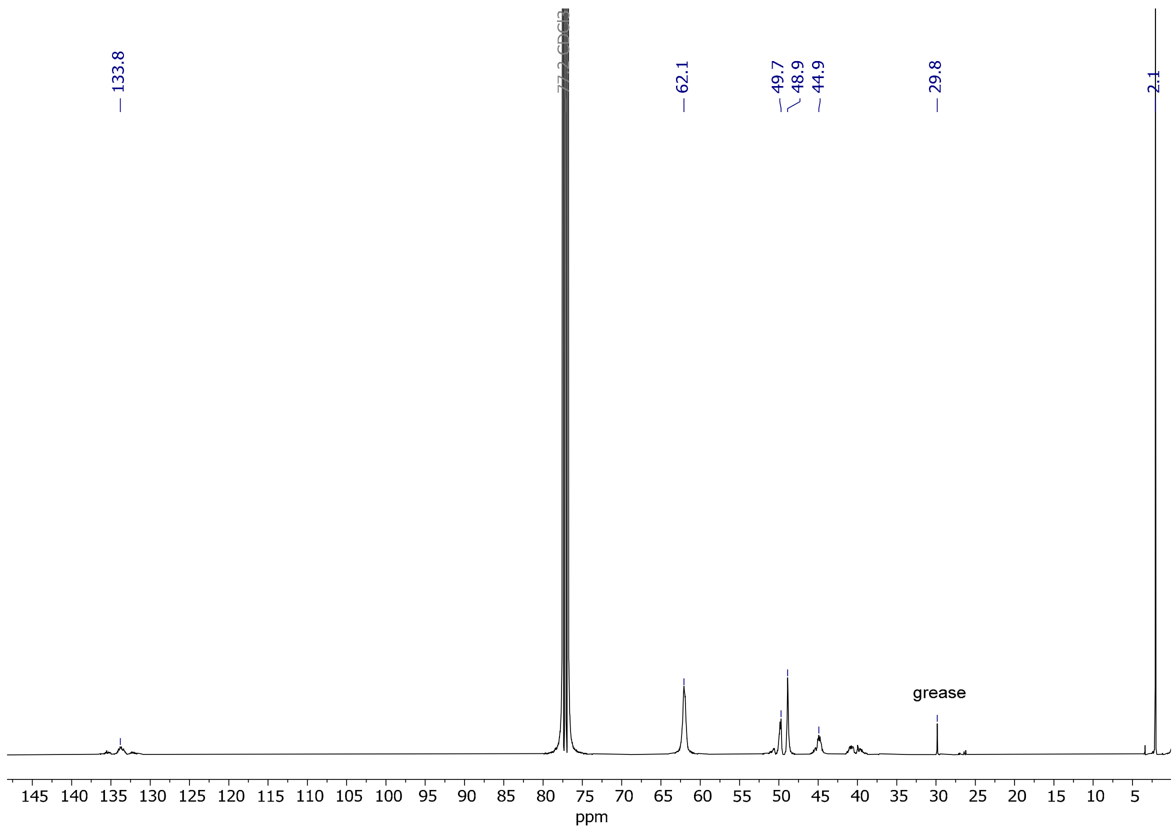

Figure S44. Compound **P7** – ^{13}C NMR in $CDCl_3$

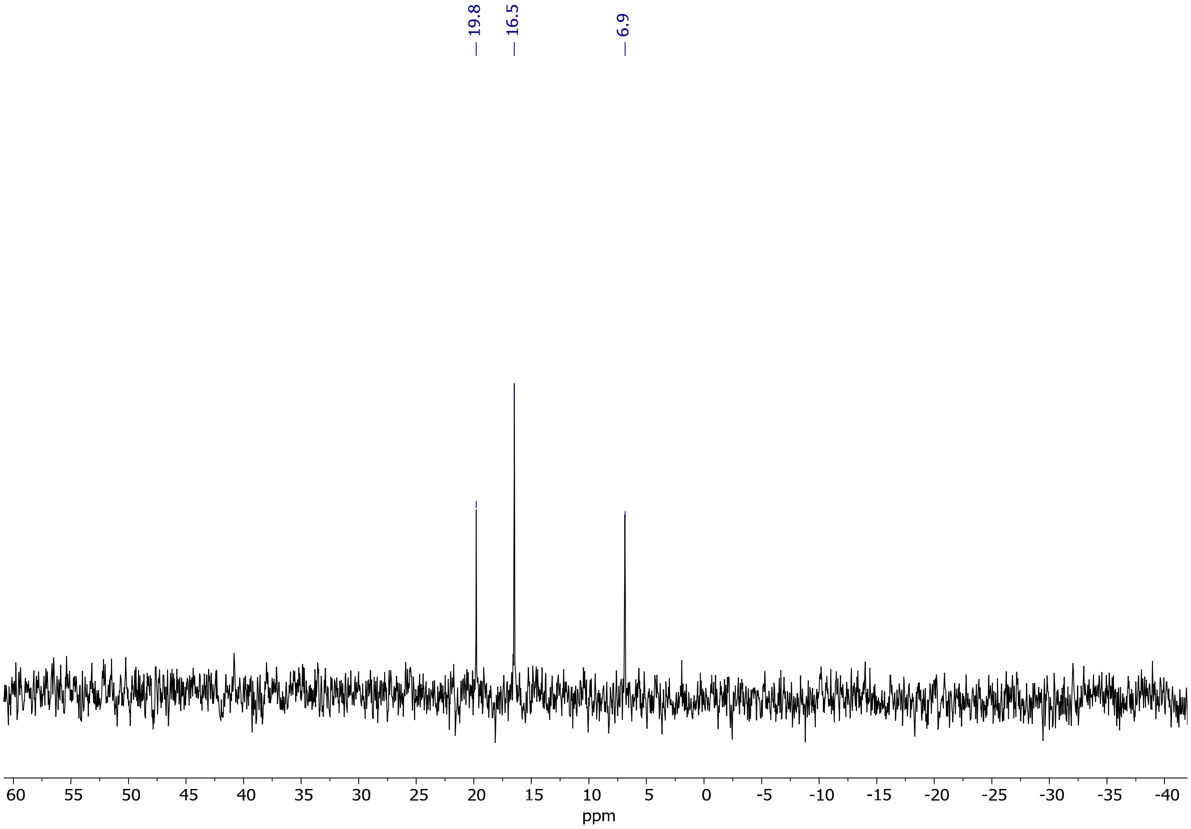

Figure S45. Compound **P7** – ^{29}Si NMR in $CDCl_3$

Compound P8

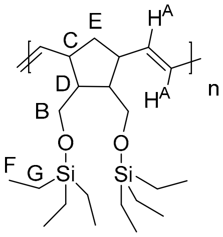

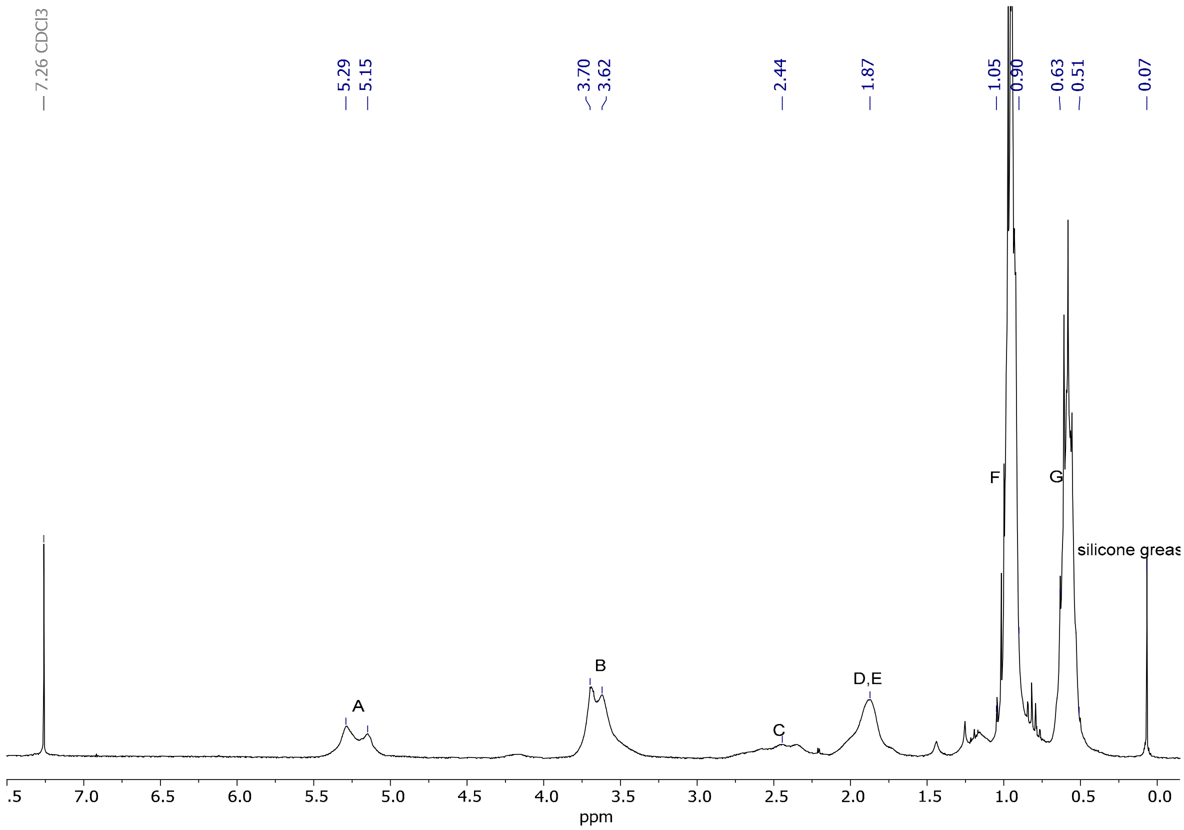

Figure S46. Compound **P8** – ^{1}H NMR in $CDCl_3$

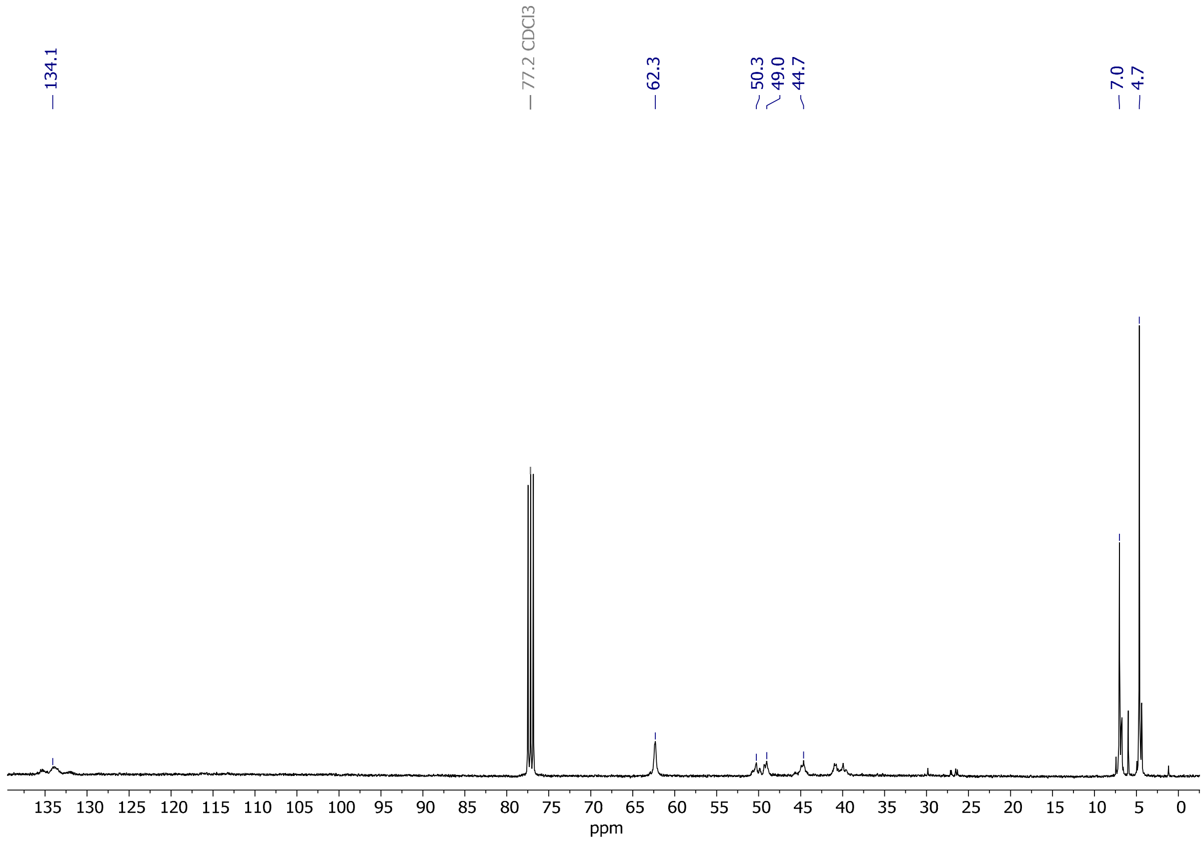

Figure S47. Compound **P8** – ^{13}C NMR in $CDCl_3$

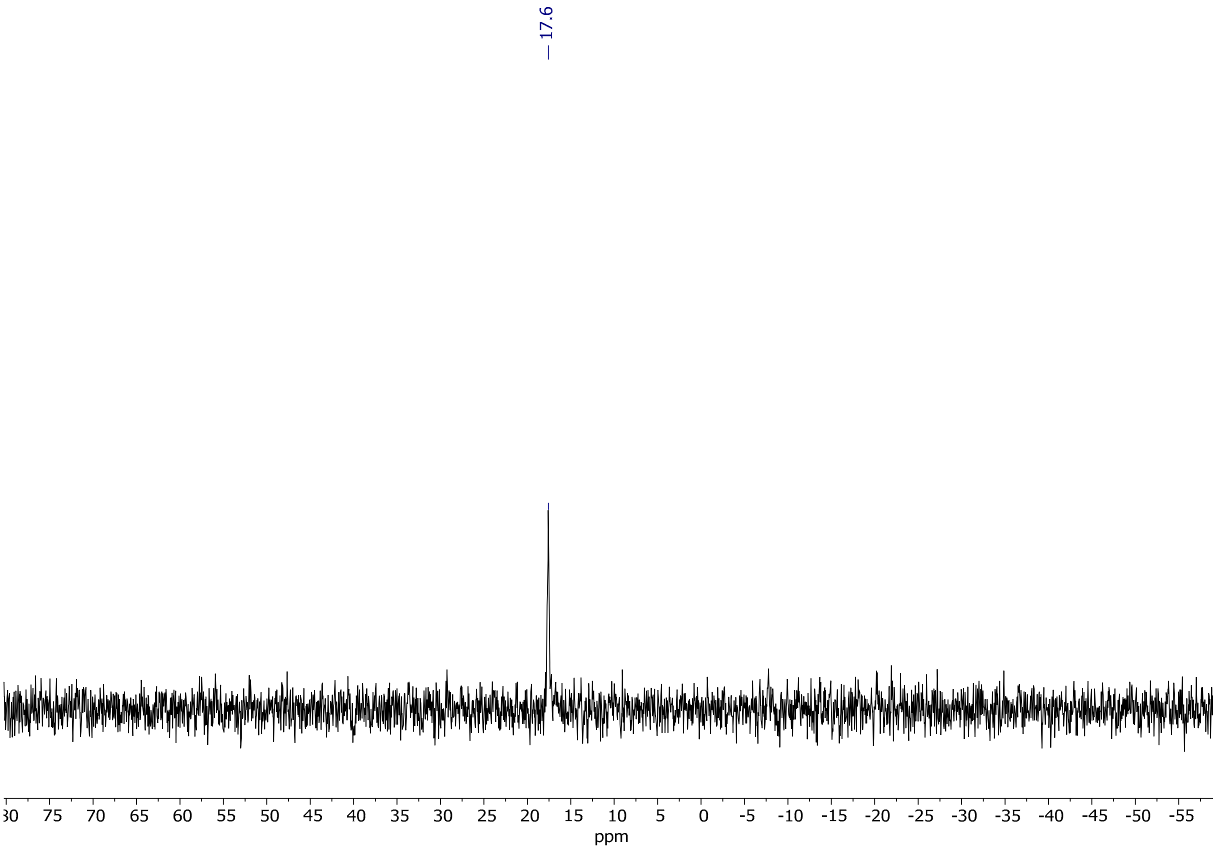

Figure S48. Compound **P8** – ^{29}Si NMR in $CDCl_3$

GPC Analysis

Results

Analysed by admin at 1:32:31 PM on Friday, November 03, 2023

Comments

Molecular Weight Averages

Peak	Mp	Mn	Mw	Mz	Mz+1	Mv	PD
Peak 1	100908	57288	116420	218115	337841	201572	2.032
Peak 2	956	957	1015	1075	1135	1066	1.061

Peak information

	Start (mins)	End (mins)
Baseline region 1	0.71	5.73
Baseline region 2	15.67	15.84
Peak 1	7.08	10.35
Peak 2	11.42	11.86

Peak	Trace	Peak Max RT (mins)	Peak Area (mV.s)	Peak Height (mV)
Peak 1	RI	8.48	20250.624	186.062
Peak 1	UV 250nm	7.68	12096.611	-217.222
Peak 2	RI	11.67	830.182	42.146
Peak 2	UV 250nm	11.83	2269.374	156.318

Chromatogram

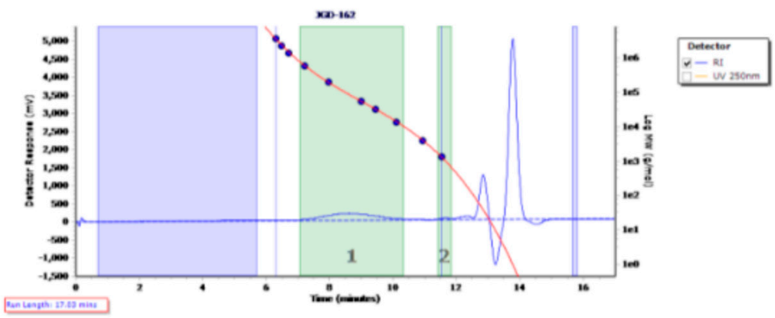

Figure S49. Compound **P1** – GPC measurement

Results

Analysed by admin at 1:34:42 PM on Friday, November 03, 2023

Comments

Molecular Weight Averages

Peak	Mp	Mn	Mw	Mz	Mz+1	Mv	PD
Peak 1	44194	39964	45311	51200	58219	50253	1.134
Peak 2	855	813	888	966	1043	955	1.092

Peak information

	Start (mins)	End (mins)
Baseline region 1	3.04	5.83
Baseline region 2	15.34	15.46
Peak 1	8.17	10.18
Peak 2	11.31	12.00

Peak	Trace	Peak Max RT (mins)	Peak Area (mV.s)	Peak Height (mV)
Peak 1	RI	9.18	34366.047	886.958
Peak 1	UV 250nm	9.64	2802.739	-123.892
Peak 2	RI	11.72	1060.326	44.300
Peak 2	UV 250nm	11.90	3701.246	-172.795

Chromatogram

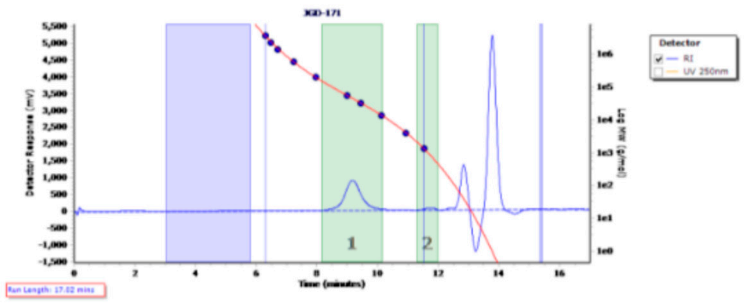

Figure S50. Compound **P7** – GPC measurement

Results

Analysed by admin at 1:33:31 PM on Friday, November 03, 2023
Comments

Molecular Weight Averages

Peak	Mp	Mn	Mw	Mz	Mz+1	Mv	PD
Peak 1	42088	36036	41108	45833	50689	45135	1.141
Peak 2	546	590	804	1102	1426	1055	1.363

Peak information

	Start (mins)	End (mins)
Baseline region 1	4.14	6.93
Baseline region 2	16.10	16.26
Peak 1	8.17	10.52
Peak 2	11.16	12.22

Peak	Trace	Peak Max RT (mins)	Peak Area (mV.s)	Peak Height (mV)
Peak 1	RI	9.23	69969.391	1784.191
Peak 1	UV 250nm	9.08	9481.656	176.693
Peak 2	RI	11.92	4670.980	126.258
Peak 2	UV 250nm	11.26	3698.474	118.179

Chromatogram

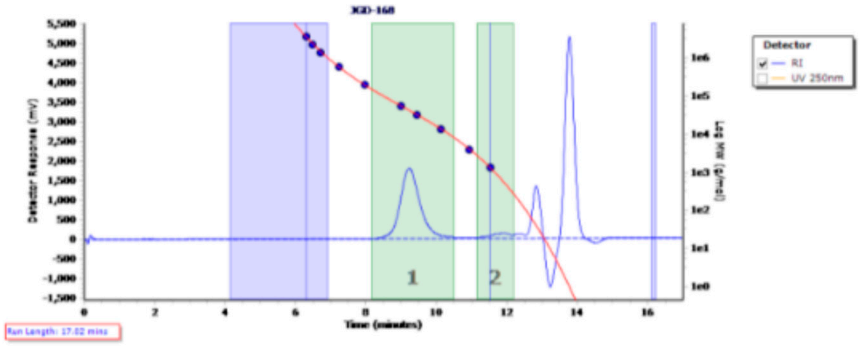

Figure S51. Compound **P8** – GPC measurement

TGA analysis (thermogravimetric curves)

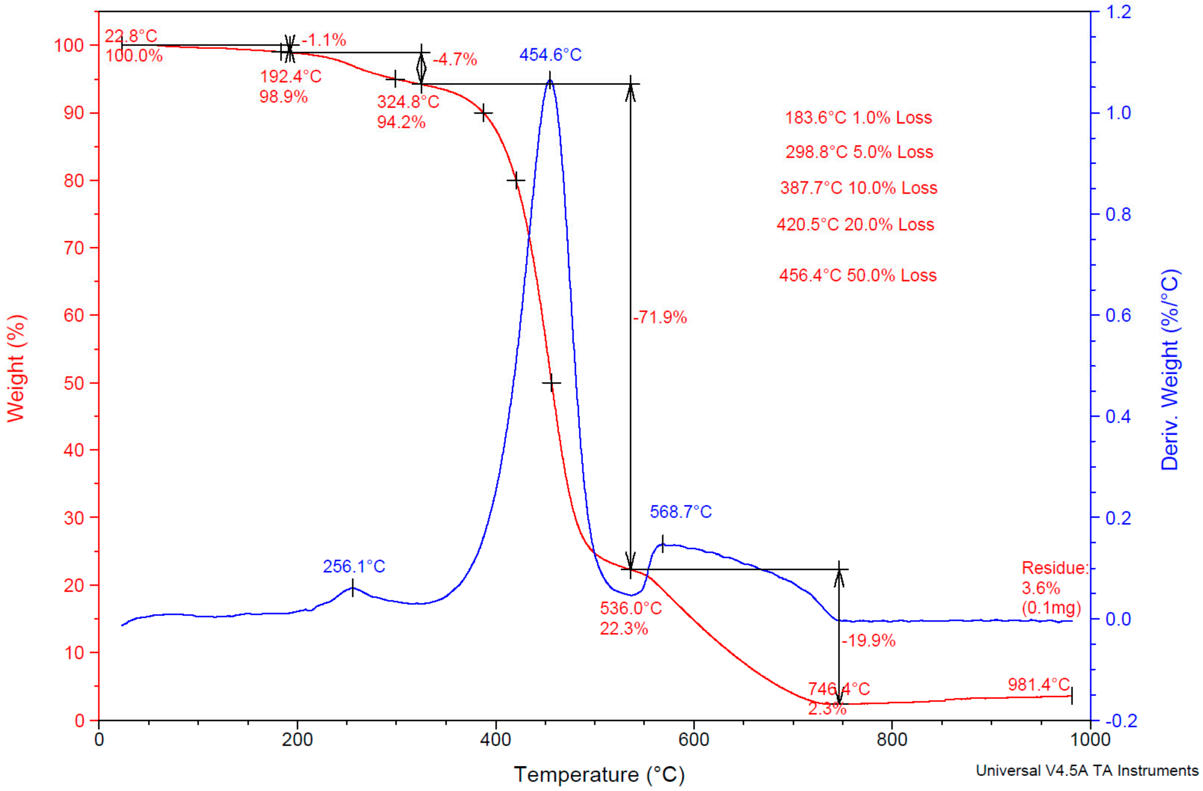

Figure S52.TGA analysis of polymer **P1**

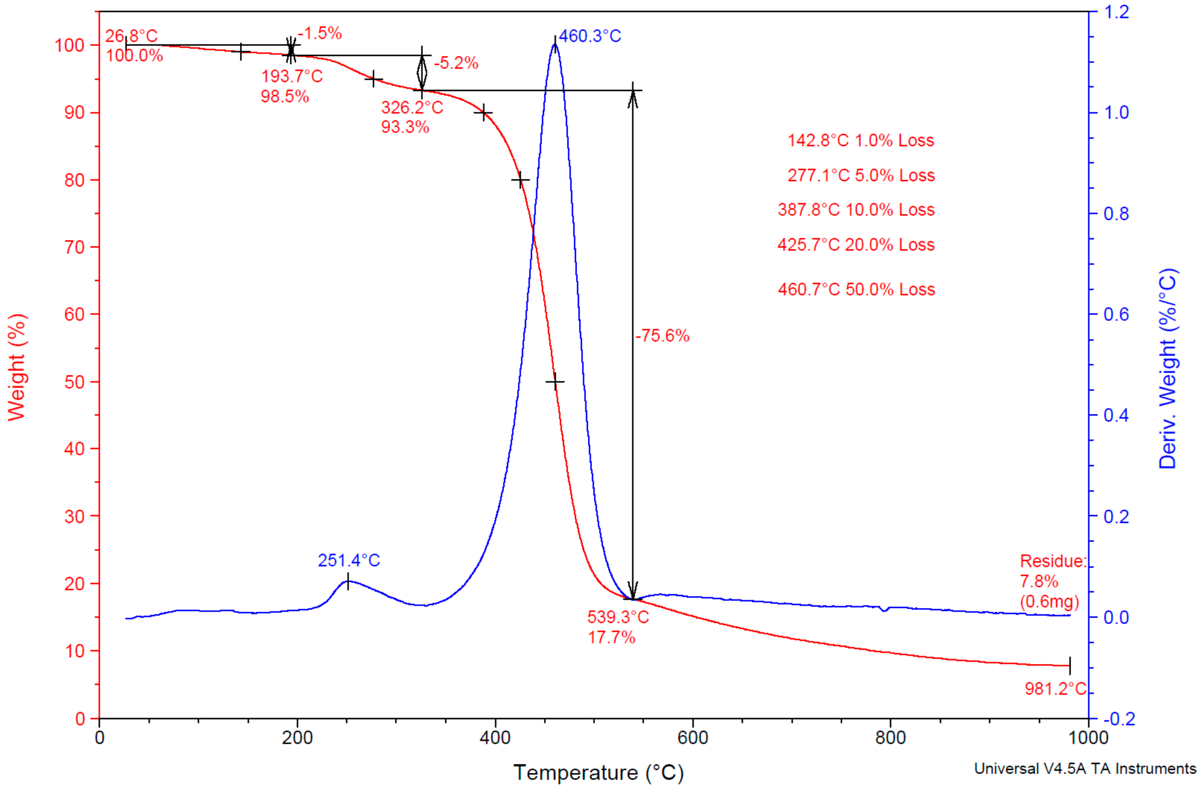

Figure S53. TGA analysis of polymer **P3**

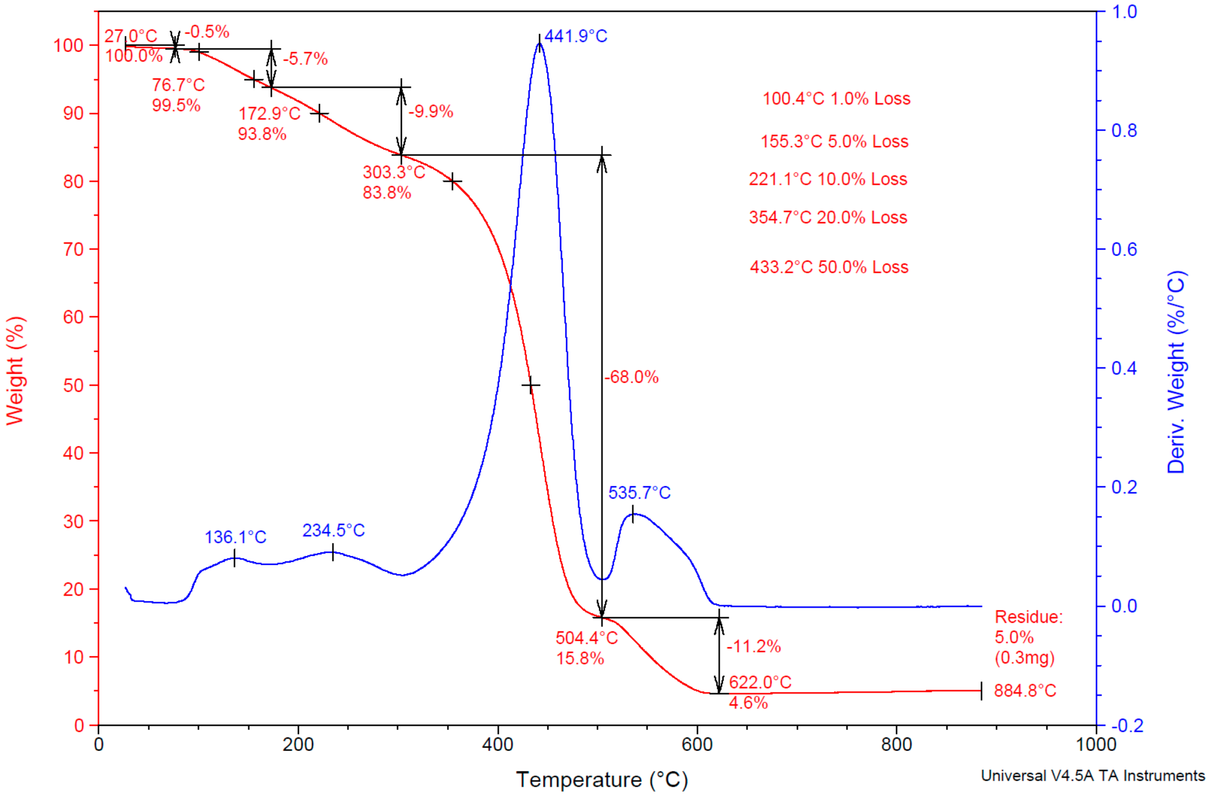

Figure S54. TGA analysis of polymer **P4**

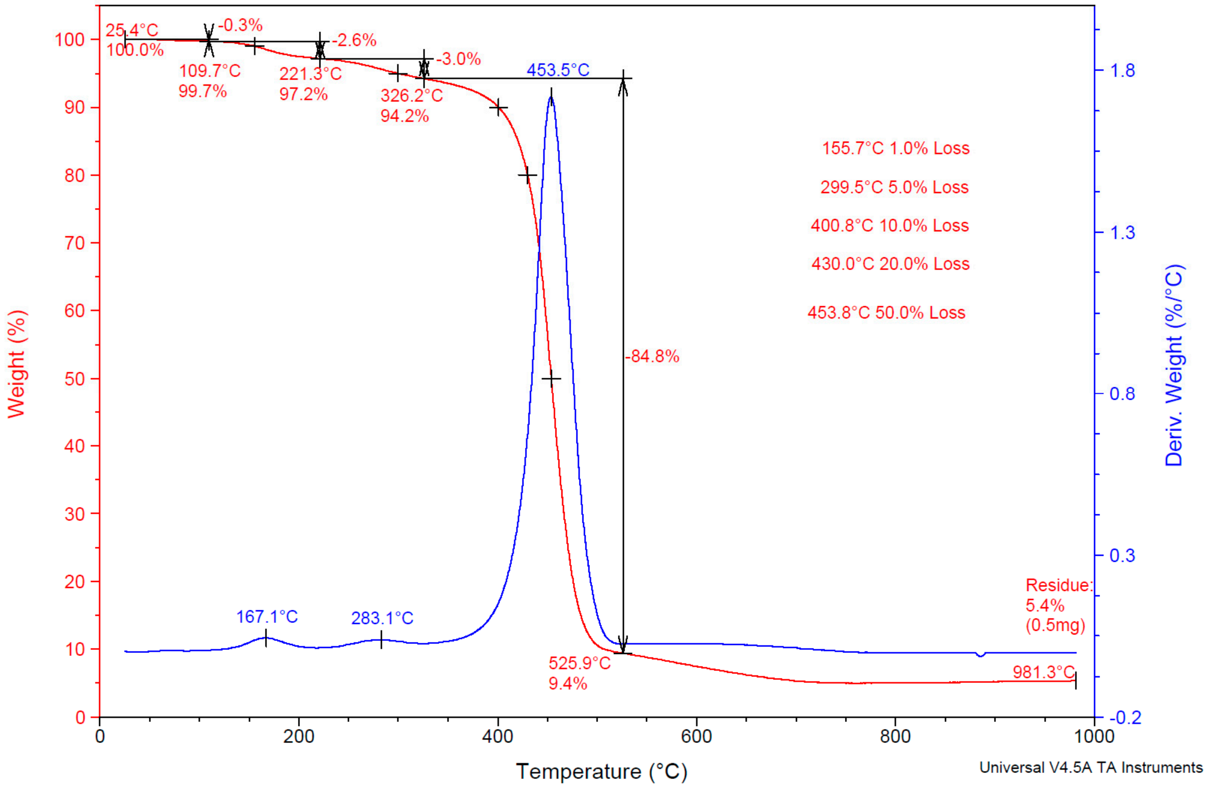

Figure S55. TGA analysis of polymer **P5**

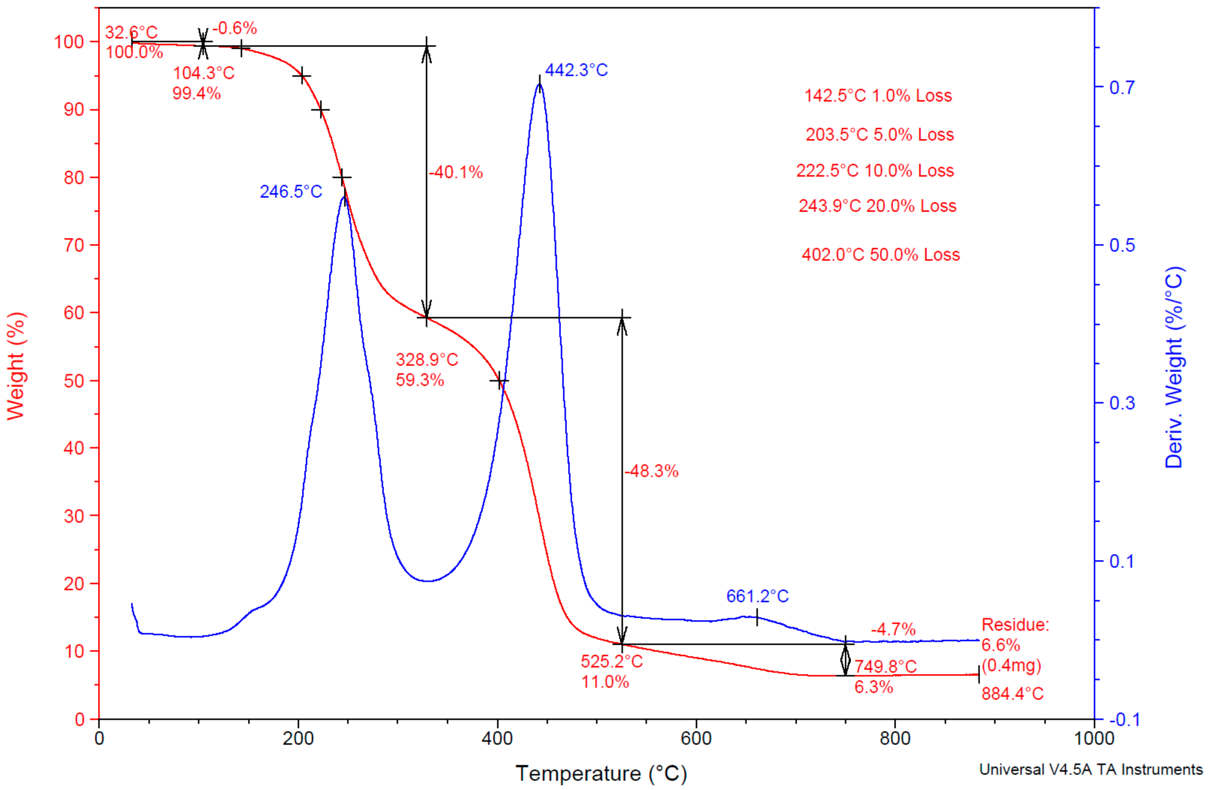

Figure S56. TGA analysis of polymer **P6**

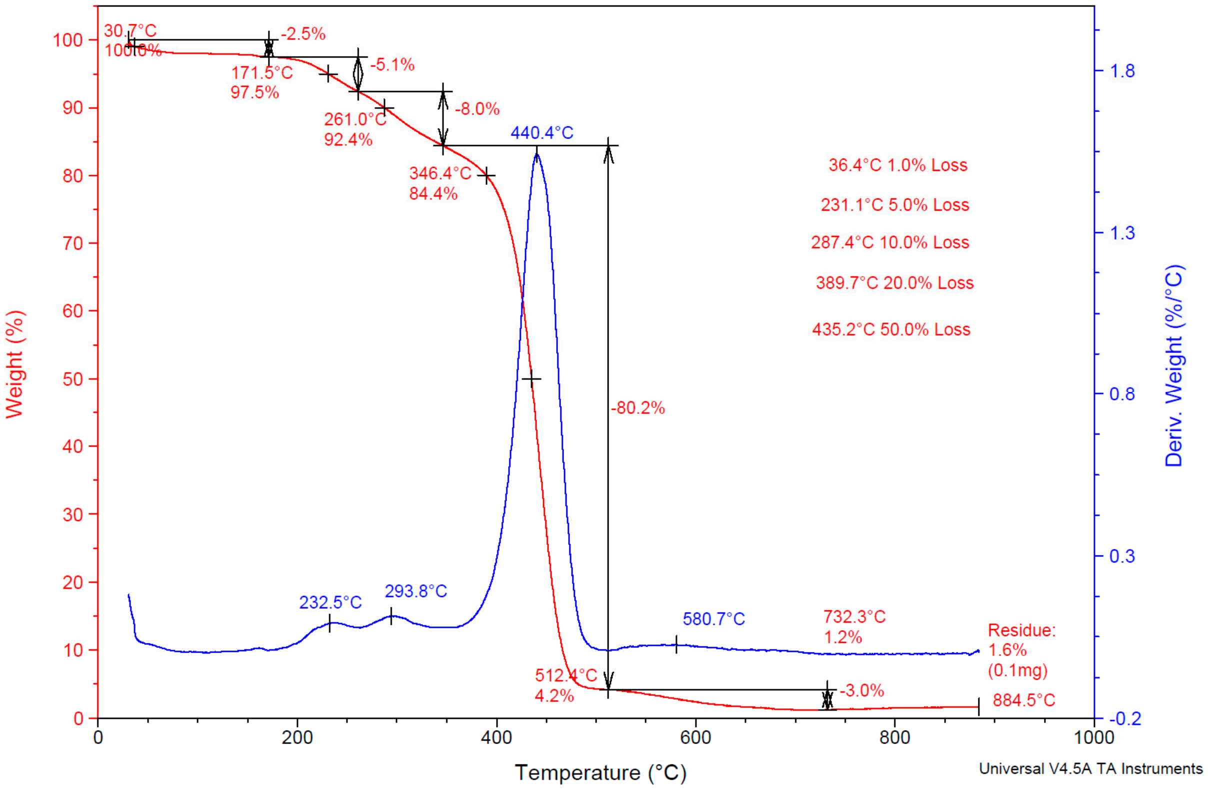

Figure S57. TGA analysis of polymer **P7**

SEM Images

POWDERS

Polymer P5

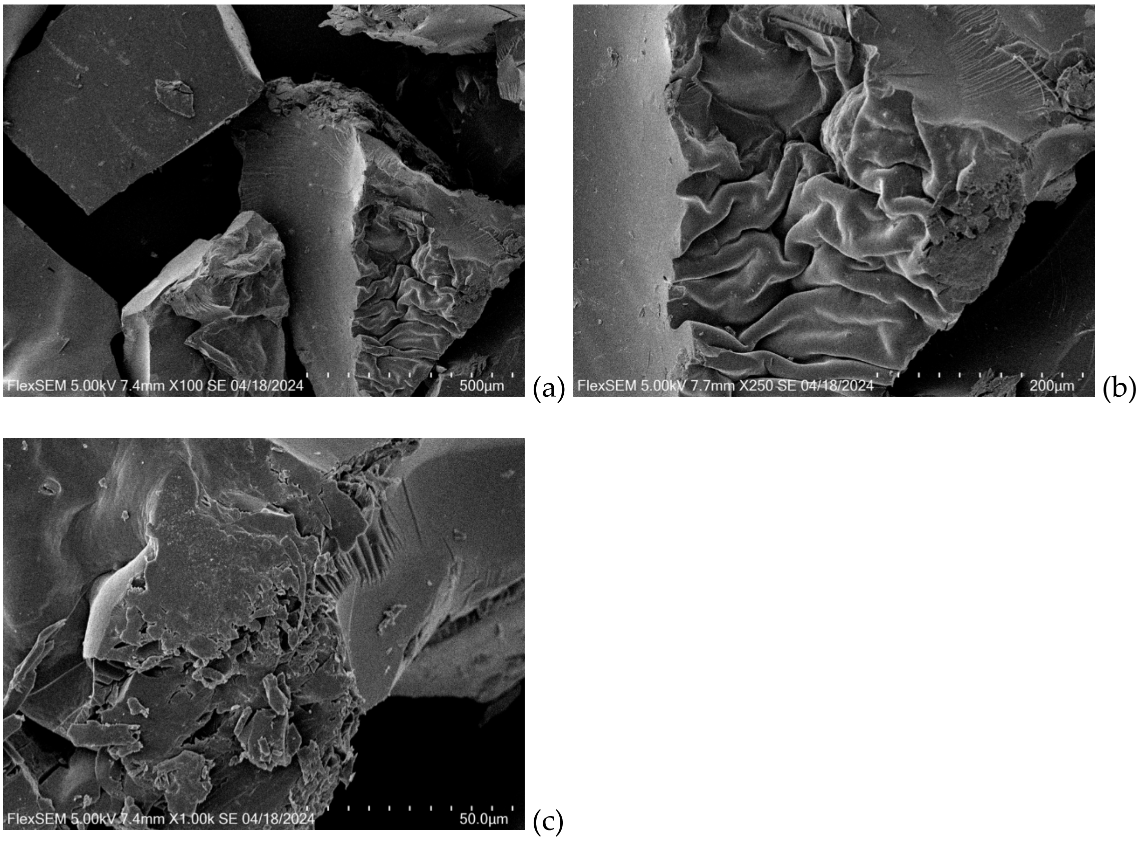

Figure S58. SEM images of polymer P5 surfaces as a powder: a) 100 x magnification; b) 250 x magnification; c) 1000 x magnification.

The polymer P5 is structurally characterized by a large-block-type material devoid of pores. The items in question can be described as 'small plates'. If we direct our attention to the lower right corner of the photograph, we may observe irregular fibers providing a sense of general structure. It appears that certain particles have undergone a process of stretching or collapse, resulting in the formation of irregular cavities that bear a resemblance to fossils (Fig. S53a). The second picture (Fig. S53b) focuses on these 'fossils', 'cavities' or 'scars'. In the upper right quadrant, the scars appear to have undergone a process of flattening, forming a pattern that resembles the topography of mountain peaks and a descending slope. The last magnification (Fig.S53c) of the middle right torn structure reveals different levels of layered material, but no pores are present. Cracks are also visible.

FILMS

The polymeric material films exhibit a high degree of homogeneity, with only minor impurities present. Furthermore, cracks become discernible at all higher magnifications. Nevertheless, the films possess a smooth surface. However, no pores or pore-lined areas are visible.

Polymer P1

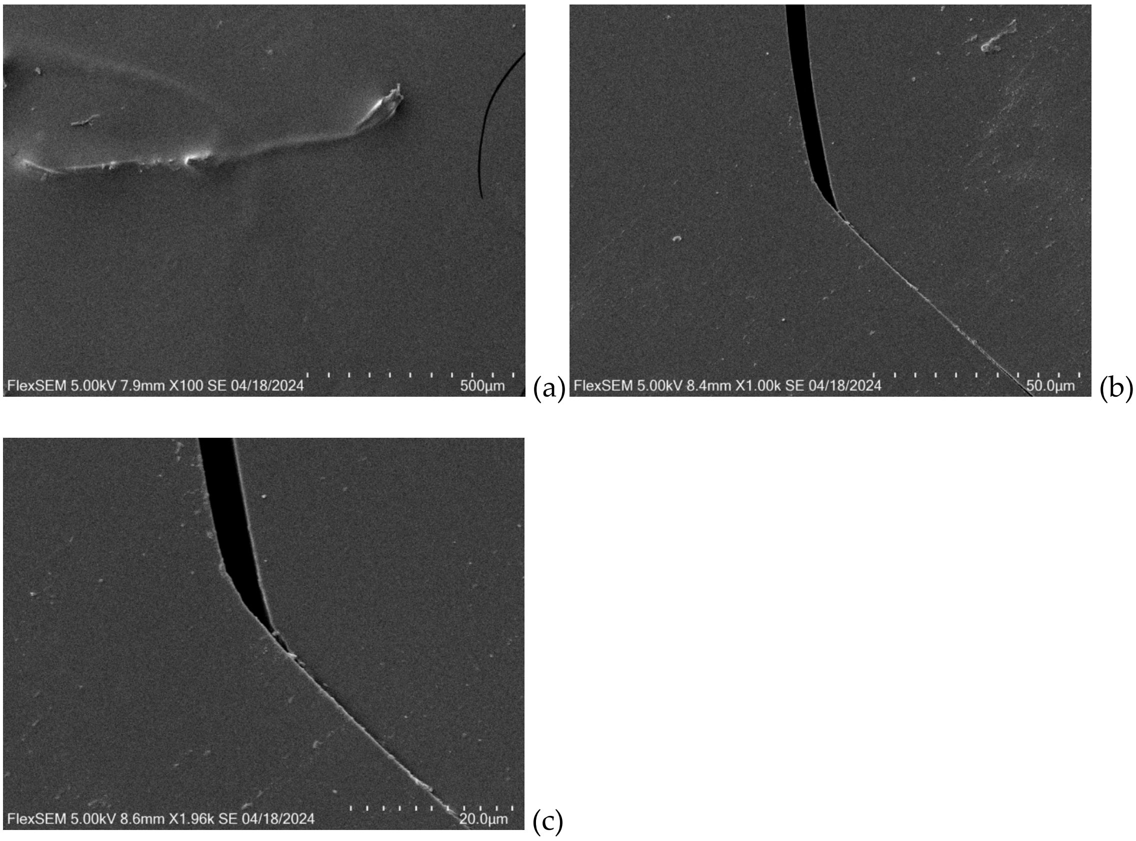

Figure S59. SEM images of polymer P1 surfaces as a film: a) 100 x magnification; b) 1000 x magnification; c) 2000 x magnification.

Polymer P3

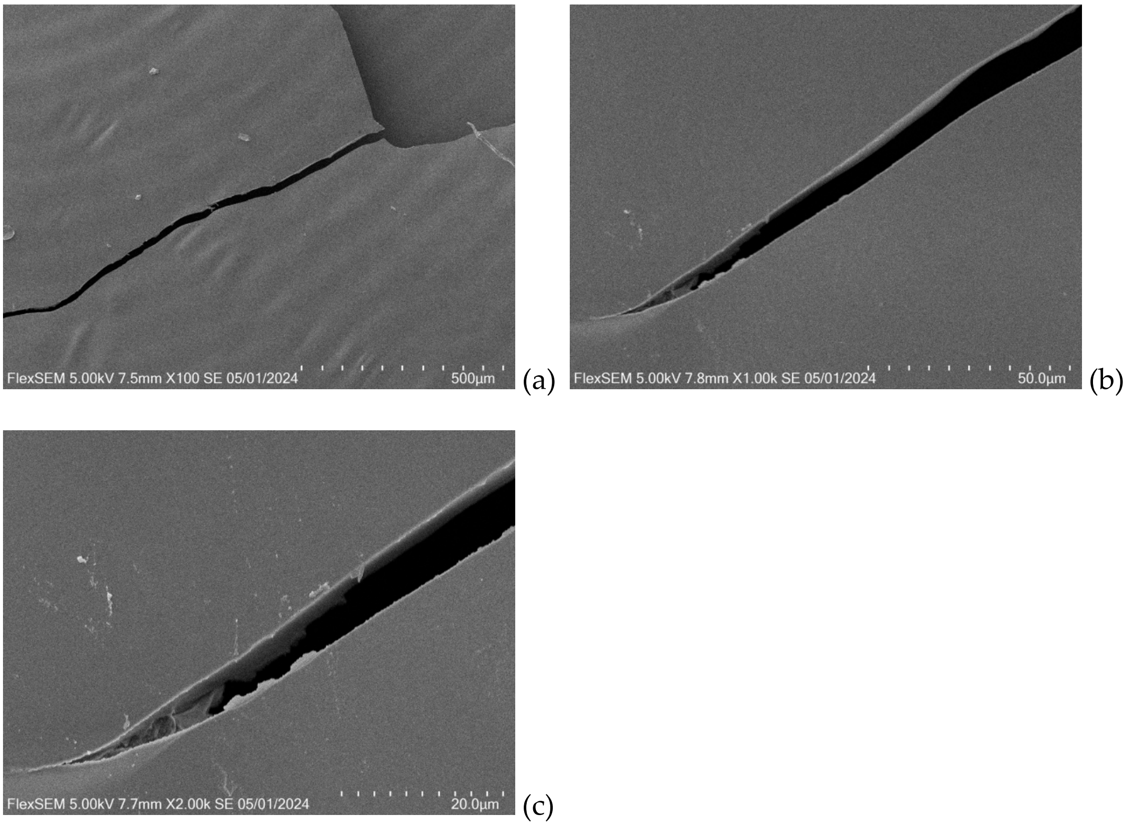

Figure S60. SEM images of polymer P3 surfaces as a film: a) 100 x magnification; b) 1000 x magnification; c) 2000 x magnification.

Polymer P5

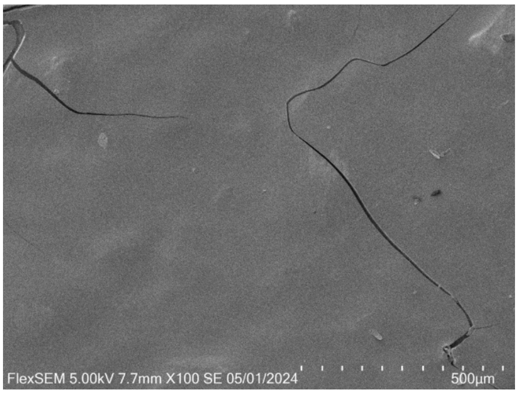

(a)

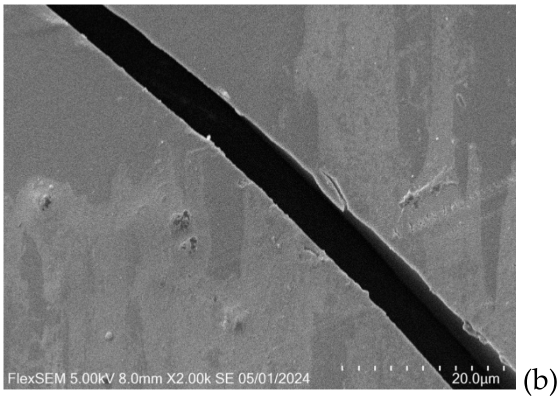

(b)

Figure S61. SEM images of polymer P5 surfaces as a film: a) 100 x magnification; b) 2000 x magnification.

Polymer P8

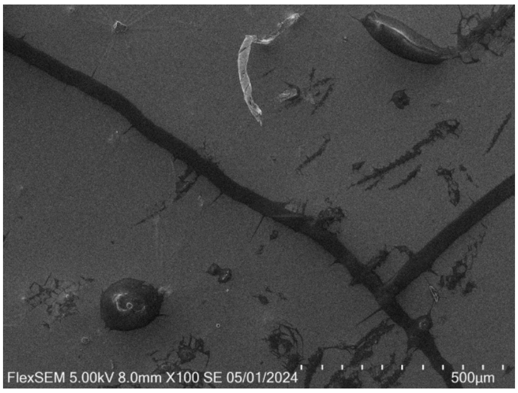

(a)

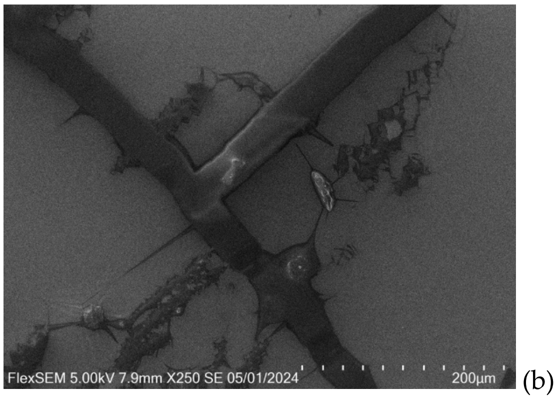

(b)

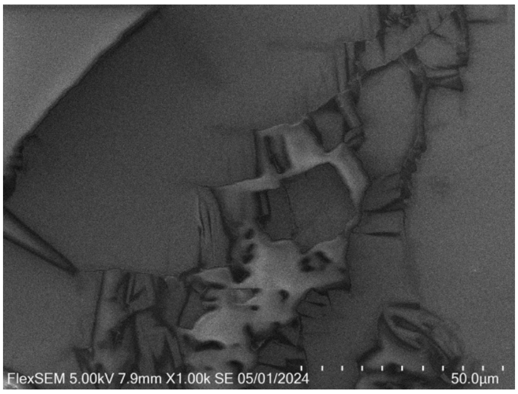

(c)

Figure S62. SEM images of polymer P8 surfaces as a film: a) 100 x magnification; b) 250 x magnification; c) 2000 x magnification.

LIBS Analysis and Images: In all tested samples (P1, P3, P7), no ruthenium was detected above the LOD (0.03% wt.).

Polymer P1

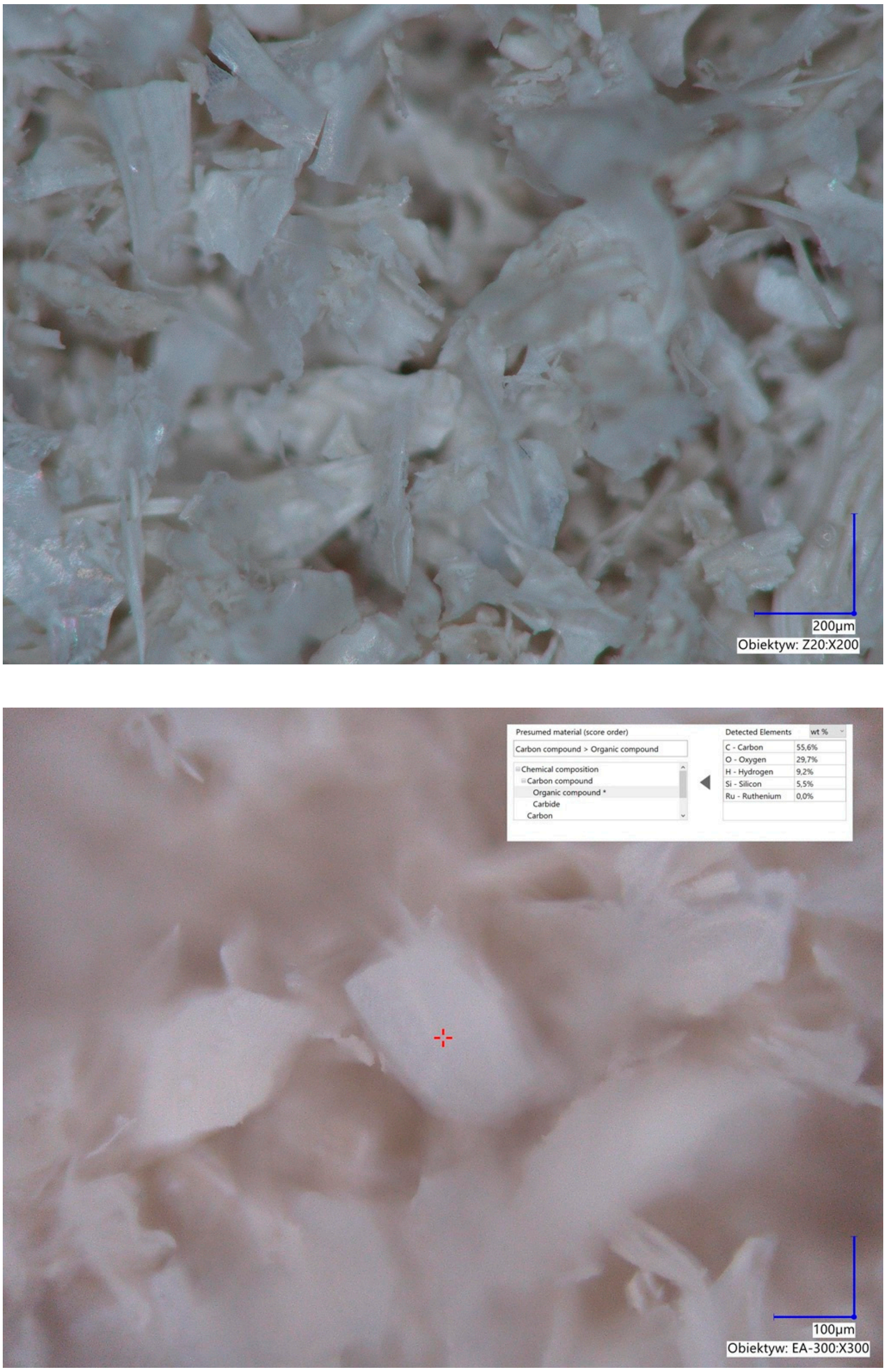

(a)

(b)

Figure S63. LIBS/ICP images of polymer P1 surfaces: a) microscopic image; b) microscopic image and LIBS measurement

Polymer P3

(a) 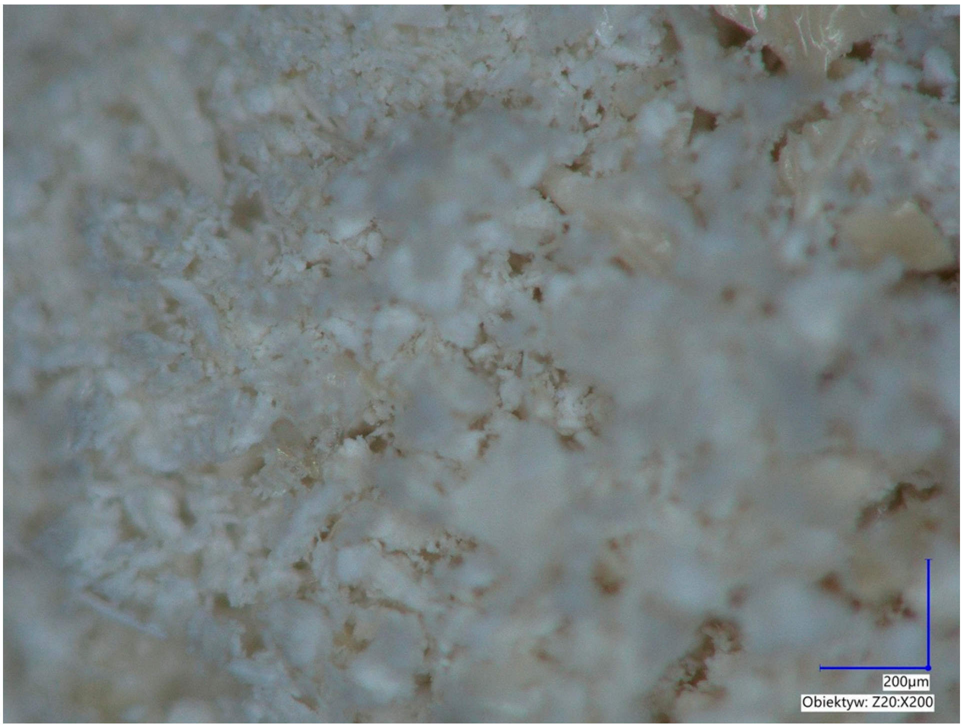

(b) 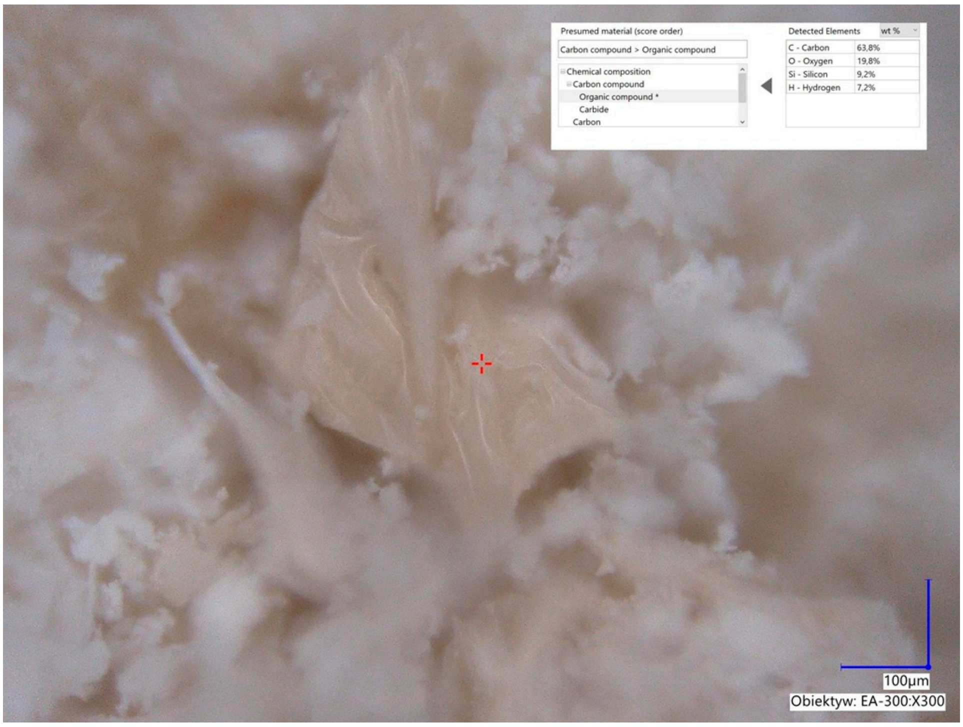

Figure S64. LIBS/ICP images of polymer P3 surfaces: a) microscopic image; b) microscopic image and LIBS measurement

Polymer P7

(a)
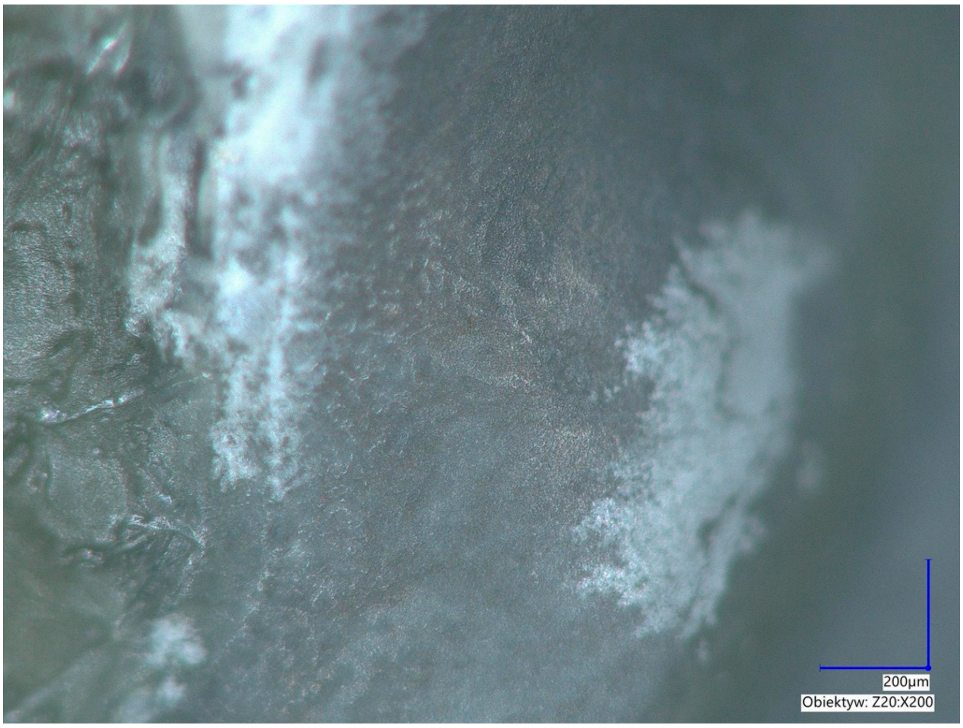

(b)
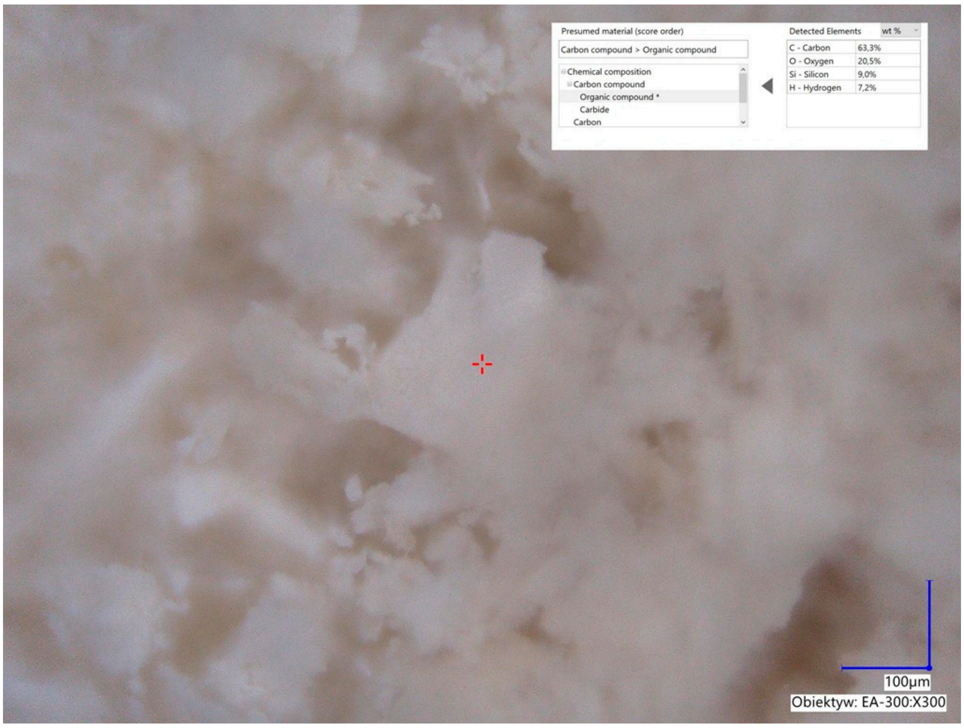

Figure S65. LIBS/ICP images of polymer P7 surfaces: a) microscopic image; b) microscopic image and LIBS measurement

References

1. B.S. Furniss; A.J. Hannaford; P.W.G. Smith; A.R. Tatchell, *A. I. VOGEL's Textbook of Practical Organic Chemistry*, 5 Edition, Longman Scintific & Technical, John Wiley & Sons., Inc. New York, 1989.
